# Supplementary material for: Decreased blood vessel density and endothelial cell subset dynamics during ageing of the endocrine system
Source: EMBO J. 2020 Nov 20;40(1):e105242. doi: 10.15252/embj.2020105242 (PMC7780152; doi:10.15252/embj.2020105242)
Supplement: Supplementary file 1 — Appendix [file EMBJ-40-e105242-s001.pdf]

**APPENDIX**

**Single-cell resolution 3D imaging reveals dysregulation of vasculature in ageing of the endocrine system**

**Table of contents**

1. Appendix Figure S1 ………………………………………………………. Page 2
2. Appendix Figure S2 ………………………………………………………. Page 3
3. Appendix Figure S3 ………………………………………………………. Page 4
4. Appendix Figure S4 ………………………………………………………. Page 5
5. Appendix Figure S5 ………………………………………………………. Page 6
6. Appendix Figure S6 ………………………………………………………. Page 7
7. Appendix Figure S7 ………………………………………………………. Page 8
8. Appendix Table S1 ……………………………………………………….. Page 9
9. Appendix Table S2 ………………………………………………………. Page 12
10. Appendix Table S3 ………………………………………………………. Page 16
11. Appendix Table S4 ………………………………………………………. Page 18
12. Appendix Table S5 ………………………………………………………. Page 24
13. Appendix Table S6 ………………………………………………………. Page 26
14. Appendix Table S7 ………………………………………………………. Page 33
15. Appendix Table S8 ………………………………………………………. Page 41
16. Appendix Table S9 ………………………………………………………. Page 42
17. Appendix Table S10 …………………………………………………….. Page 46
18. Appendix Table S11 …………………………………………………….. Page 48
19. Appendix Table S12 …………………………………………………….. Page 53

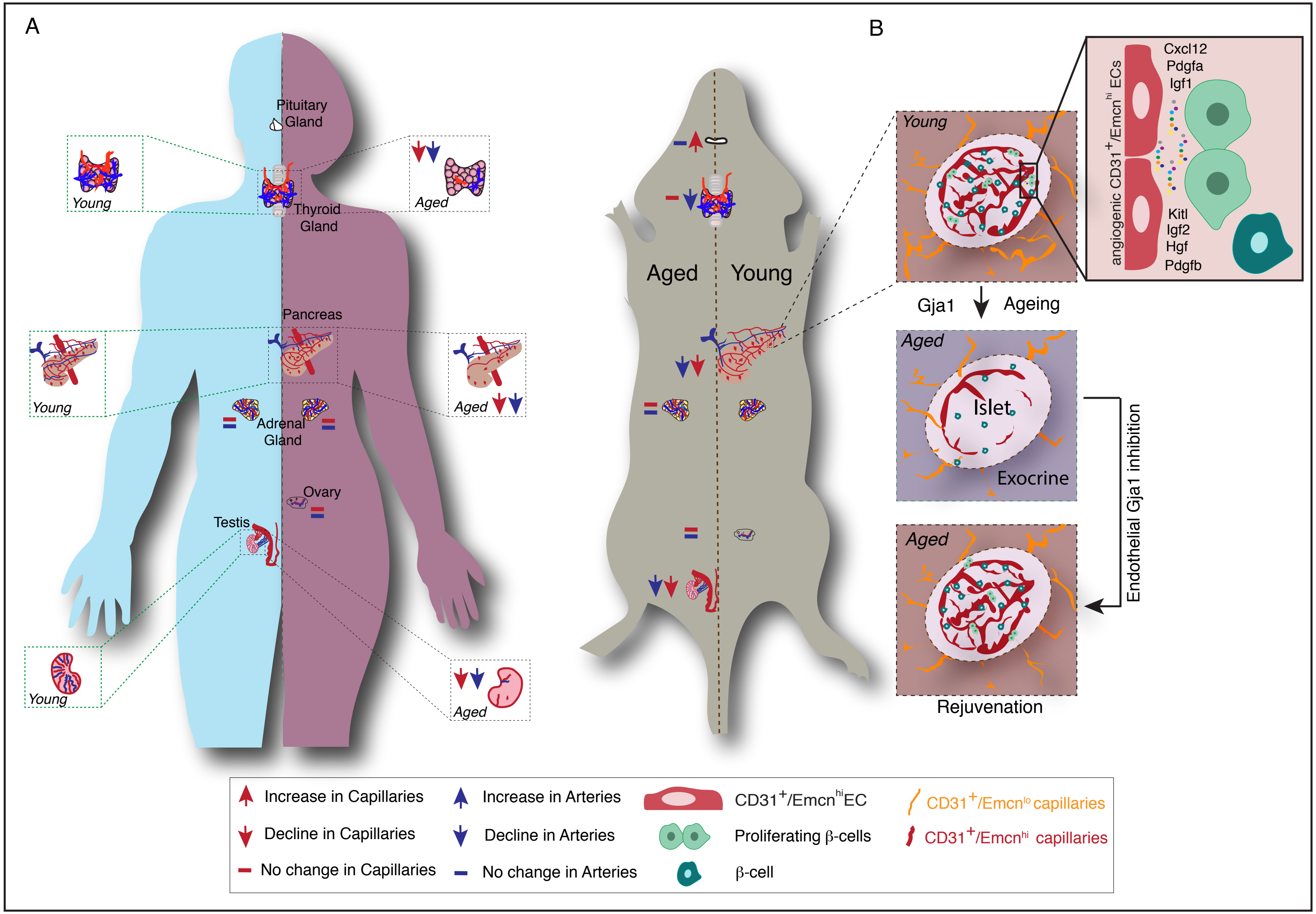


**Appendix Figure S1** Schematic representation of vascular perturbations in the ageing of the endocrine glands with details on age-dependent loss of specialized capillaries in pancreas and impact on β-cell proliferation.

**A** Age-related changes in the vasculature of human and murine endocrine glands. Human pancreas, testis and thyroid gland demonstrated an age-dependent decline in microvascular density and arterial numbers. Also, arterial numbers declined in aged murine pancreas, testis and thyroid gland.

**B** Angiogenic CD31$^{+}$ Emcn$^{hi}$ capillaries abundant in young pancreatic islets secrete factors to promote β-cell survival and proliferation. These CD31$^{+}$ Emcn$^{hi}$ capillaries exhibit age-dependent decline leading to a decrease in β-cell numbers and proliferation. Endothelial Gja1 negatively regulates angiogenesis and thereby β-cell proliferation. Endothelial Gja-1 inhibition reactivated angiogenesis and CD31$^{+}$ Emcn$^{hi}$ ECs in aged mice and boosted β-cell proliferation, rejuvenating the aged pancreatic islet.

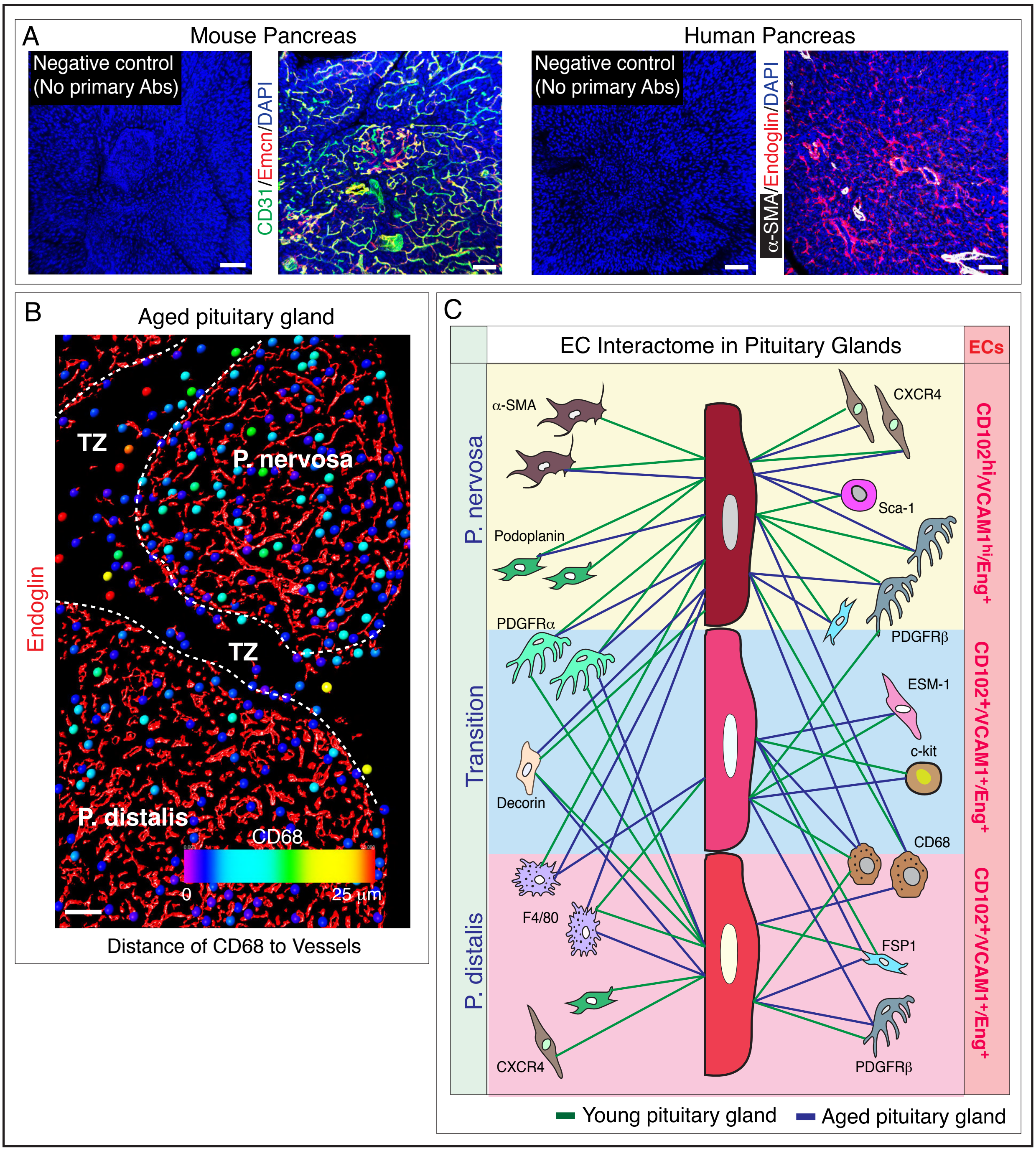


**Appendix Figure S2** Negative controls of immunostaining and cell-cell interactome analysis in young and aged pituitary gland

**A** Negative control with no primary antibodies and representative 3D images show the CD31 and Emcn immunostaining in mouse frozen pancreas tissue, α-SMA and Endoglin immunostaining in paraffin embedded human pancreas tissue.
**B** Exemplar image illustrates the cell-cell interactome analysis with the distance estimates of CD68$^+$ macrophages to blood vessels (Endoglin) in P. nervosa, P. distalis and the transition zone (TZ) of an aged pituitary gland. The colour code indicates the distance which is very close in violet and very far in red. The scale bar is 50 $\mu$m. The dashed lines represent the outline of the transition zone.
**C** Illustration of the EC interactome in pituitary glands and changes in the interactome with ageing. Analysis of endothelium in the different region showed that they have distinct marker expressions. CD102$^{hi}$/VCAM-1$^{hi}$/Endoglin$^+$, CD102$^+$/VCAM-1$^+$/Endoglin$^+$ and CD102$^+$/VCAM-1$^+$/Endoglin$^+$ ECs were located in P. nervosa, transition zone (TZ) and P. distalis regions respectively in adrenal glands from young and aged mice. Their interactions in young and aged adrenal glands with PDGFRβ$^+$ pericytes, PDGFRα$^+$ mesenchymal cells, α-SMA$^+$ pericytes, Decorin+ stromal cells, CXCR4$^+$ stromal cells, FSP1$^+$ fibroblasts, Podoplanin$^+$ fibroblasts, CD34$^+$ hematopoietic cells, ESM-1$^+$ tip cells, c-kit$^+$ stem cells, Sca-1$^+$ stem cells, F4/80$^+$ macrophages and CD68$^+$ macrophages are shown.

Data information: Nuclei: DAPI. Scale bars are 50 $\mu$m for all the 3D images.

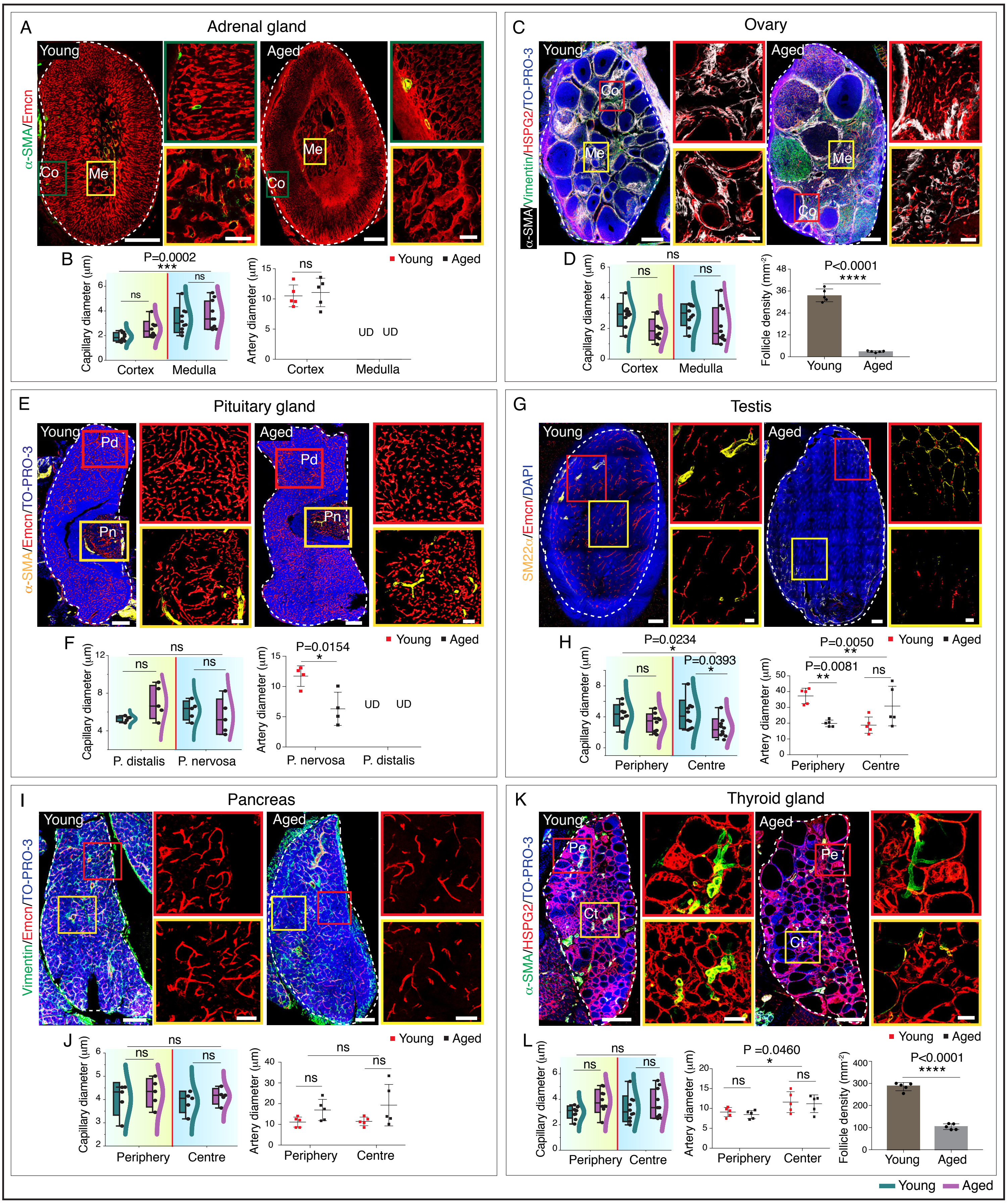


**Appendix Figure S3** The decline of arteries in the ageing of endocrine glands

**A** 3D images with α-SMA and Emcn in young and aged adrenal gland. Insets show cortex and medulla regions of the adrenal gland.
**B** Combined box and whiskers, and scatter plot (left) showing the quantification of capillary diameter in young and aged adrenal glands. Graph (right) shows the changes in artery diameter. Data represent mean ± s.d. (capillary diameter n=10; artery diameter n=5), P-value, two-tailed unpaired t-test (artery diameter) and one-way ANOVA tests with Tukey's multiple comparisons tests (capillary diameter).
**C** 3D images of young and aged ovary staining with α-SMA, Vimentin and HSPG2. Insets show cortex and medulla regions of the ovary.
**D** Combo plot (left) shows the quantification of capillary diameter in young and aged ovaries. Graph shows the follicle density in young and aged ovaries. Data represent mean ± s.d. (capillary diameter n=10; follicle density n=5), P-value, two-tailed unpaired t-test (follicle density) and one-way ANOVA tests with Tukey's multiple comparisons tests (capillary diameter).
**E** Representative tile scan 3D images with α-SMA and Emcn in young and aged pituitary gland. Insets show higher magnification of pars distalis and pars nervosa of the pituitary gland.
**F** Combo plot (left) shows the quantification of capillary diameter. Graph (right) shows the changes in artery diameter in young and aged pituitary gland. Data represent mean ± s.d. (capillary diameter n=5; artery diameter n=4), P-value, two-tailed unpaired t-test (artery diameter) and one-way ANOVA tests with Tukey's multiple comparisons tests (capillary diameter).
**G** 3D images of young and aged testis staining with SM22α and Emcn.
**H** Combo plot (left) shows the quantification of capillary diameter in young and aged testis. Graph (right) shows the quantification of artery diameter. Data represent mean ± s.d. (capillary diameter n=10; artery diameter n=5), P-value, and one-way ANOVA test with Tukey's multiple comparisons test.
**I** Representative tile scan 3D images of young and aged pancreas with staining of Vimentin and Emcn.
**J** Combo plot (left) shows the quantification of capillary diameter. Graph (right) shows quantification of artery diameter in periphery and centre regions of young and aged pancreas. Data represent mean ± s.d. (n=5), one-way ANOVA test with Tukey's multiple comparisons test.
**K** Representative tile scan and crops of periphery and centre 3D images show α-SMA and HSPG2 in young and aged thyroid gland.
**L** Combo plot (left) shows the quantification of capillary diameter in young and aged thyroid gland. Bar graphs (middle and right) show the quantifications of artery diameter and follicle density. Data represent mean ± s.d. (capillary diameter n=10; artery diameter and follicle density n=5), P-values, one-way ANOVA with Tukey's multiple comparisons tests (capillary and artery diameter) and two-tailed unpaired t-tests (follicle density).

Data information: The white dashed lines in Panel A, C, E, G, I and K represent the outlines of organs. One-way ANOVA with Tukey's multiple comparisons tests were performed for statistical analysis between more than two groups; two-tailed Student's t-test was applied for analysis between two groups. ns: not significant; UD: undetermined; *: P < 0.05; **: P < 0.01; ***: P < 0.001; ****: P < 0.0001. Nuclei: DAPI or TO-PRO-3 as indicated. Co: Cortex; Me: Medulla; Pe: Periphery; Ct: Centre; Pd: Pars Distalis; Pn: Pars Nervosa. For the Combo plots, the boxes represent mean± s.d., line in the box is the median, and the lower and upper lines show the minimum and the maximum of the values. The line on the right side of these combo plots in represents the sample distribution. Scale bars are 200 μm for tile scan 3D images and 50 μm for the high magnification insets.

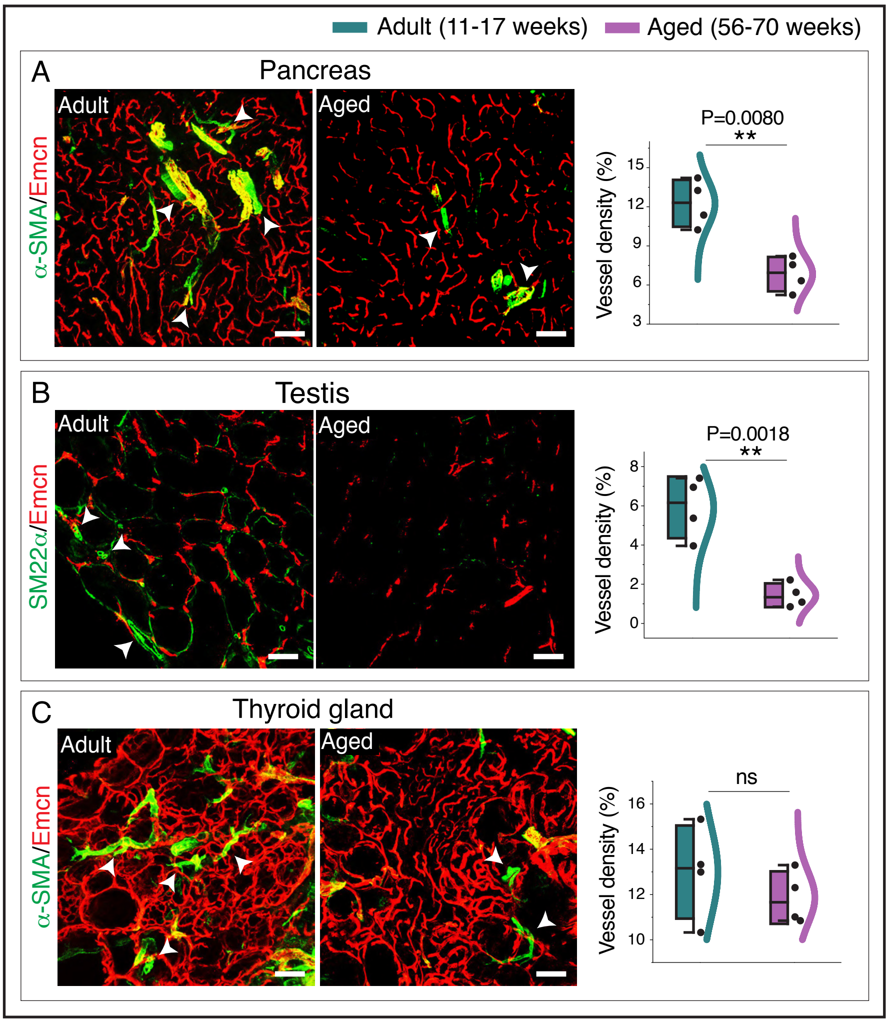


**Appendix Figure S4** Vascular analysis in murine adult and aged endocrine glands

**A** Representative 3D images with staining of α-SMA and Emcn in adult and aged pancreas. Combined box and whiskers, and scatter plot shows the quantification of the vessel density in adult and aged pancreas. Arrowheads indicate arteries.
**B** 3D images from adult and aged testis staining with SM22α and Emcn. Combo plot shows the quantification of vessel density. Arrowheads indicate arteries.
**C** Representative 3D images show α-SMA and Emcn immunostaining in adult and aged thyroid gland. Combo plot shows the quantification of vessel density. Arrowheads indicate arteries.

Data information: (n=4), P-values, and two-tailed unpaired t-tests for all the above panels. ns: not significant; **: $P < 0.01$. Mice at ages 11-17 weeks and 56-70 weeks were chosen for adult and aged group sets for all the above panels. The line on the right side of the combo plots represents the sample distribution. Scale bars are 50 $\mu$m for all the 3D images.

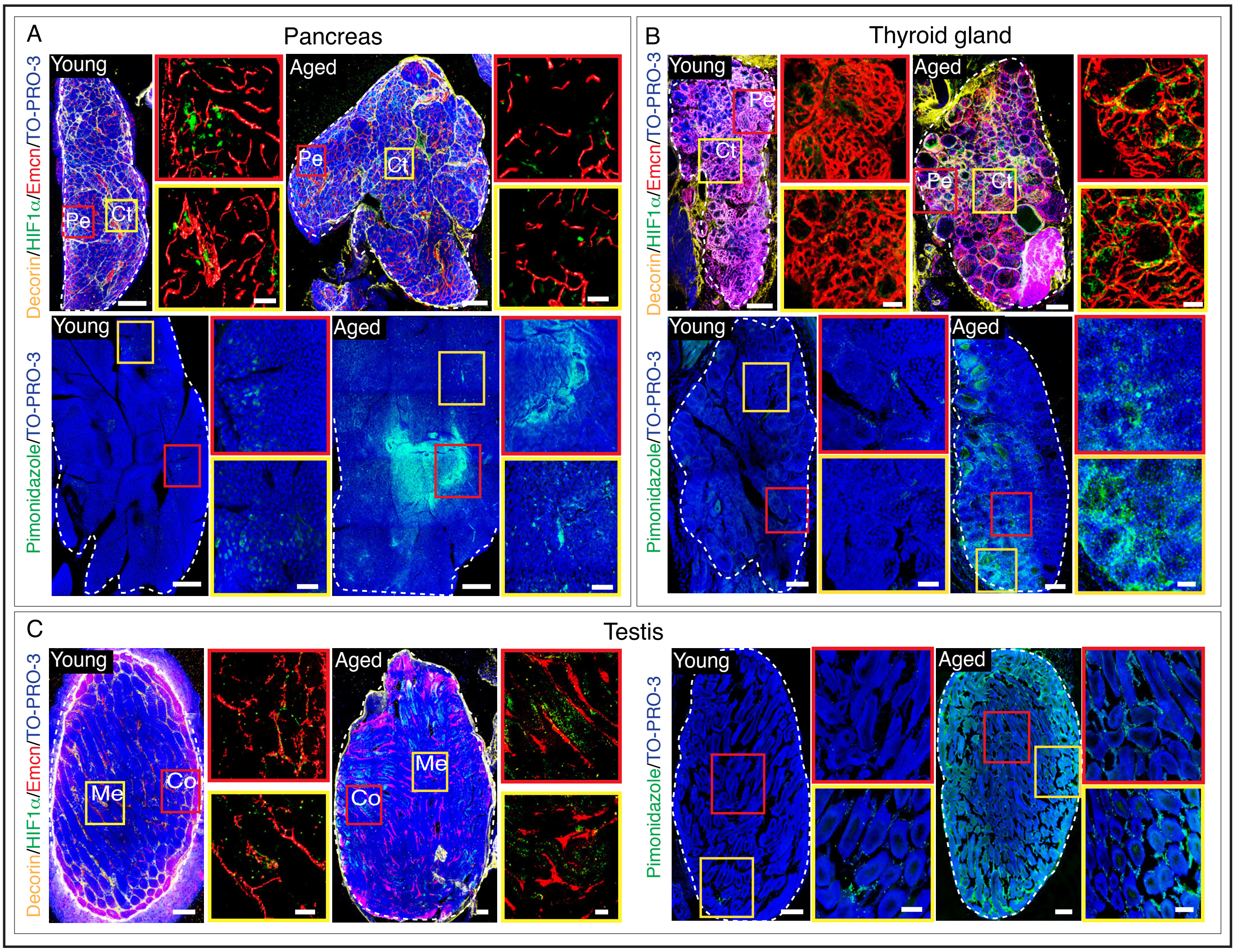


**Appendix Figure S5** Hypoxic nature of the aged endocrine glands

**A** 3D images on the top of panel with the immunostaining of Decorin, HIF1α and Emcn on young and aged pancreas in periphery and centre regions. Images at the bottom of panel shows the hypoxia regions probed by Pimonidazole in young and aged pancreas. Insets show higher magnification regions.
**B** Representative 3D images (top panel) show Decorin, HIF1α and Emcn in thyroid glands from young and aged mice. Bottom images show the hypoxia regions probed by Pimonidazole in young and aged thyroid gland.
**C** Representative 3D images (left panel) with young and aged testis stained with Decorin, HIF1α and Emcn in cortex and medulla regions. Images (right panel) show the hypoxia regions probed by Pimonidazole in young and aged testis.

Data information: The white dashed lines in each panel represent the outlines of organs. Nuclei: TO-PRO-3. Co: Cortex; Me: Medulla; Pe: Periphery; Ct: Center. Scale bars are 200 $\mu$m for tile scan 3D images and 50 $\mu$m for the high magnification insets.

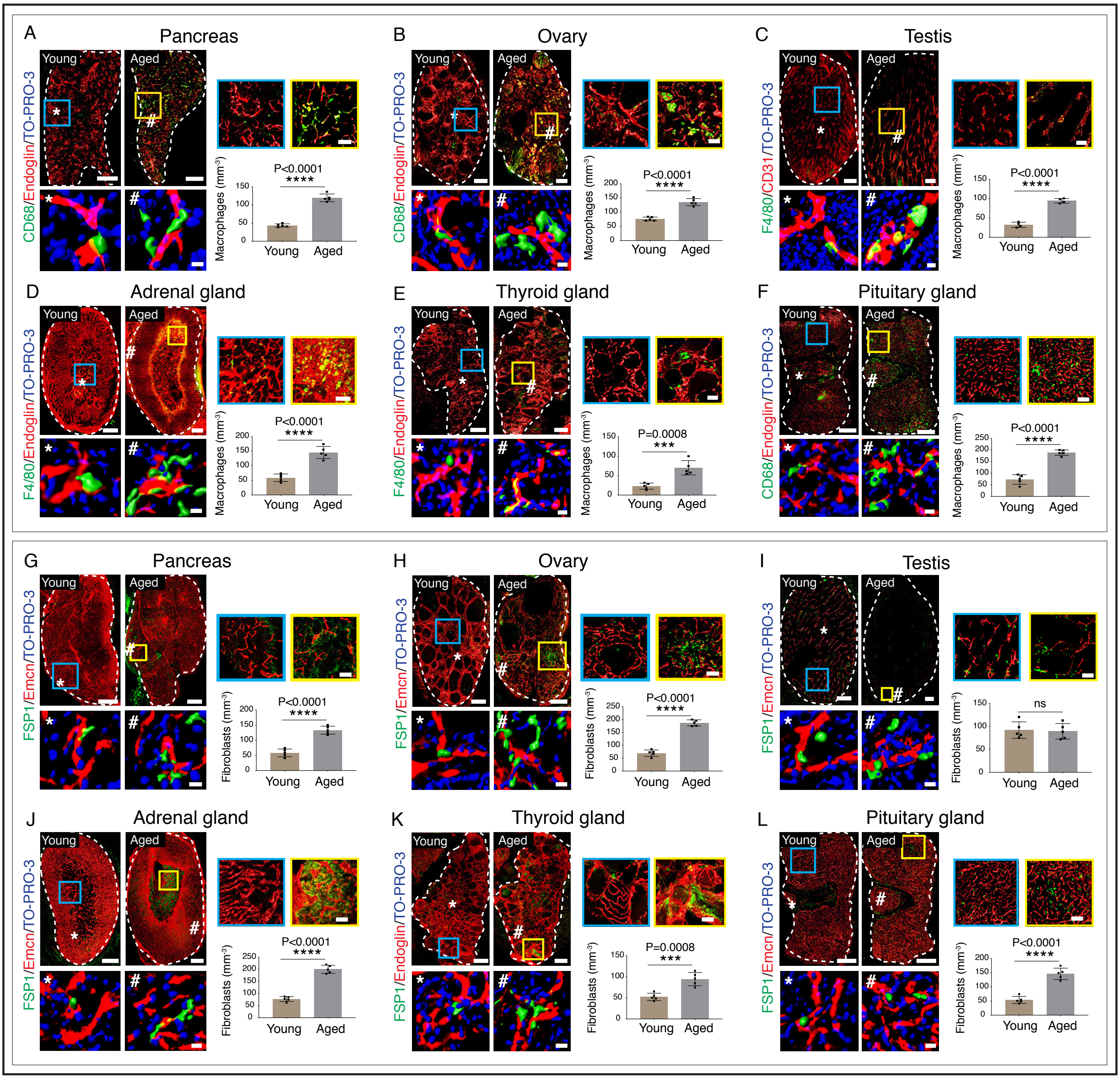


**Appendix Figure S6** Age-associated expansion of perivascular macrophages and fibroblasts in endocrine glands

**A-F** Representative 3D images and insets show pancreas, ovary, testis, adrenal gland, thyroid gland and pituitary gland from young and aged mice stained with CD68 and Endoglin, F4/80 and Endoglin or F4/80 and CD31. Nuclei stained with TO-PRO-3. The insets on the top right part of each panel with higher magnification of macrophages in different regions of the glands. At the bottom left panels insets (* and #) show the single-cell resolution topography of the macrophages which are in close proximity with the blood vessels. Bar graphs show the quantifications of macrophage numbers per tissue volume (1 mm$^3$) based on CD68 immunostaining. Data represent mean ± s.d. (testis n=4; other glands n=5), P-values, and two-tailed unpaired t-tests.

**G-L** Representative tile scan 3D images and higher magnifications insets show FSP1$^+$ fibroblasts and blood vessels stained with Emcn or Endoglin in pancreas, ovary, testis, adrenal gland, thyroid gland and pituitary gland from young and aged mice. Nuclei stained with TO-PRO-3. Insets on the top right of each panel show higher magnification regions of FSP1$^+$ fibroblasts for each gland. Insets at the bottom left of each panel show higher magnification of single-cell resolution topography of the FSP1$^+$ fibroblasts which are in close proximity with blood vessels. Bar graphs show the quantifications of FSP1$^+$ fibroblast numbers per 1 mm$^3$ of sample volume counted on thick endocrine sections of each young and aged endocrine gland. Data represent mean ± s.d. (n=5), P-values, and two-tailed unpaired t-tests.

Data information: The white dashed lines in each panel represent the outlines of organs. Two-tailed Student's t-test was applied for the statistical analysis. ns: not significant; **: P <0.01; ***: P <0.001; ****: P <0.0001. Scale bars are 200 $\mu$m for tile scan 3D images and 50 $\mu$m for the high magnification insets and 5 $\mu$m for the single-cell resolution images.

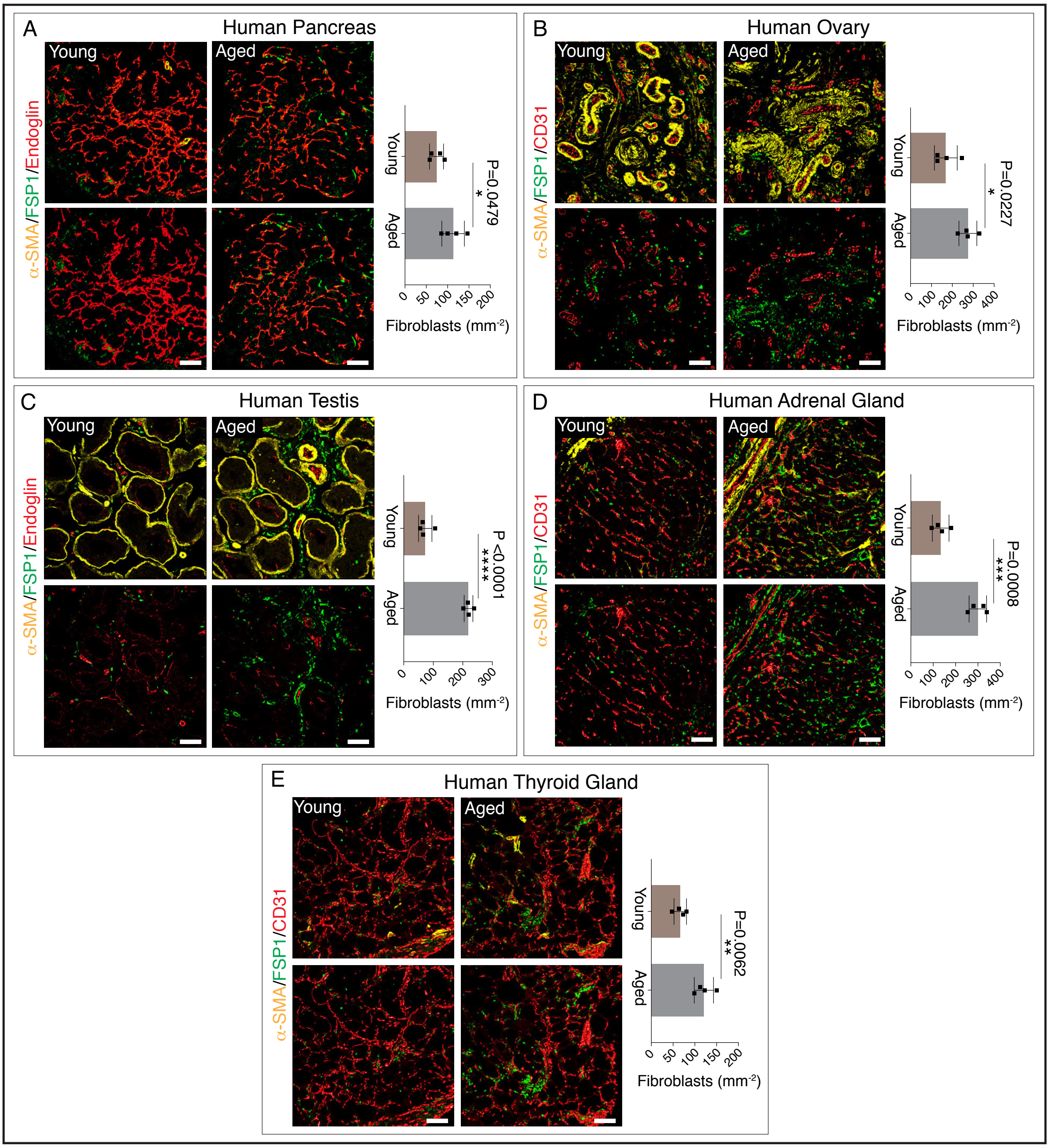


**Appdenix Figure S7** Age-associated expansion of fibroblasts in human endocrine tissues

**A** 3D images show α-SMA, FSP1 and Endoglin in young and aged human pancreas. The graph shows the quantification of the number of FSP1⁺ fibroblasts per 1 $mm^2$ sample area counted on thick sections of young and aged pancreas. Data represent mean ± s.d. (n=4), P-value, and two-tailed unpaired t-test.
**B** Representative 3D images with α-SMA, FSP1 and CD31 immunostaining in young and aged human ovary. Graph with the quantification of $FSP1^+$ fibroblast numbers per 1 $mm^2$ sample area in young and aged human ovary. Data represent mean ± s.d. (n=4), P-value, and two-tailed unpaired t-test.
**C** Representative 3D images of young and aged human testis stained with α-SMA, FSP1 and Endoglin. Graph with the quantification of $FSP1^+$ fibroblast numbers per 1 $mm^2$ sample area. Data represent mean ± s.d. (n=4), P-value, and two-tailed unpaired t-test.
**D** Representative 3D images of young and aged human adrenal gland after α-SMA, FSP1 and CD31 immunostaining. The right graph shows the quantification of the number of $FSP1^+$ fibroblasts per 1 $mm^2$ sample area. Data represent mean ± s.d. (n=4), P-value, and two-tailed unpaired t-test.
**E** 3D images with α-SMA, FSP1 and CD31 in young and aged human thyroid gland. Graph with the quantification of $FSP1^+$ fibroblast numbers per 1 $mm^2$ sample area. Data represent mean ± s.d. (n=4), P-value, and two-tailed unpaired t-test.

Data information: Two-tailed Student's t-test was applied for the statistical analysis. *: $P < 0.05$; **: $P < 0.01$; ***: $P < 0.001$; ****: $P < 0.0001$. Scale bars 80 μm.

**Appendix Table S1**. Antibody list

| ANTIBODY | ALTERNATIVE NAME | CLONE | SPECIES | COMPANY | CAT NO. | WORK STATUS | TISSUE SPECIFICITY |
|---|---|---|---|---|---|---|---|
| **α-SMA** | ACTA1 | 1A4 | Mouse | Sigma-Aldrich | C6198 | YES | Myoepithelial cell; Artery EC |
| **α-SMA** | ACTA1 | 1A4 | Mouse | Sigma-Aldrich | A2547 | YES | Myoepithelial cell; Artery EC |
| **α-Tubulin** | TUBA1A | B-5-1-2 | Mouse | Thermo Fisher | 32-2500 | NO | NC |
| **BCAM** | MSK19; CD239 | Poly | Goat | R&D Systems | AF8299 | YES | EC |
| **Caveolin-1** | CAV1 | Poly | Rabbit | Cell signalling | 3238S | YES | EC |
| **Caveolin-1** | CAV1 | 2297 | Mouse | BD Biosciences | 610407 | NO | EC |
| **Caveolin-1** | CAV1 | Poly | Rabbit | Abcam | ab2910 | NO | EC |
| **CD102** | ICAM-2 | 3C4(mIC2/4) | Rat | BD Pharmingen | 553326 | YES | EC |
| **CD117** | c-kit | Poly | Goat | R&D Systems | AF1356 | YES | HSC; EC |
| **CD144** | CDH5; VE-cadherin | 55-7H1 | Mouse | BD Biosciences | 555661 | NO | EC |
| **CD144** | CDH5; VE-cadherin | Poly | goat | R&D Systems | AF1002 | NO | EC |
| **CD31** | PECAM-1; EndoCAM | 390 | Rat | BioLegend | 102401 | NO | EC |
| **CD31** | PECAM-1; EndoCAM | MEC13.3 | Rat | BD Biosciences | 553370 | YES | EC |
| **CD31** | PECAM-1; EndoCAM | Poly | Goat | R&D Systems | FAB3628G | YES | EC |
| **CD31** | PECAM-1; EndoCAM | MEC13.3 | Mouse | BD Pharmingen | 553371 | NO | EC |
| **CD34** | CD34 Molecule | RAM34 | Rat | eBioscience | 14-0341-81 | YES | HSC; EC |
| **CD34** | CD34 Molecule | EP373Y | Rabbit | Abcam | ab81289 | NO | HSC; EC |
| **CD34** | CD34 Molecule | EP373Y | Rabbit | Abcam | ab223930 | YES | HSC; EC |
| **CD45** | PTPRC; T200 | 30-F11 | Rat | BioLegend | 103101 | NO | HSC |
| **CD45** | PTPRC; T200 | 30-F11 | Rat | Thermo Fisher | 14-0451-81 | YES | HSC |
| **CD68** | SCARD1; GP110 | FA-11 | Rat | Abcam | ab53444 | YES | Macrophages |
| **CD90** | Thy1 | Poly | Sheep | R&D Systems | AF2067 | NO | HSC |
| **CD90** | Thy1 | EPR3133 | Rabbit | Abcam | ab133350 | YES | HSC |
| **Claudin-5** | CLDN5 | Poly | Rabbit | Abcam | ab15106 | YES | Tight junction |
| **Claudin-5** | CLDN5 | 4C3C2 | Mouse | Thermo Fisher | 35-2500 | NO | Tight junction |
| **Claudin-5** | CLDN5 | Poly | Rabbit | Thermo Fisher | 34-1600 | NO | Tight junction |
| **Collagen I** | COL1A1 | Poly | Rabbit | Merck Millipore | AB765P | YES | Mesenchymal Matrix |
| **Collagen IV** | Collagen Type IV | Poly | Rat | Bio-Rad | 2150-1470 | NO | Matrix; EC |
| **Collagen IV** | Collagen Type IV | Poly | Rabbit | Merck Millipore | AB756P | YES | Matrix; EC |
| **Collagen IV** | Collagen Type IV | 1042 | Mouse | Abcam | ab23975 | NO | Matrix; EC |
| **Collagen IV** | Collagen Type IV | C IV 22 | Mouse | Abcam | ab15633 | NO | Matrix; EC |
| **CXCl12** | SDF1; PBSF | D32F9 | Rabbit | Cell signalling | 3530 | YES | Pericyte; MC |
| **CXCR4** | LAP-3; LESTR | Poly | Goat | Abcam | ab1670 | YES | β-cell |
| **Decorin** | DCN; PG40 | Poly | Goat | R&D Systems | AF1060 | YES | Matrix |
| **Desmin** | DES; LGMD1D | Poly | Rabbit | Abcam | ab15200 | YES | MC |

| | | | | | | | |
|---|---|---|---|---|---|---|---|
| **Desmin** | DES; LGMD1D | D33 | Mouse | Dako | M0760 | NO | MC |
| **Dll4** | Deltalike protein 4 | Poly | Goat | R&D Systems | AF1389 | YES | EC; Pericyte; MC |
| **Dll4** | Deltalike protein 4 | Poly | Rabbit | Abcam | ab7280 | NO | EC; Pericyte; MC |
| **Dll4** | Deltalike protein 4 | 207822 | rat | R&D Systems | MAB1389 | NO | EC; Pericyte; MC |
| **DRAC** | ACKR1 | EPR5205 | Rabbit | Abcam | ab137044 | NO | EC |
| **EDAR** | ECTD10A | 132102 | Rat | R&D Systems | MAB745 | NO | Ectodysplasin A receptor |
| **eIF2$\alpha$** | EIF2S1 | D7D3 | Rabbit | Cell signalling | 5324T | YES | Multiple cell types |
| **Endoglin** | CD105 | Poly | Goat | R&D Systems | AF1097 | YES | EC |
| **Endoglin** | CD105 | Poly | Goat | R&D Systems | AF1320 | YES | EC |
| **Endoglycan** | PODXL2 | Poly | Goat | R&D Systems | AF3534 | YES | EC |
| **Endomucin** | Mucin-14 | V.7C7 | Rat | Santa Cruz | sc-65495 | YES | EC |
| **EPCR** | PROCR; CD201 | Poly | Goat | R&D Systems | AF2749 | YES | EC |
| **ERG** | Variant 10 | EPR3863 | Rabbit | Abcam | ab110639 | NO | EC |
| **ESM-1** | Endocan | poly | goat | R&D Systems | AF199 | YES | Tip cell |
| **ESAM** | LP4791 | 340236 | Rat | R&D Systems | MAB28271 | NO | EC |
| **F4/80** | EMR1 | CI:A3-1 | Rat | Bio-Rad | MCA497GA | YES | Macrophages |
| **FABP4** | A-FABP | Poly | Goat | R&D Systems | AF1443 | YES | Adipocytes |
| **Fibronectin** | FN1 | Poly | Rabbit | abcam | ab2413 | YES | Matrix |
| **Fibronectin** | FN1 | 10/Fibronectin | Mouse | BD Biosciences | 610078 | NO | Matrix |
| **GJA1** | GJAL; Connexin-43 | Poly | Rabbit | Sigma-Aldrich | SAB4300504 | YES | Gap junctions |
| **GJA5** | Connexin-40 | Poly | Rabbit | Alpha Diagnostics | CX40-A | NO | Gap junctions |
| **GJA8** | Connexin-50 | c6 | mouse | Thermo Fisher | 33-4300 | NO | Gap junctions |
| **HIF1$\alpha$** | Anti-Hypoxia Inducible Factor 1 | H1alpha67 | Mouse | EMD Millipore | MAB5382 | NO | Multiple cell types |
| **HIF1$\alpha$** | Anti-Hypoxia Inducible Factor 1 | Poly | rabbit | Abcam | ab2185 | YES | Multiple cell types |
| **HSPG2** | PLC | A7L6 | Rat | Merck Millipore | MAB1948P | YES | Matrix |
| **Ki67** | MKI67 | Poly | Rabbit | Abcam | ab15580 | YES | Mitotic chromosome |
| **ICAM-1** | CD54 | 11C81 | Mouse | R&D Systems | BBA3 | NO | EC |
| **ICAM-1** | CD54 | Poly | Goat | R&D Systems | AF796 | YES | EC |
| **ICAM-1** | CD54 | EP1442Y | Rabbit | Abcam | ab53013 | YES | EC |
| **Insulin** | INS | Poly | Rabbit | Abcam | ab63820 | YES | $\beta$-cell |
| **Laminin** | LAMA1 | Poly | Rabbit | Sigma-Aldrich | L9393 | YES | Matrix |
| **Nestin** | NES | rat 401 | Mouse | Sigma-Aldrich | MAB353 | NO | NC |
| **NG2** | CSPG4 | Poly | Rabbit | Merck Millipore | AB5320 | YES | Pericytes |
| **P-Histone H3** | | Poly | Rabbit | Merck Millipore | 06-570 | YES | Mitotic chromosome |
| **P-selectin** | CD62P | Poly | Goat | R&D Systems | AF737 | YES | EC |
| **PDGFR$\alpha$** | CD140a; PDGFRA | Poly | Goat | R&D Systems | AF1062 | YES | MC |
| **PDGFR$\alpha$** | CD140a; PDGFRA | Poly | Rabbit | Sigma-Aldrich | SAB4502142 | NO | MC |

| | | | | | | | |
|---|---|---|---|---|---|---|---|
| **PDGFR$\alpha$** | CD140a; PDGFRA | APA5 | rat | Thermo Fisher | 14-1401-82 | NO | MC |
| **PDGFR$\beta$** | CD140b; PDGFRB | Y92 | Rabbit | Abcam | ab32570 | YES | Pericytes |
| **PDGFR$\beta$** | CD140b; PDGFRB | 28/CD140b | mouse | BD Biosciences | 610113 | NO | Pericytes |
| **PDGFR$\beta$** | CD140b; PDGFRB | Poly | rabbit | Merck Millipore | 06-495 | NO | Pericytes |
| **PDGFR$\beta$** | CD140b; PDGFRB | Poly | goat | R&D Systems | AF1042 | NO | Pericytes |
| **Perilipin** | PLIN | D1D8 | Rabbit | Cell signalling | 9349 | YES | Adipocytes |
| **PLVAP** | FELS; PV-1 | MECA-32 | Rat | Abcam | ab27853 | NO | EC |
| **PLVAP** | FELS; PV-1 | MECA-32 | Rat | Bio-Rad | MCA2539 | YES | EC |
| **Podocalyxin** | PODXL; PCLP-1 | Poly | Goat | R&D Systems | AF1556 | YES | EC |
| **Podocalyxin** | PODXL; PCLP-1 | 222328 | Mouse | R&D Systems | MAB1658 | NO | EC |
| **Podoplanin** | PDPN; GP36 | Poly | Goat | R&D Systems | AF3244 | YES | EC |
| **PROX1** | Prospero Homeobox 1 | Poly | Rabbit | Abcam | ab15580 | NO | Lymphatic cell |
| **Sca-1** | Ly-6A/E | Poly | Goat | R&D Systems | AF1226 | YES | HSC |
| **SM22$\alpha$** | TAGLN | Poly | Rabbit | Abcam | ab14106 | YES | Vascular SMC |
| **Smoothelin** | SMTN | EPR20044 | Rabbit | Abcam | ab219652 | YES | SMC |
| **Tie-2** | TEK | Poly | Goat | R&D Systems | AF762 | YES | EC |
| **Tie-2** | TEK | Cl. 16 | Mouse | Abcam | ab24859 | YES | EC |
| **VCAM-1** | CD106 | Poly | Goat | R&D Systems | AF643 | YES | EC |
| **VEGFA** | Vascular Endothelial Growth Factor A | Poly | Rabbit | Abcam | ab52917 | YES | Growth factor |
| **VEGFR2** | KDR; FLK1 | Poly | Rabbit | Abcam | ab39256 | YES | EC |
| **VEGFR3** | Flt-4 | Poly | Goat | R&D Systems | AF743 | YES | Lymphatic EC |
| **VEGFR3** | Flt-4 | 54703 | Mouse | R&D Systems | MAB3491 | NO | Lymphatic EC |
| **Vimentin** | VIM | V9 | Mouse | Dako | M0725 | NO | Matrix |
| **Vimentin** | VIM | EPR3776 | Rabbit | Abcam | ab92547 | YES | Matrix |
| **Vinculin** | VCL | EPR20407 | Rabbit | Abcam | ab219649 | YES | Matrix |
| **Vinculin** | VCL | hVIN-1 | mouse | Sigma-Aldrich | V9131 | NO | Matrix |
| **VWF** | F8VWF | F8/86 | Mouse | Dako | M0616 | NO | Adipocytes |
| **p27** | Kip1; CDKN1B | Poly | Rabbit | Thermo Fisher | PA5-16717 | YES | CDK inhibitor |
| **p21** | Cip1; CDKN1A | Poly | Rabbit | Thermo Fisher | PA1-30399 | YES | CDK inhibitor |

EC: endothelial cell; NC: nerve cell; HSC: hematopoietic stem cell; MC: mesenchymal cell; SMC: smooth muscle cell

**Appendix Table S2**. Single-cell 3D mapping image database of young versus aged murine adrenal glands

| Antibodies | Young | Aged | Database No. |
|---|---|---|---|
| NG2 (488)<br>Podocalyxin (546)<br>HSPG2 (594)<br>TO-PRO-3 (647) | 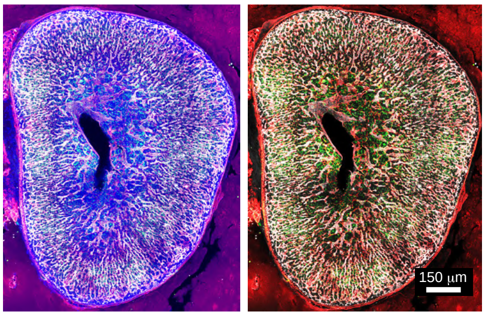  | 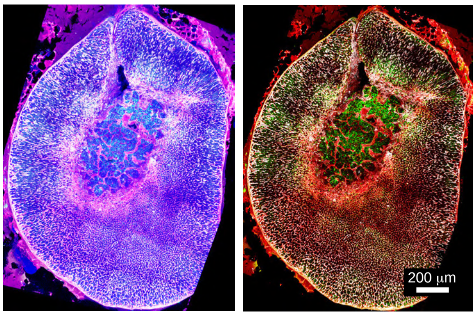  | 1 |
| Vinculin (488)<br>Dll4 (546)<br>Emcn (594)<br>TO-PRO-3 (647) | 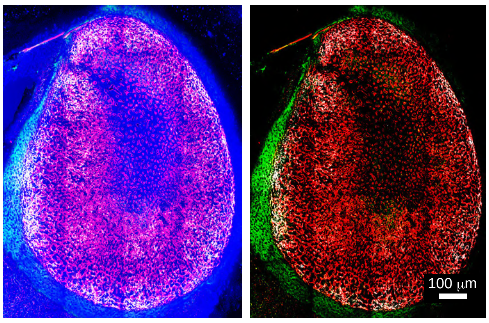  | 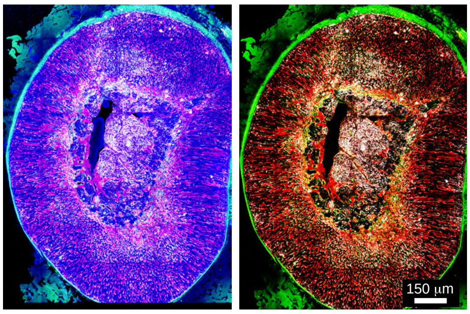  | 2 |
| α-SMA (488)<br>Emcn (594)<br>TO-PRO-3 (647) | 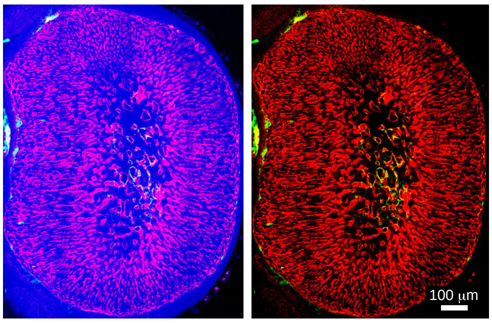  | 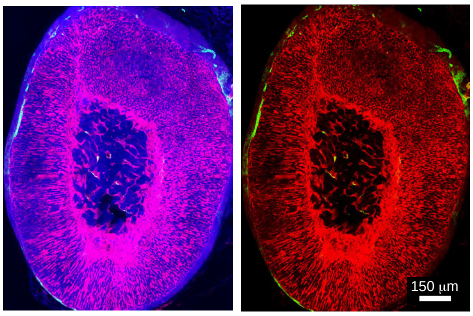  | 3 |
| NG2 (488)<br>Sca-1 (546)<br>Emcn (594)<br>TO-PRO-3 (647) | 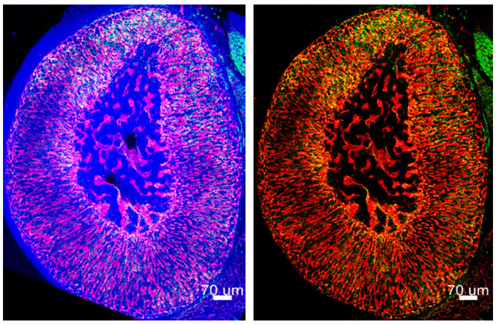  | 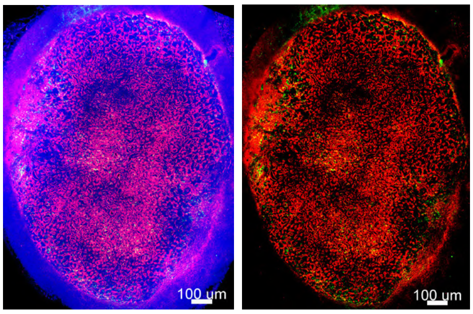  | 4 |
| Vimentin (488)<br>CXCR4 (546)<br>Emcn (594)<br>TO-PRO-3 (647) | 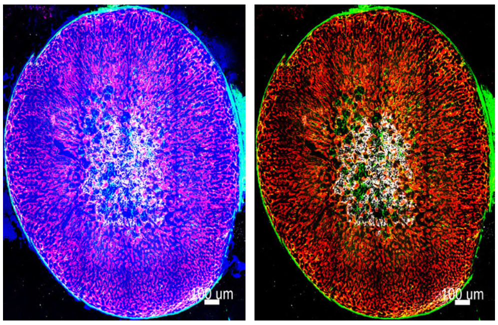  | 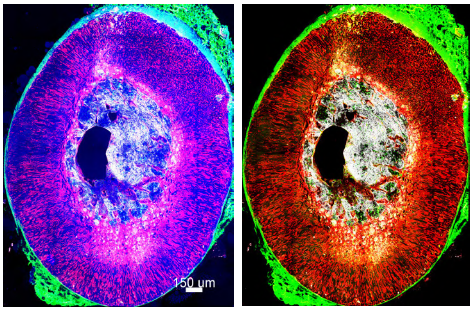  | 5 |

| Antibodies | Young | Aged | Database No. |
|---|---|---|---|
| Collagen IV (488)<br>Endoglin (546)<br>F4/80 (594)<br>TO-PRO-3 (647) | 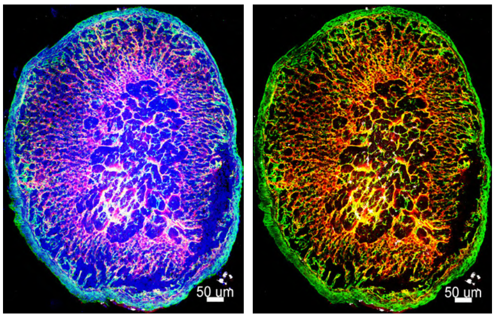 | 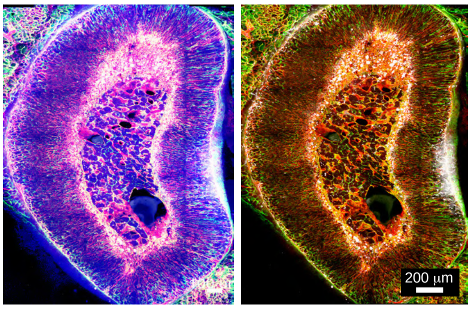 | 6 |
| VCAM-1 (546)<br>CD31 (594)<br>TO-PRO-3 (647) | 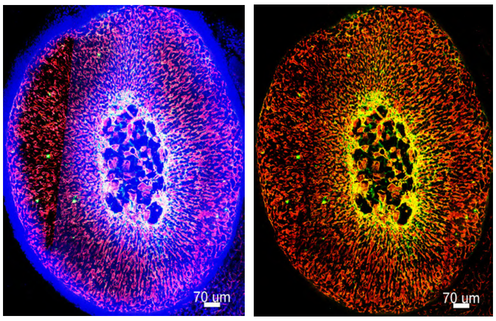 | 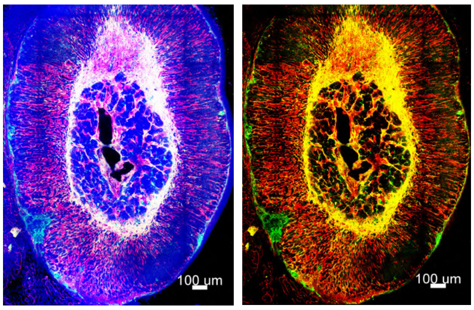 | 7 |
| PDGFRβ (488)<br>Emcn (546)<br>TO-PRO-3 (647) | 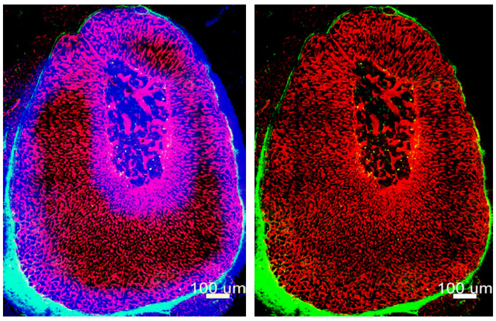 | 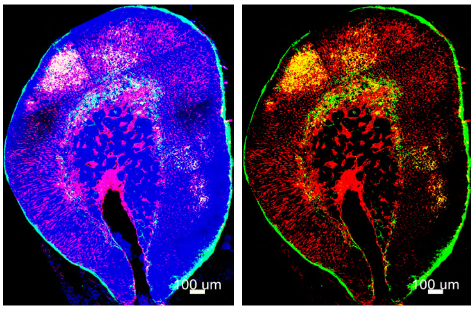 | 8 |
| Fibronectin (488)<br>Endoglin (546)<br>CD68 (594)<br>TO-PRO-3 (647) | 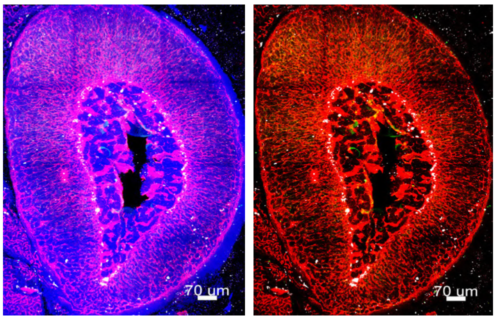 | 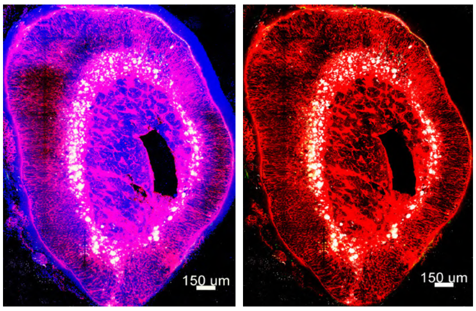 | 9 |
| FSP1 (488)<br>Isolectin (546)<br>Emcn (594)<br>Decorin (647)<br>DAPI (405) | 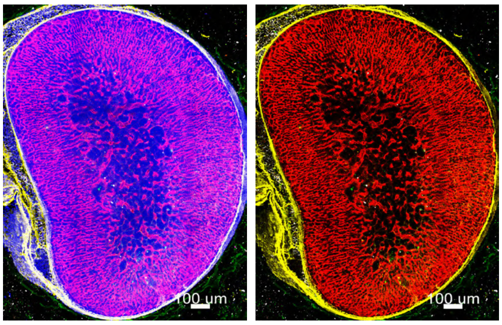 | 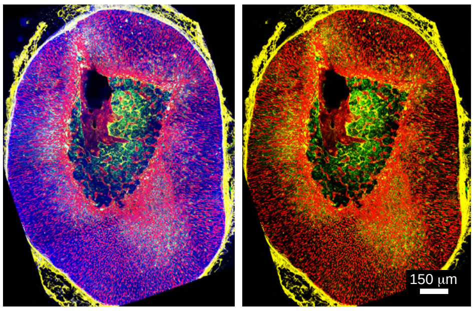 | 10 |

| Antibodies | Young | Aged | Database No. |
|---|---|---|---|
| Perilipin (488)<br>Tie-2 (546)<br>Emcn (594)<br>TO-PRO-3 (647) | 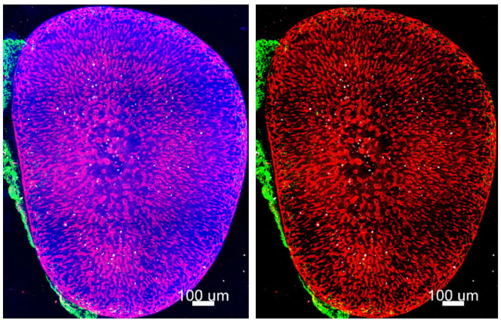  | 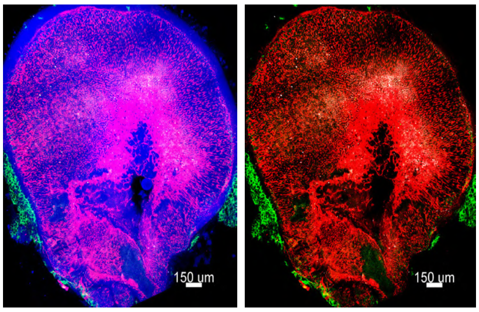  | 11 |
| Collagen I (488)<br>P-selectin (546)<br>CD102 (594)<br>TO-PRO-3 (647) | 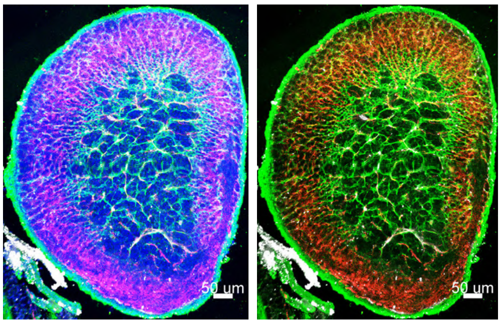  | 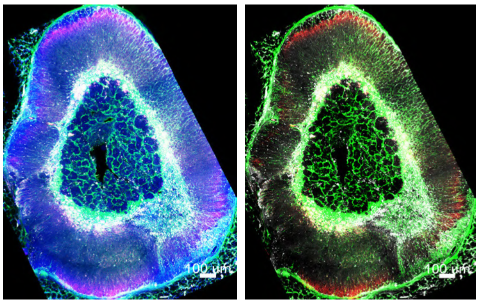  | 12 |
| HIF1α (488)<br>Endoglin (546)<br>HSPG2 (594)<br>TO-PRO-3 (647) | 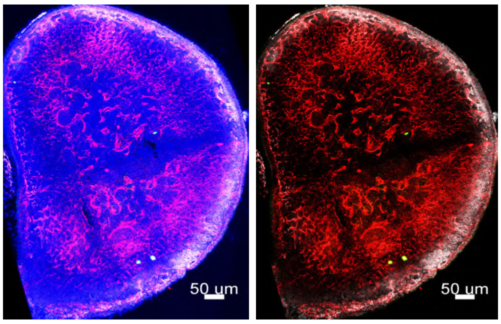  | 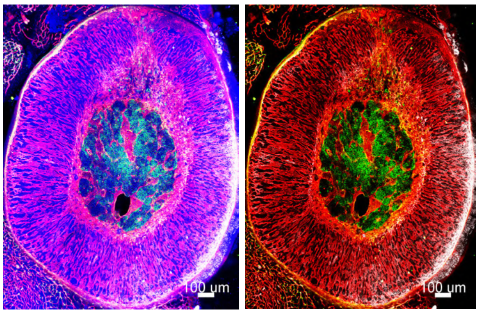  | 13 |
| Laminin (488)<br>VCAM-1 (546)<br>TO-PRO-3 (647) | 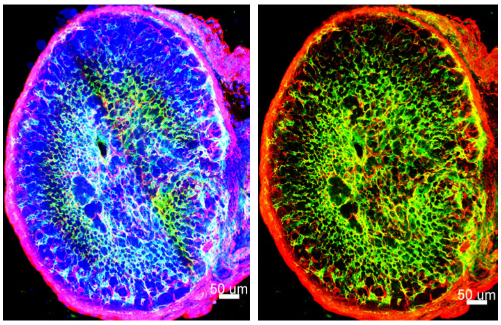  | 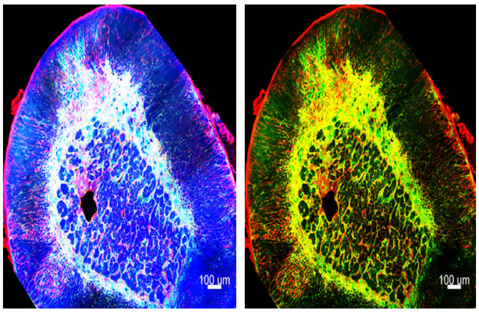  | 14 |
| α-SMA (546)<br>Emcn (594)<br>TO-PRO-3 (647) | 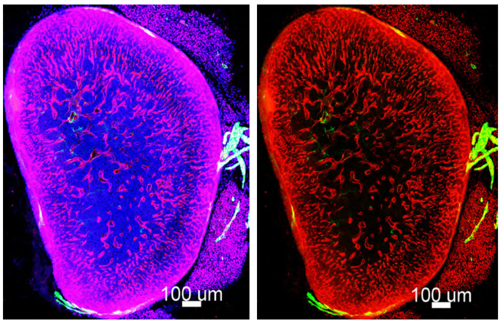  | 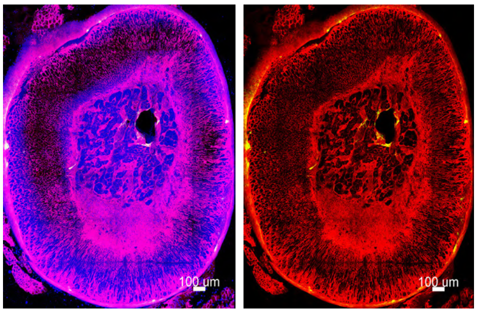  | 15 |

| Antibodies | Young | Aged | Database No. |
|---|---|---|---|
| VEGFA (488)<br>Emcn (594)<br>TO-PRO-3 (647) | 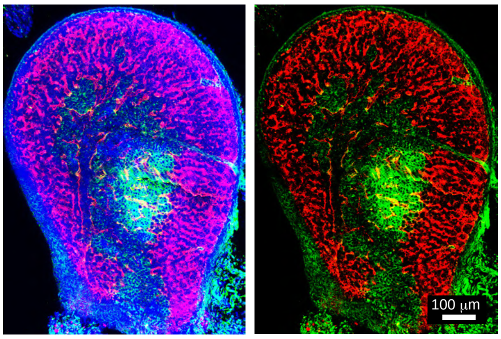 | 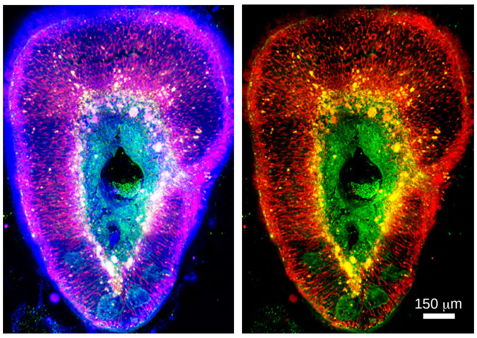 | 184, 185 |

**Appendix Table S3**. Single-cell 3D mapping image database of murine adrenal glands with various niche markers

| Antibodies | Young | Antibodies | Young |
|---|---|---|---|
| SM22α (488)<br>Emcn (594)<br>TO-PRO-3 (647) | 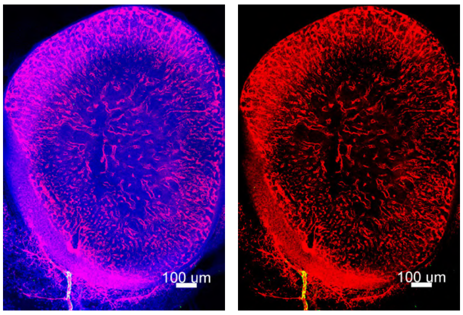  | HIF1α (488)<br>Decorin (546)<br>Emcn (594)<br>TO-PRO-3 (647) | 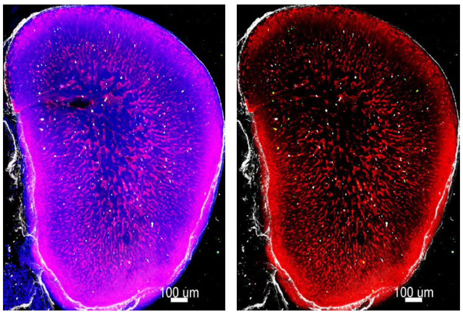  |
| PDGFRα (488)<br>Emcn (594)<br>TO-PRO-3 (647) | 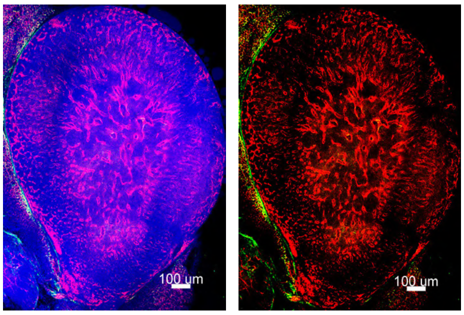  | EPCR (488)<br>α-SMA (546)<br>HSPG2 (594)<br>TO-PRO-3 (647) | 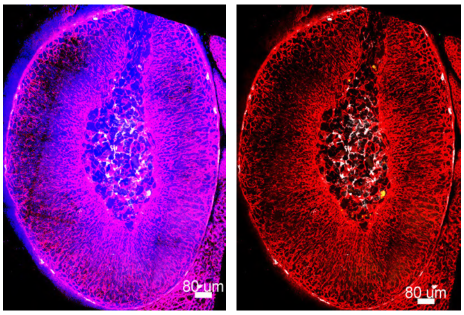  |
| Perilipin (488)<br>FABP4 (546)<br>Emcn (594)<br>TO-PRO-3 (647) | 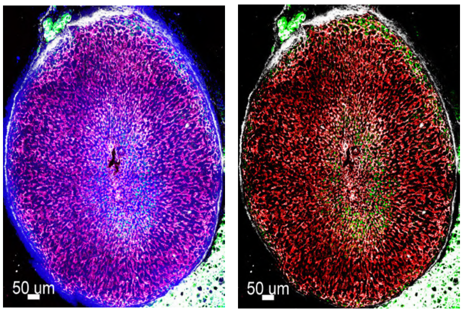  | Laminin (488)<br>BCAM (546)<br>TO-PRO-3 (647) | 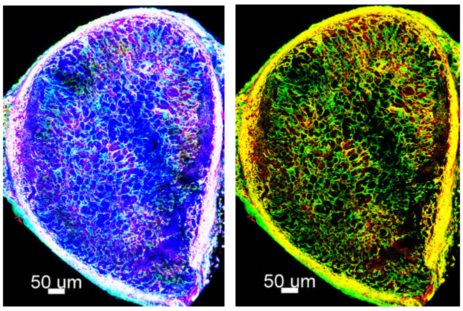  |
| FSP1 (546)<br>Emcn (594)<br>TO-PRO-3 (647) | 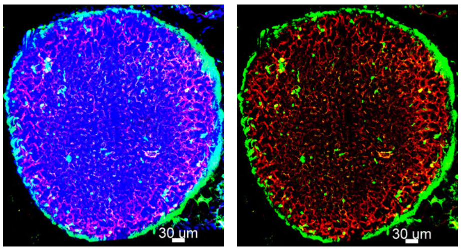  | FSP1 (546)<br>Emcn (594)<br>TO-PRO-3 (647) | 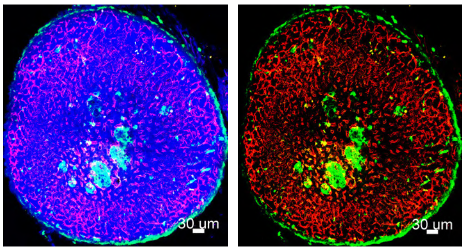  |
| VEGFR3 (546)<br>Emcn (594)<br>TO-PRO-3 (647) | 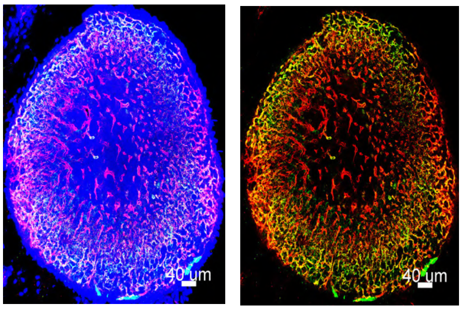  | VEGFR3 (546)<br>Emcn (594)<br>TO-PRO-3 (647) | 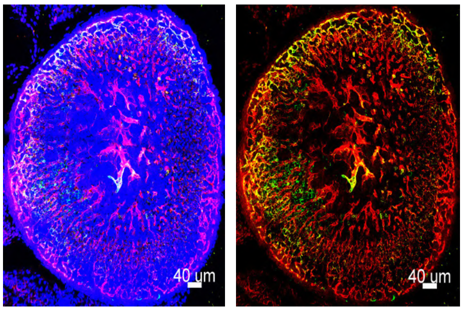  |
| Desmin (546)<br>Emcn (594)<br>TO-PRO-3 (647) | 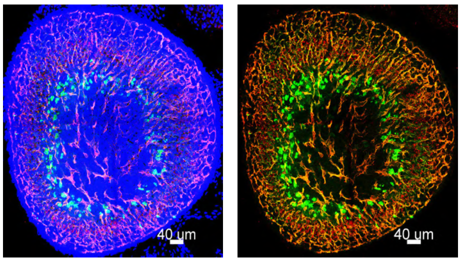  | | |

| Antibodies | Aged | Antibodies | Aged |
|---|---|---|---|
| PDGFRβ (488)<br>Emcn (594) | 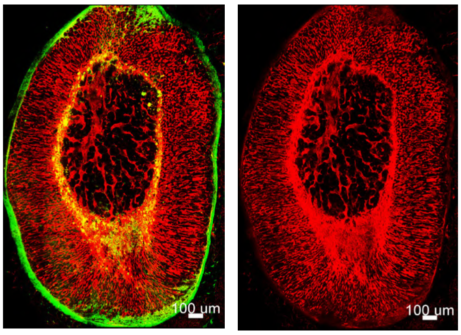 | VCAM-1 (546)<br>Emcn (594)<br>TO-PRO-3 (647) | 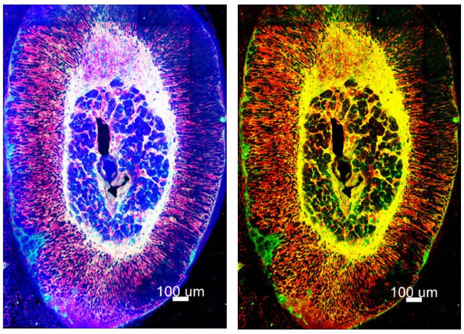 |
| α-SMA (546)<br>Emcn (594)<br>TO-PRO-3 (647) | 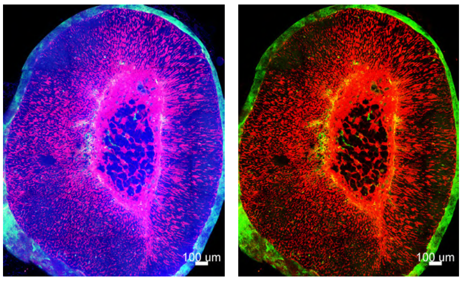 | | |

**Appendix Table S4**. Single-cell 3D mapping image database of young versus aged murine ovaries

| Antibodies | Young | Aged | Database No. |
|---|---|---|---|
| Vinculin (488)<br>Dll4 (546)<br>Emcn (594)<br>TO-PRO-3 (647) | 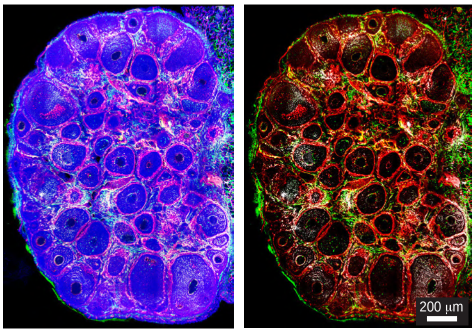 | 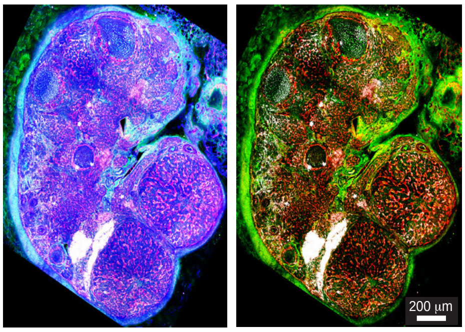 | 1 |
| PDGFRβ (488)<br>Sca-1 (546)<br>Emcn (594)<br>TO-PRO-3 (647) | 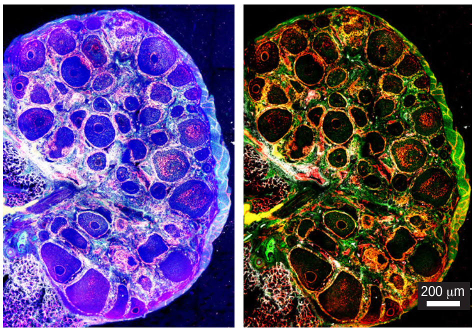 | 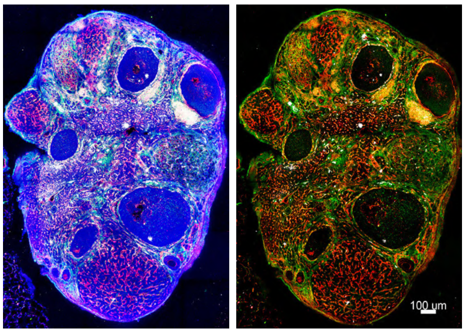 | 2 |
| Claudin-5 (488)<br>Endocan (546)<br>Emcn (594)<br>TO-PRO-3 (647) | 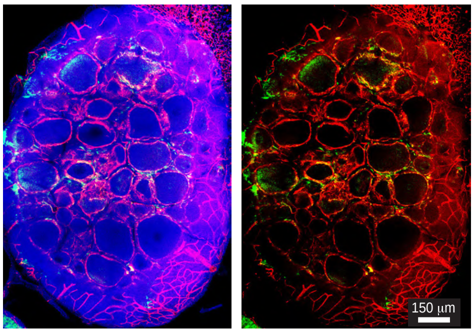 | 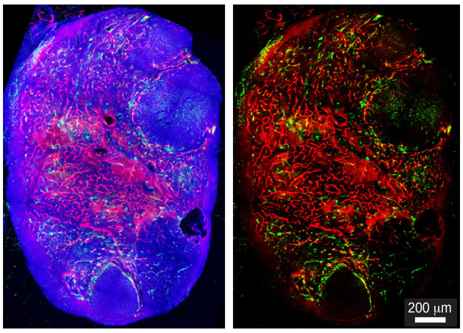 | 3 |
| GJA-1(488)<br>Endoglin (546)<br>CD102 (594)<br>TO-PRO-3 (647) | 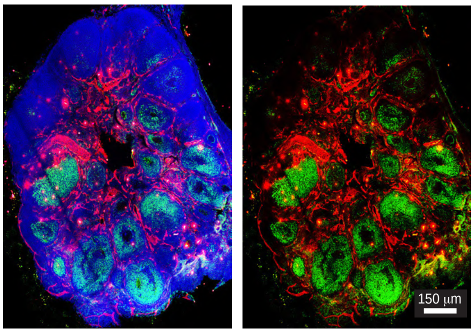 | 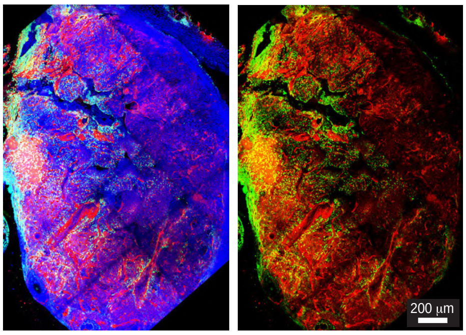 | 4 |
| Smoothelin (488)<br>Endoglin (546)<br>TO-PRO-3 (647) | 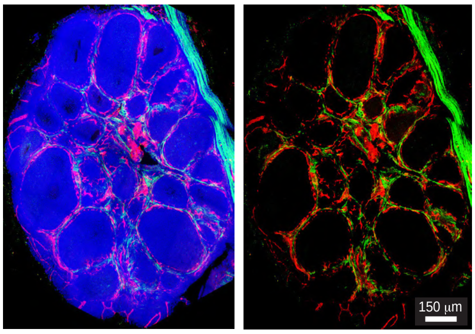 | 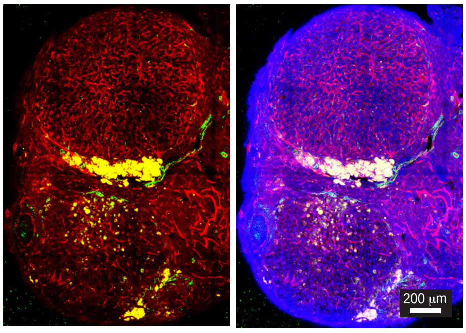 | 5 |

| Antibodies | Young | Aged | Database No. |
|---|---|---|---|
| Perilipin (488)<br>Tie-2 (546)<br>Emcn (594)<br>TO-PRO-3 (647) | 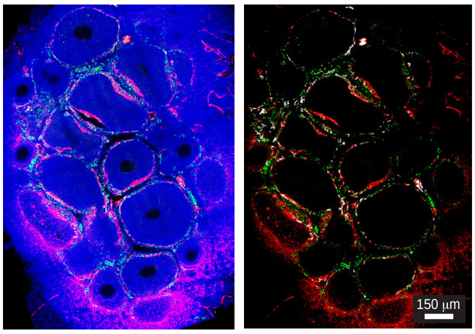 | 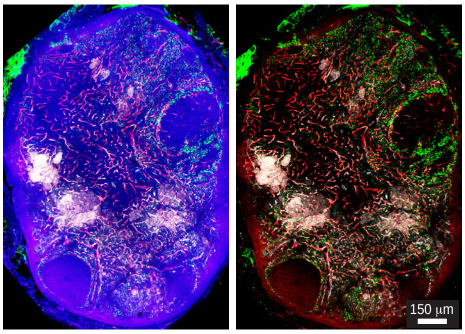 | 6 |
| Vimentin(488)<br>Endoglin (546)<br>TO-PRO-3 (647) | 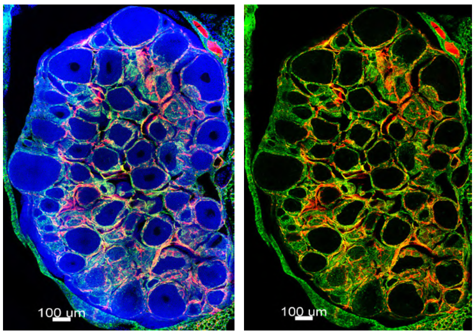 | 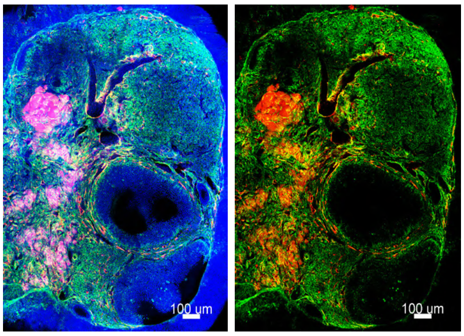 | 7 |
| Fibronectin (488)<br>Endoglin (546)<br>CD68 (594)<br>TO-PRO-3 (647) | 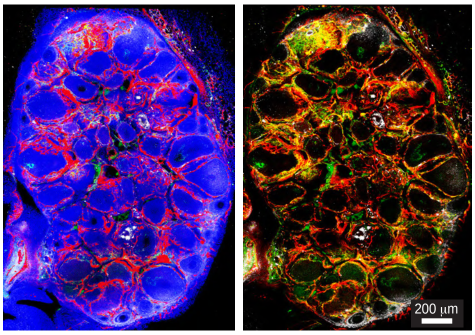 | 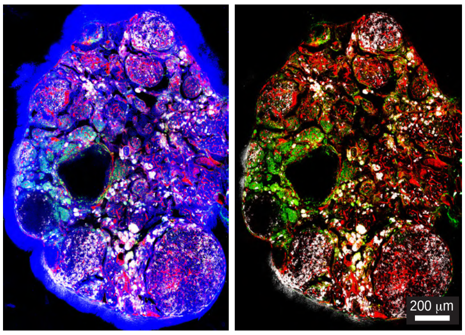 | 8 |
| Laminin (488)<br>Podocalyxin (546)<br>TO-PRO-3 (647) | 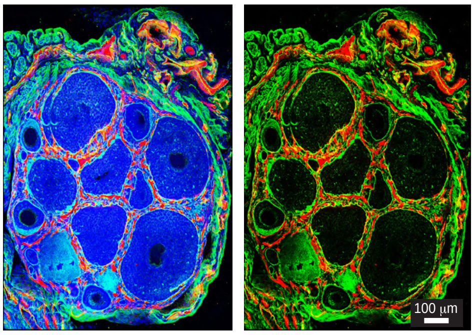 | 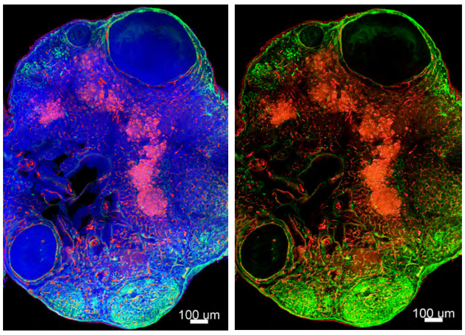 | 9 |
| PDGFRα (488)<br>Endoglin (546)<br>TO-PRO-3 (647) | 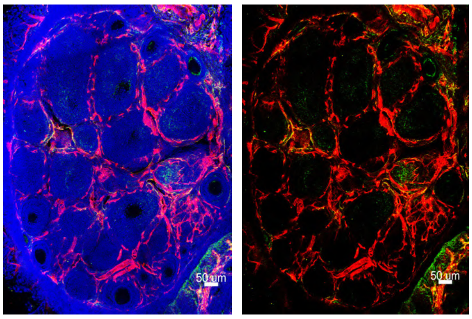 | 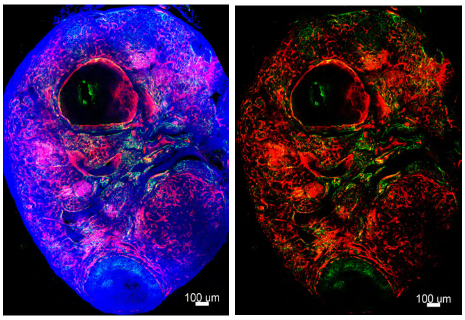 | 10 |

| Antibodies | Young | Aged | Database No. |
|---|---|---|---|
| Vimentin (488)<br>α-SMA (546)<br>HSPG2 (594)<br>TO-PRO-3 (647) | 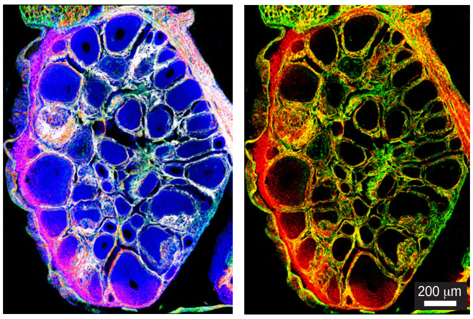 | 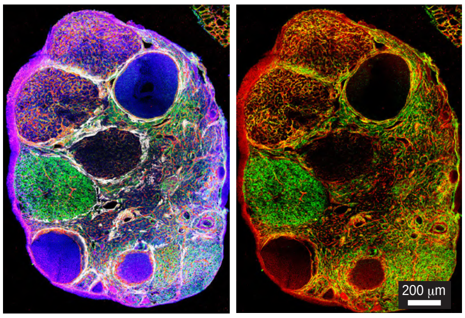 | 11 |
| PDGFRβ (488)<br>c-kit(546)<br>HSPG2 (594)<br>TO-PRO-3 (647) | 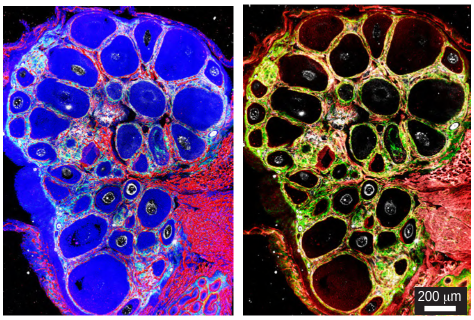 | 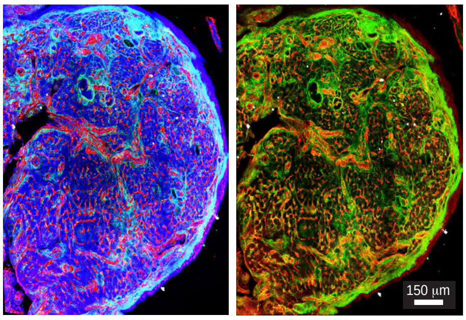 | 12 |
| FSP1 (488)<br>Isolectin (546)<br>CD102 (594)<br>Endoglin (647)<br>DAPI (405) | 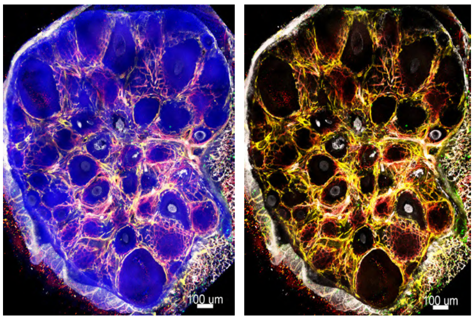 | 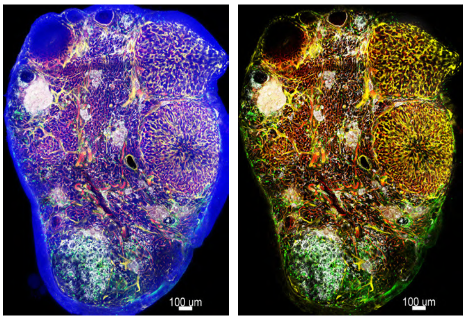 | 13 |
| GJA-1 (488)<br>VEGFR3 (546)<br>Emcn (594)<br>TO-PRO-3 (647) | 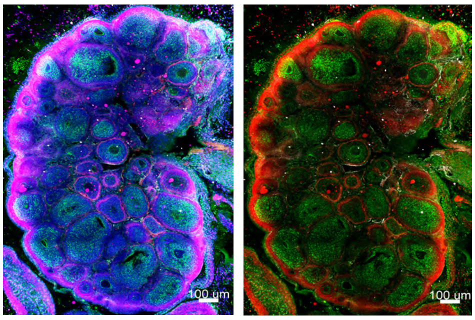 | 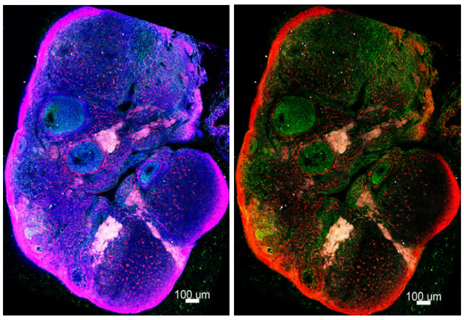 | 14 |
| FSP1 (488)<br>Isolectin (546)<br>Emcn (594)<br>Decorin (647)<br>DAPI (405) | 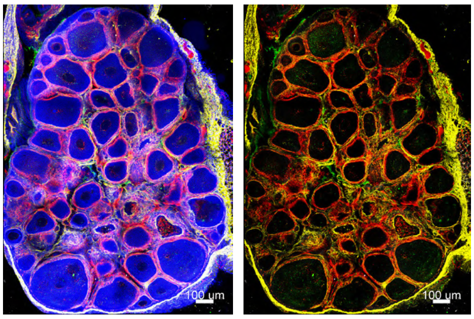 | 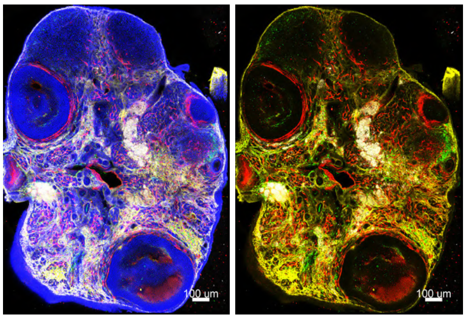 | 15 |

| Antibodies | Young | Aged | Database No. |
|---|---|---|---|
| FSP1 (488)<br>Isolectin (546)<br>CD34 (594)<br>Endoglin (647)<br>DAPI (405) | 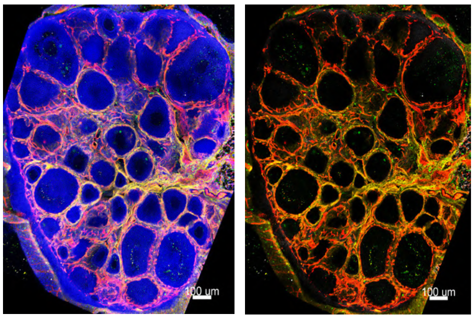 | 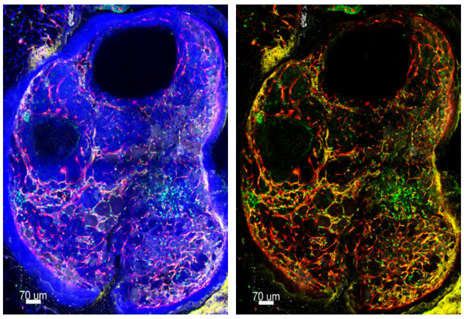 | 16 |
| SM22α (488)<br>HSPG2 (594)<br>TO-PRO-3 (647) | 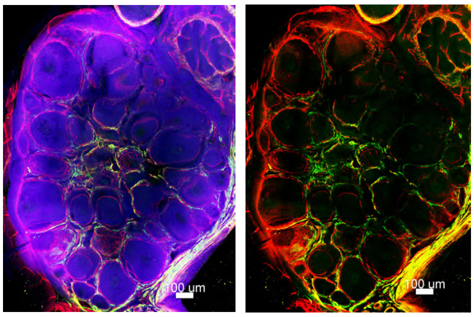 | 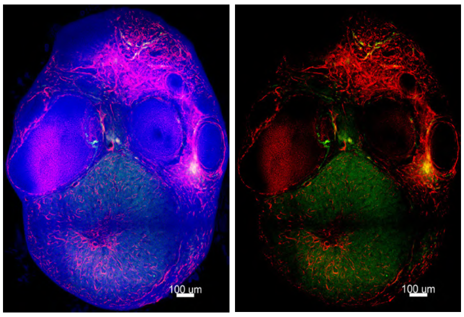 | 17 |
| VEGFR3 (546)<br>Emcn (594)<br>TO-PRO-3 (647) | 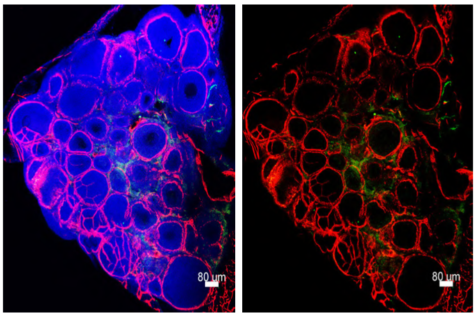 | 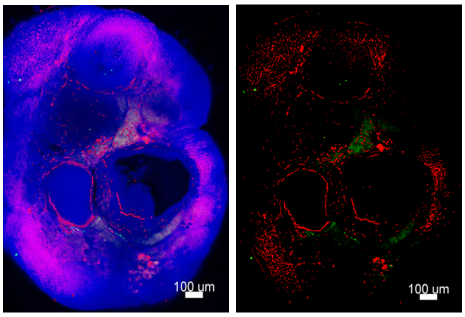 | 18 |
| Laminin (488)<br>Podocalyxin (546)<br>TO-PRO-3 (647) | 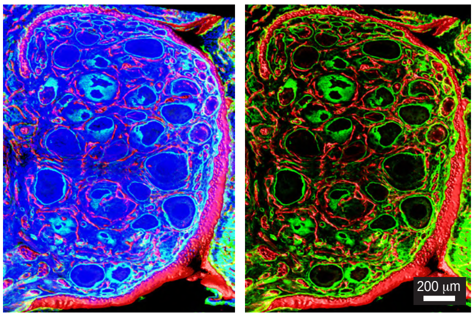 | 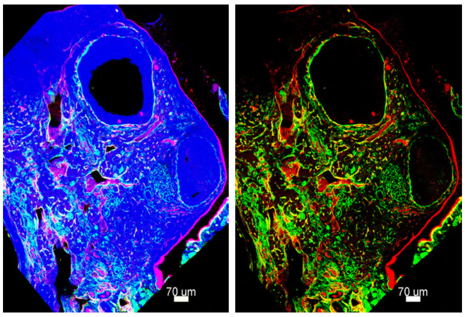 | 19 |
| Laminin (488)<br>Endoglin (546)<br>TO-PRO-3 (647) | 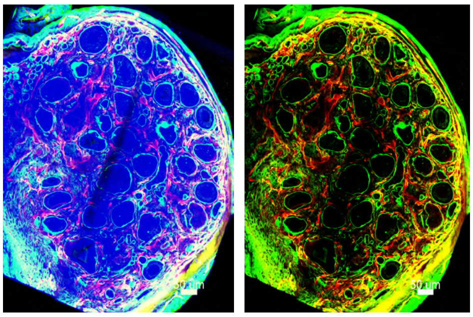 | 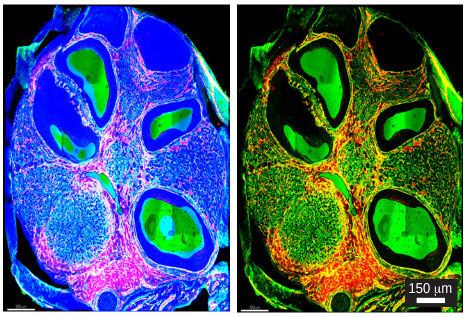 | 20 |

| Antibodies | Young | Aged | Database No. |
|---|---|---|---|
| HIF1α (488)<br>Endoglin (546)<br>HSPG2 (594)<br>TO-PRO-3 (647) | 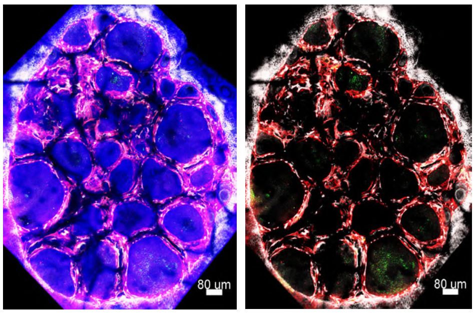 | 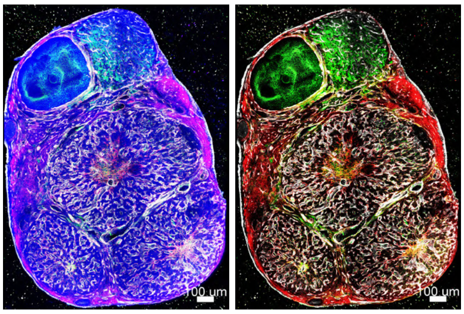 | 21 |
| α-SMA (546)<br>HSPG2 (594)<br>TO-PRO-3 (647) | 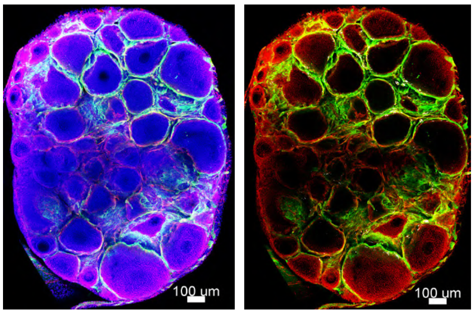 | 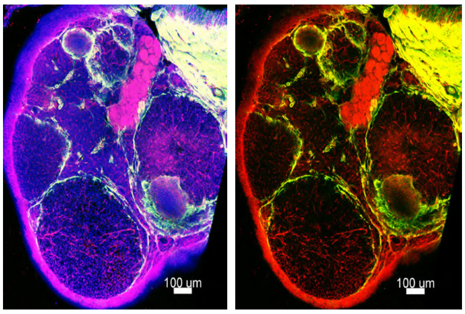 | 22 |
| Smoothelin (488)<br>BCAM (546)<br>HSPG2 (594)<br>TO-PRO-3 (647) | 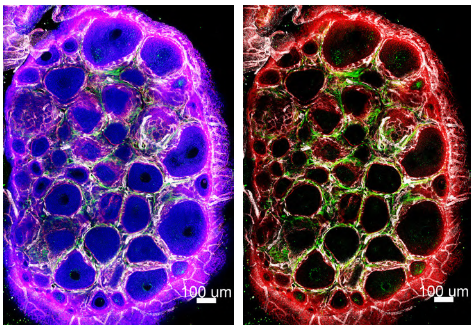 | 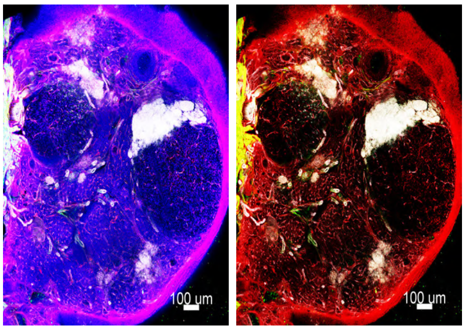 | 23 |
| Collagen IV (488)<br>α-SMA (546)<br>HSPG2 (594)<br>TO-PRO-3 (647) | 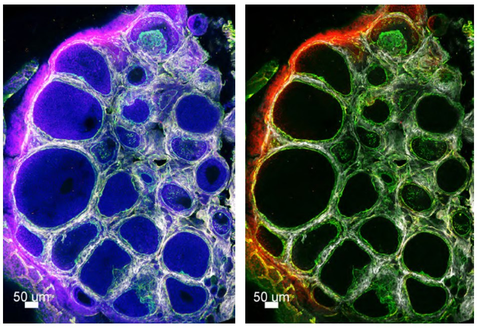 | 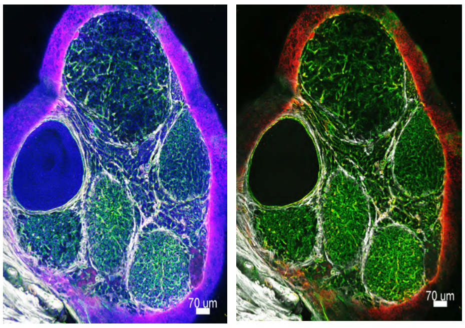 | 24 |
| HIF1α (488)<br>Decorin (546)<br>Emcn (594)<br>TO-PRO-3 (647) | 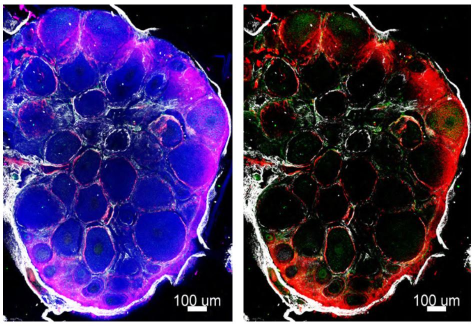 | 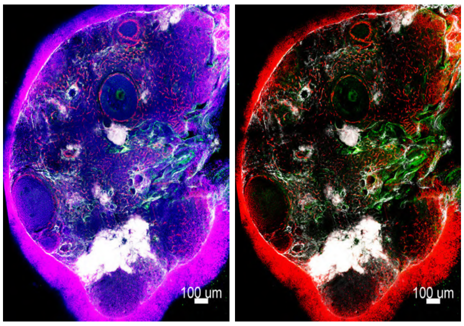 | 25 |

| Antibodies | Young | Aged | Database No. |
|---|---|---|---|
| VEGFA (488)<br>Emcn (594)<br>TO-PRO-3 (647) | 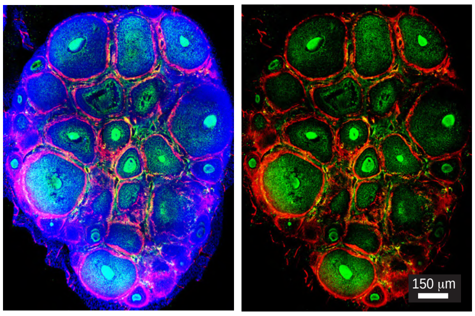 | 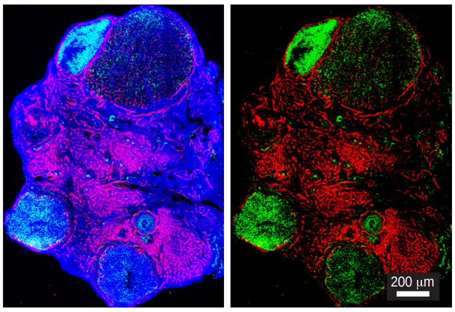 | 146, 147 |

**Appendix Table S5**. Single-cell 3D mapping image database of murine ovaries with various niche markers

| Antibodies | Young | Antibodies | Young |
|---|---|---|---|
| Smoothelin (488)<br>Emcn (594)<br>TO-PRO-3 (647) | 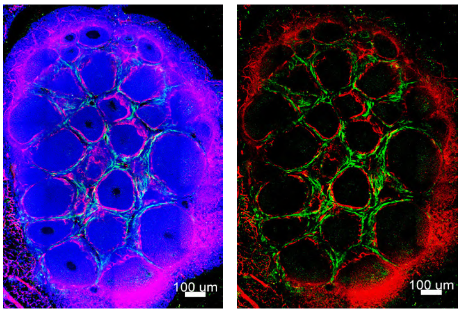 | CD31 (488)<br>Emcn (594)<br>TO-PRO-3 (647) | 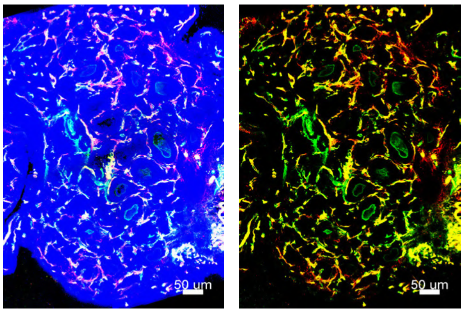 |
| Desmin (488)<br>CD31 (546)<br>TO-PRO-3 (647) | 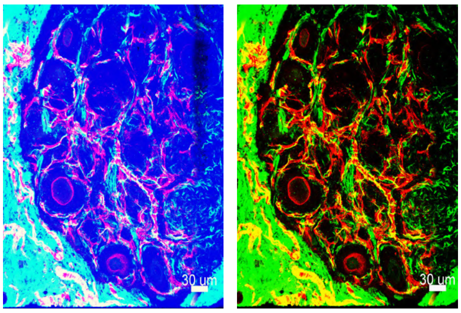 | CD34 (488)<br>CD31 (546)<br>TO-PRO-3 (647) | 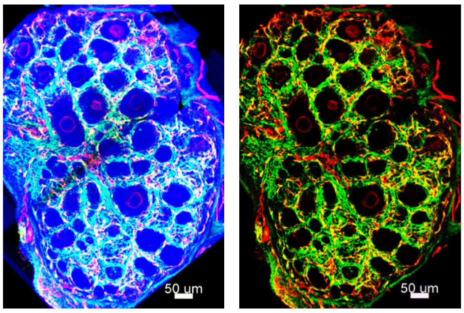 |
| Desmin (488)<br>CD31 (546)<br>TO-PRO-3 (647) | 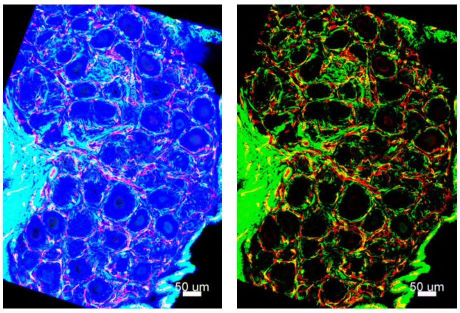 | Laminin (488)<br>P-selectin (546)<br>TO-PRO-3 (647) | 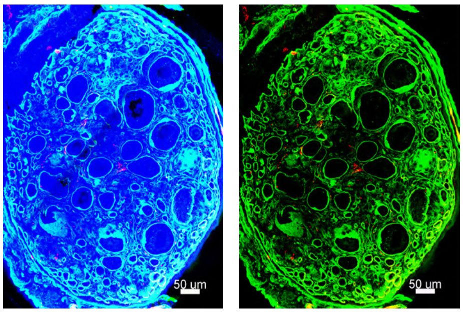 |
| Laminin (488)<br>BCAM (546)<br>TO-PRO-3 (647) | 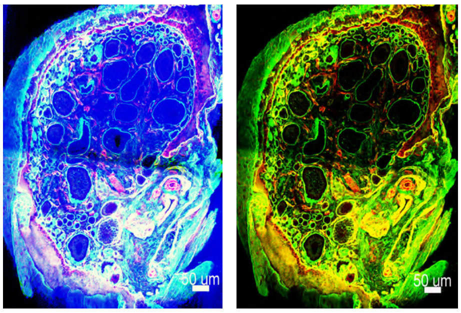 | Laminin (488)<br>VEGFR3 (546)<br>TO-PRO-3 (647) | 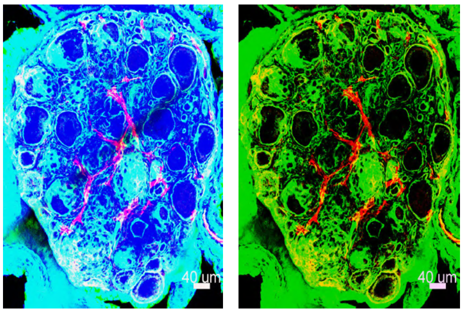 |
| Laminin (488)<br>FABP4 (546)<br>TO-PRO-3 (647) | 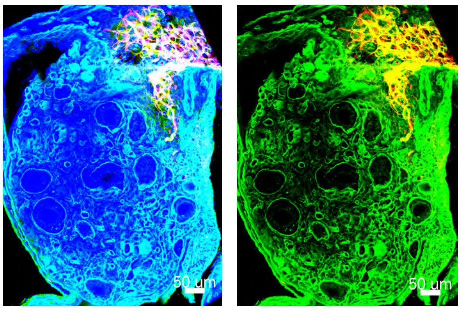 | | |

| Antibodies | Aged | Antibodies | Aged |
| --- | --- | --- | --- |
| GJA-1 (488)<br>Endoglin (546)<br>CD102 (594)<br>TO-PRO-3 (647) | 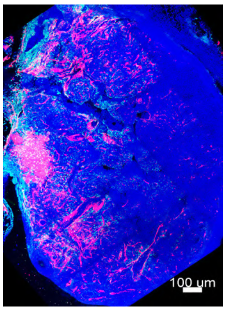  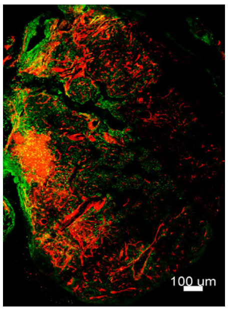  | | |

**Appendix Table S6**. Single-cell 3D mapping image database of young versus aged murine pituitary glands

| Antibodies | Young | Aged | Database No. |
|---|---|---|---|
| Vimentin (488)<br>Dll4 (546)<br>Emcn (594)<br>TO-PRO-3 (647) | 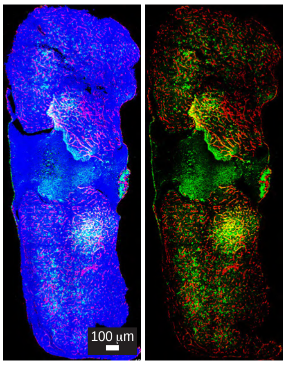 | 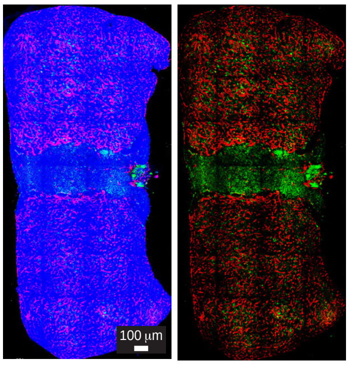 | 1 |
| Claudin-5 (488)<br>EPCR (546)<br>Emcn (594)<br>TO-PRO-3 (647) | 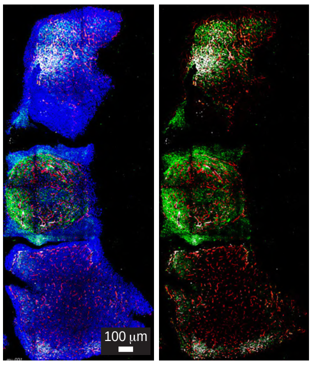 | 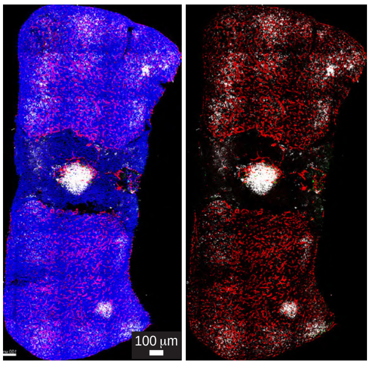 | 2 |
| Laminin (488)<br>c-kit (546)<br>Emcn (594)<br>TO-PRO-3 (647) | 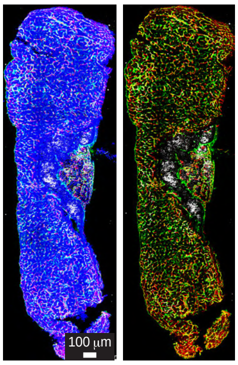 | 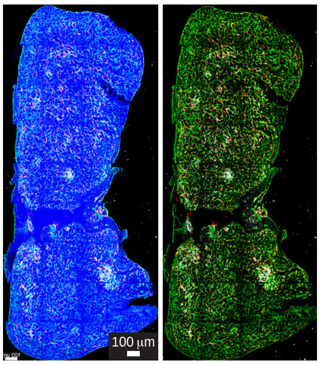 | 3 |
| Desmin (488)<br>VCAM-1 (546)<br>Emcn (594)<br>TO-PRO-3 (647) | 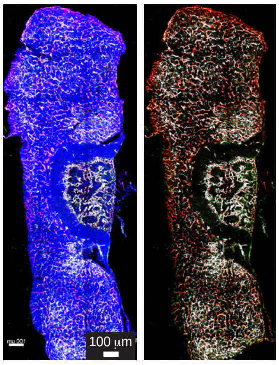 | 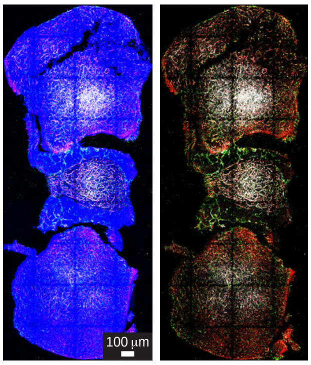 | 4 |
| SM22α (488)<br>Isolectin (546)<br>Emcn (594)<br>PDGFRα (647)<br>DAPI (405) | 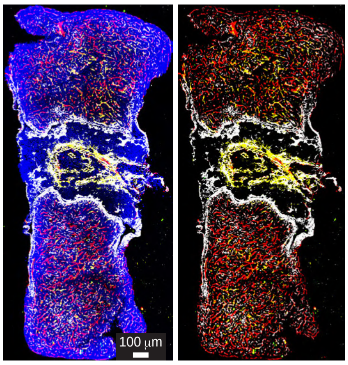 | 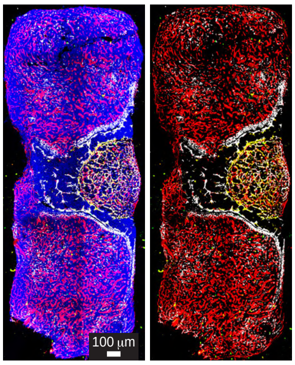 | 5 |

| Antibodies | Young | Aged | Database No. |
|---|---|---|---|
| Fibronectin (488)<br>Endoglycan (546)<br>Emcn (594)<br>TO-PRO-3 (647) | 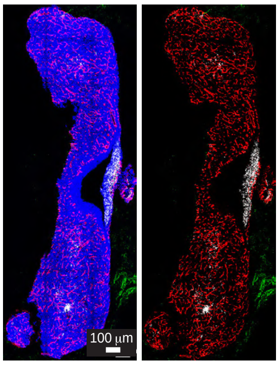 | 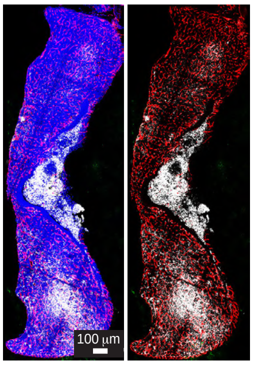 | 6 |
| Perilipin (488)<br>Sca-1 (546)<br>Emcn (594)<br>TO-PRO-3 (647) | 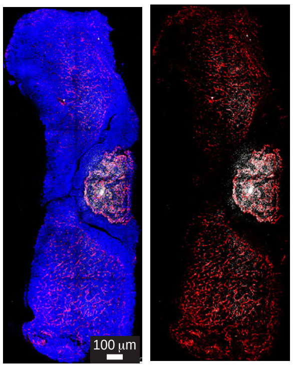 | 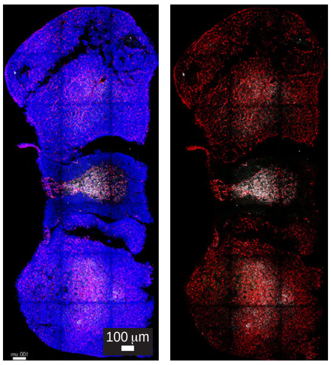 | 7 |
| NG2 (488)<br>FABP4 (546)<br>HSPG2 (594)<br>TO-PRO-3 (647) | 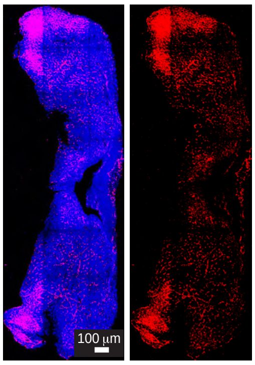 | 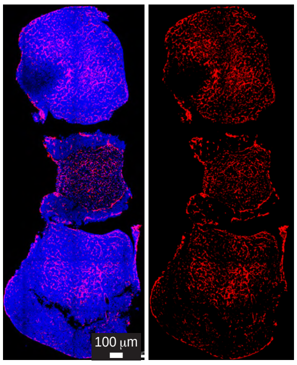 | 8 |
| Collagen IV (488)<br>ESM-1 (546)<br>HSPG2 (594)<br>TO-PRO-3 (647) | 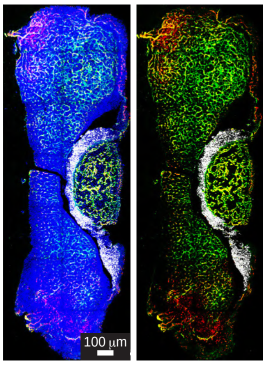 | 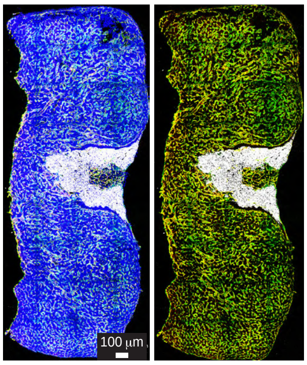 | 9 |
| CD34 (488)<br>Decorin (546)<br>Emcn (594)<br>TO-PRO-3 (647) | 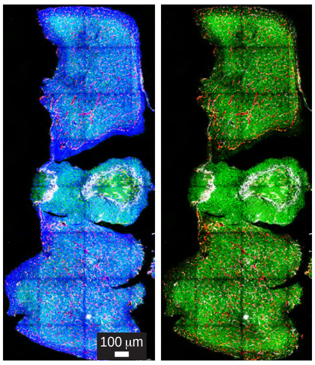 | 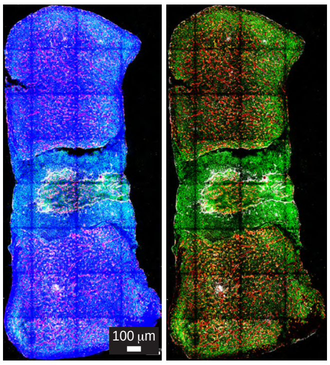 | 10 |

| Antibodies | Young | Aged | Database No. |
|---|---|---|---|
| GJA-1 (488)<br>Endoglin (546)<br>Emcn (594)<br>TO-PRO-3 (647) | 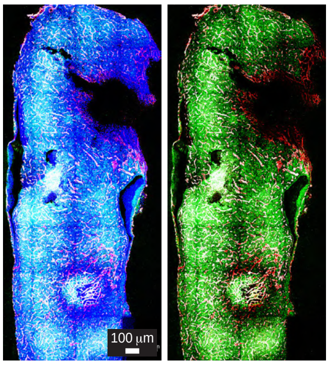 | 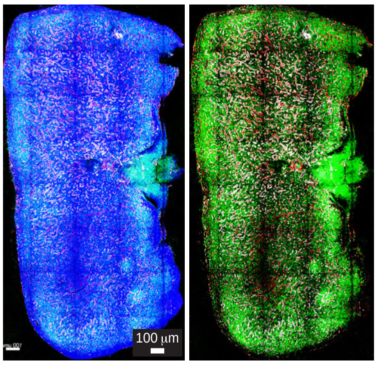 | 11 |
| GJA-1 (488)<br>α-SMA (546)<br>Emcn (594)<br>TO-PRO-3 (647) | 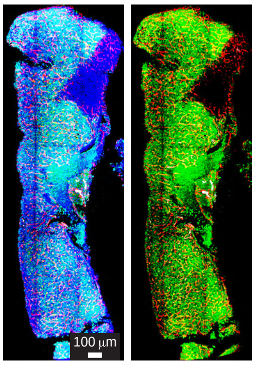 | 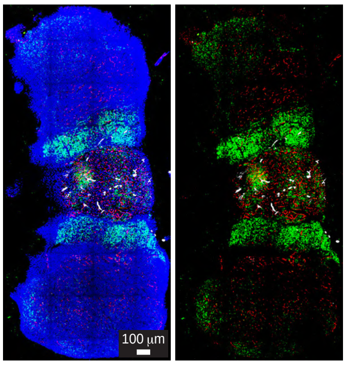 | 12 |
| HIF1α (488)<br>Decorin (546)<br>Emcn (594)<br>TO-PRO-3 (647) | 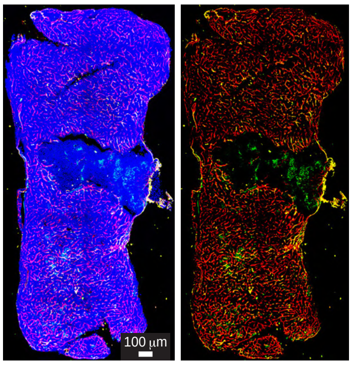 | 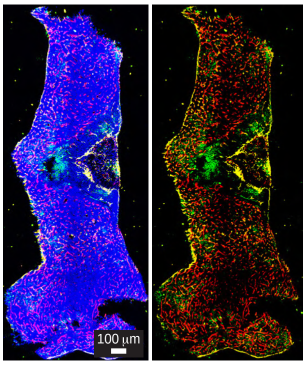 | 13 |
| Decorin (488)<br>α-SMA (546)<br>Emcn (594)<br>TO-PRO-3 (647) | 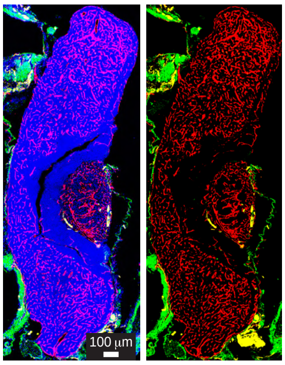 | 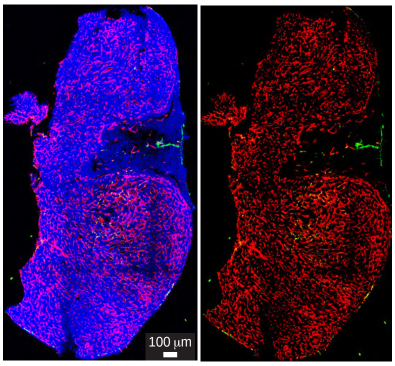 | 14 |
| α-SMA (546)<br>Emcn (594)<br>TO-PRO-3 (647) | 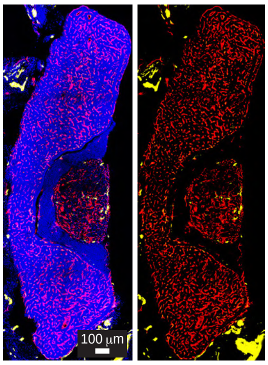 | 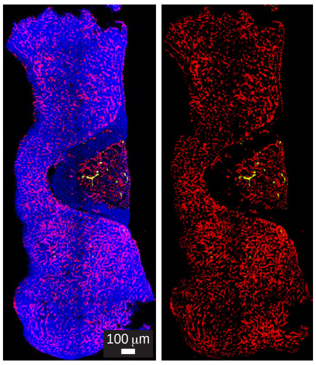 | 15 |

| Antibodies | Young | Aged | Database No. |
|---|---|---|---|
| Podocalyxin (488)<br>$\alpha$-SMA (546)<br>Emcn (594)<br>TO-PRO-3 (647) | 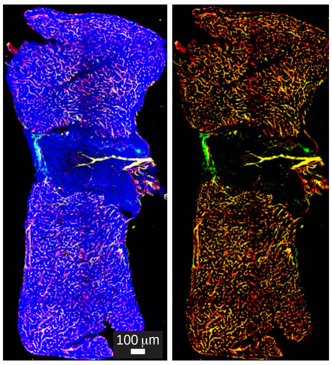 | 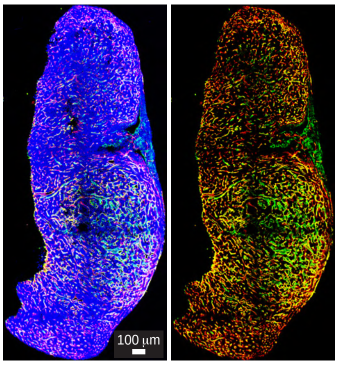 | 16 |
| Isolectin (488)<br>$\alpha$-SMA (546)<br>Emcn (594)<br>FSP-1 (647)<br>DAPI (405) | 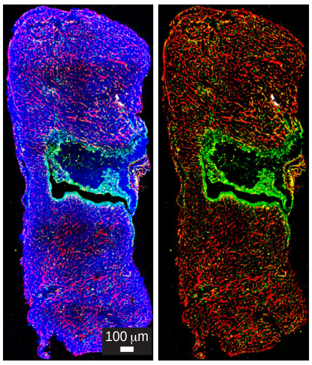 | 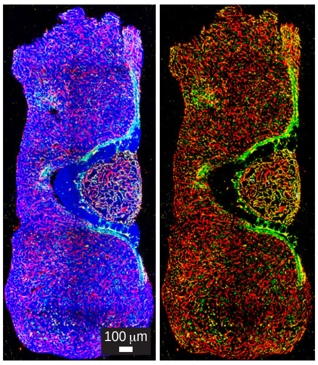 | 17 |
| Vinculin (488)<br>CD68 (546)<br>Endoglin (594)<br>Isolectin (647)<br>DAPI (405) | 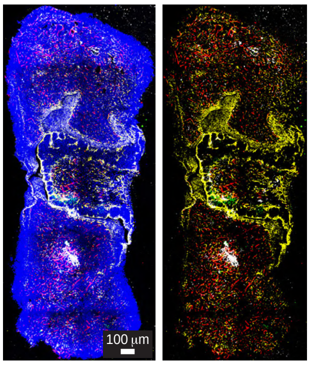 | 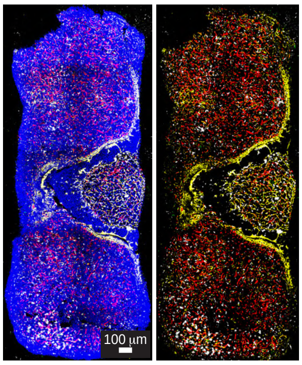 | 18 |
| FSP-1 (488)<br>Isolectin (546)<br>Emcn (594)<br>Podoplanin (647)<br>DAPI (405) | 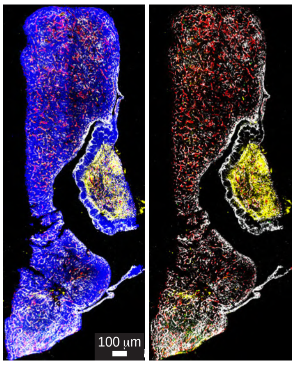 | 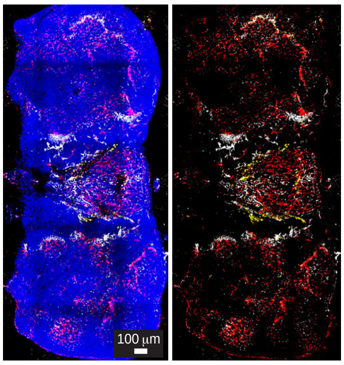 | 19 |
| Claudin-5 (488)<br>Isolectin (546)<br>Emcn (594)<br>Endoglycan (647)<br>DAPI (405) | 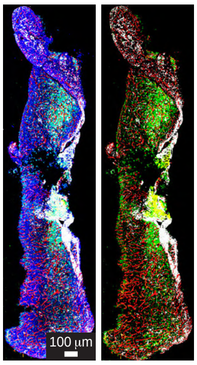 | 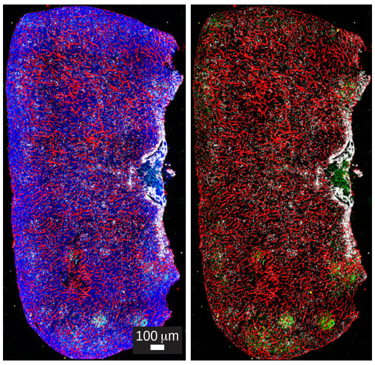 | 20 |

| Antibodies | Young | Aged | Database No. |
|---|---|---|---|
| Vimentin (488)<br>Isolectin (546)<br>CD102 (594)<br>CXCR4 (647)<br>DAPI (405) | 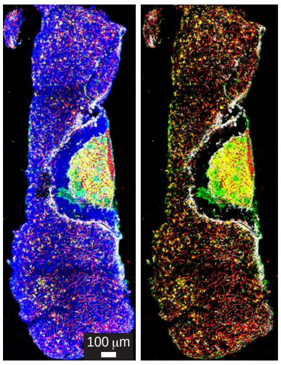  | 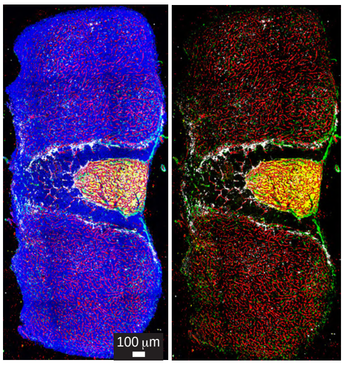  | 21 |
| Laminin (488)<br>Isolectin (546)<br>Emcn (594)<br>c-kit (647)<br>DAPI (405) | 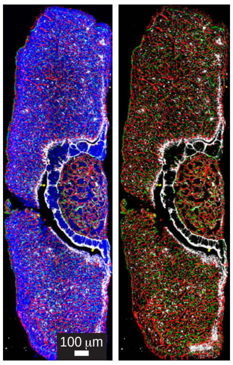  | 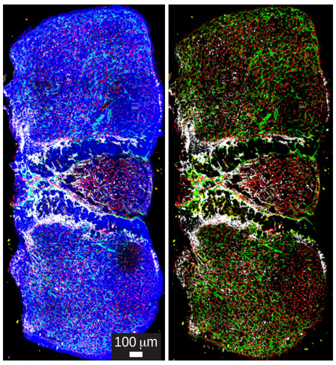  | 22 |
| Isolectin (488)<br>P-selectin (546)<br>Emcn (594)<br>Vinculin (647)<br>DAPI (405) | 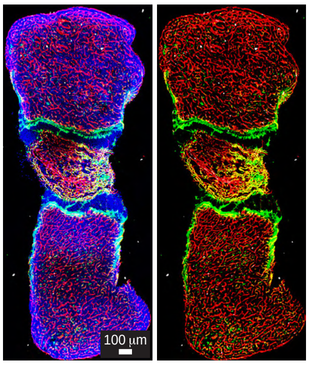  | 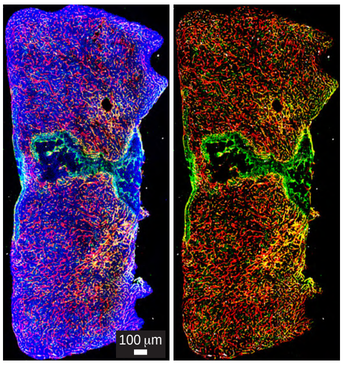  | 23 |
| CD31 (488)<br>PDGFRβ (546)<br>Emcn (594)<br>TO-PRO-3 (647) | 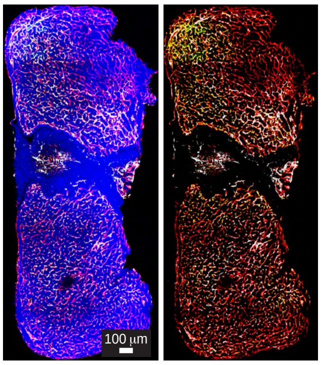  | 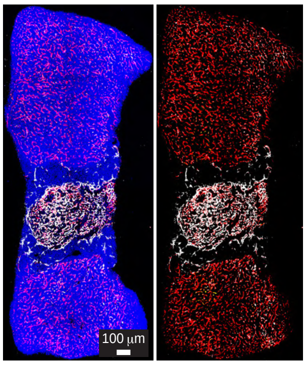  | 24 |
| PDGFRα (546)<br>HSPG2 (594)<br>TO-PRO-3 (647) | 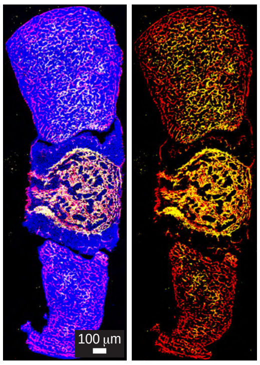  | 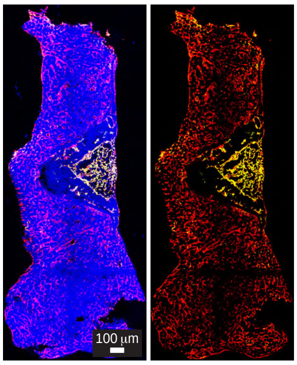  | 25 |

| Antibodies | Young | Aged | Database No. |
|---|---|---|---|
| HIF1α (488)<br>Endoglin (546)<br>HSPG2 (594)<br>TO-PRO-3 (647) | 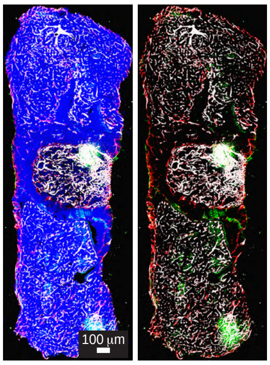 | 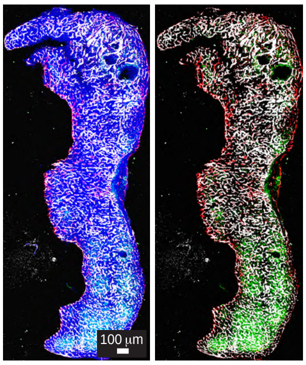 | 26 |
| CD34 (488)<br>Emcn (594)<br>TO-PRO-3 (647) | 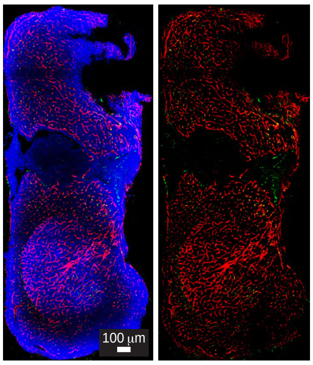 | 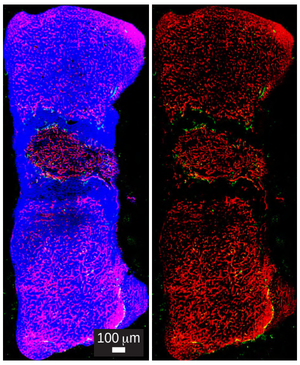 | 27 |
| LYVE-1 (488)<br>Emcn (594)<br>TO-PRO-3 (647) | 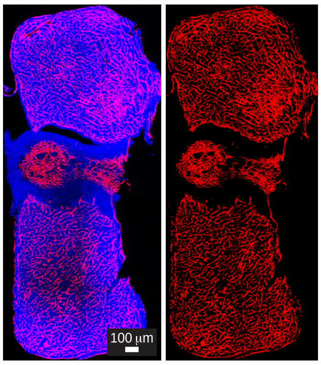 | 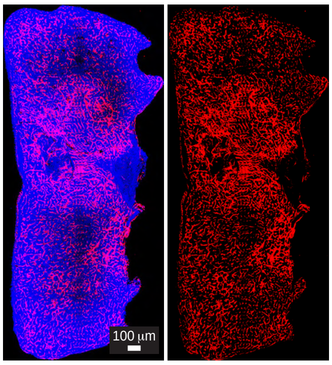 | 28 |
| Fibronectin (488)<br>Emcn (594)<br>TO-PRO-3 (647) | 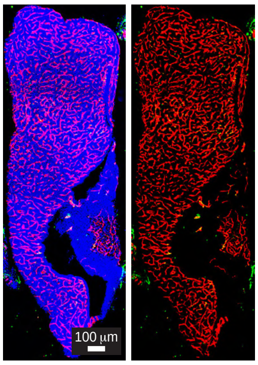 | 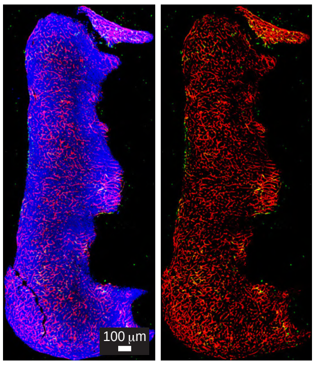 | 29 |
| Isolectin (488)<br>Emcn (594)<br>Smoothelin (647)<br>DAPI (405) | 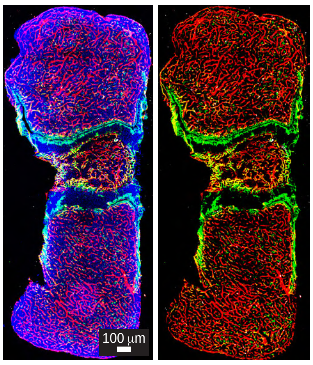 | 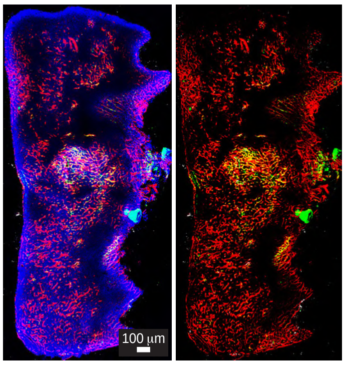 | 30 |

| Antibodies | Young | Aged | Database No. |
|---|---|---|---|
| Caveolin-1 (488)<br>Endoglin (546)<br>CD45 (594)<br>TO-PRO-3 (647) | 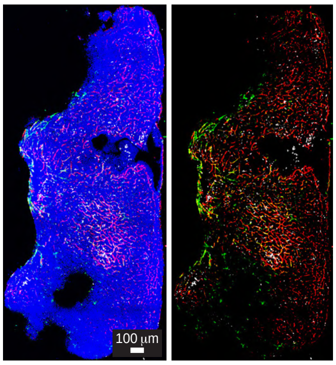 | 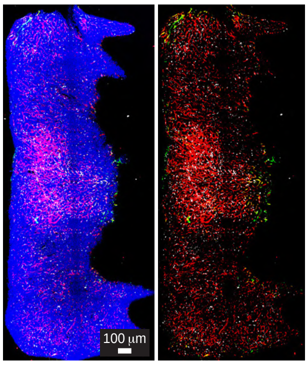 | 31 |
| Laminin (488)<br>Endoglin (546)<br>F4/80 (594)<br>DAPI (405) | 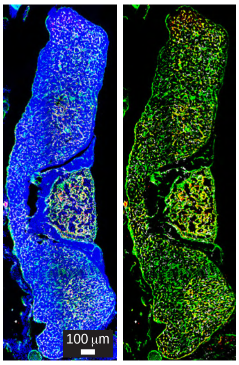 | 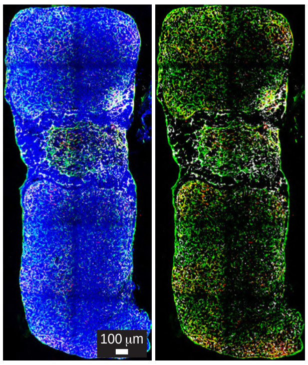 | 32 |
| VEGFA (488)<br>Emcn (546)<br>TO-PRO-3 (405) | 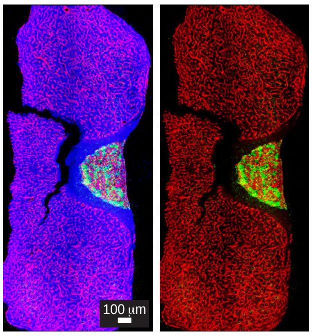 | 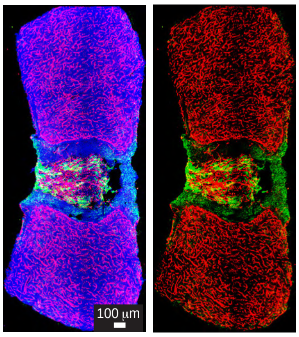 | 75, 76 |

**Appendix Table S7**. Single-cell 3D mapping image database of young versus aged murine testes

| Antibodies | Young | Aged | Database No. |
|---|---|---|---|
| GJA-1 (488)<br>α-SMA (546)<br>Emcn (594)<br>TO-PRO-3 (647) | 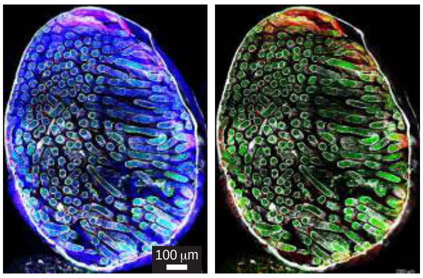 | 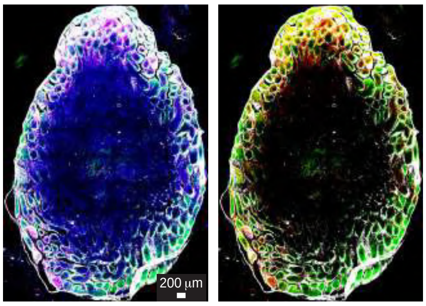 | 1 |
| GJA-1 (488)<br>Tie-2 (546)<br>HSPG2 (594)<br>TO-PRO-3 (647) | 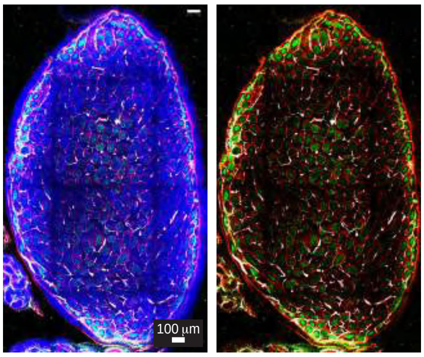 | 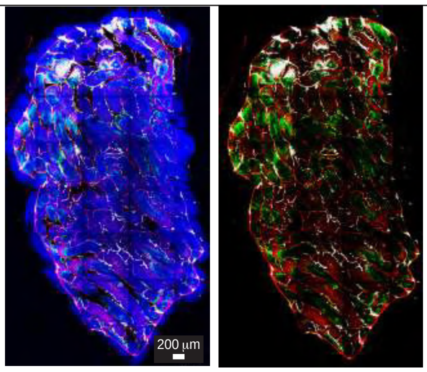 | 2 |
| Isolectin (488)<br>α-SMA (546)<br>Emcn (594)<br>Desmin (647)<br>DAPI (405) | 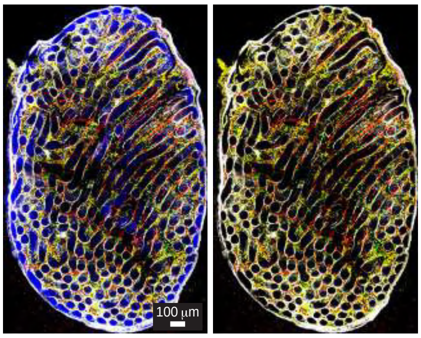 | 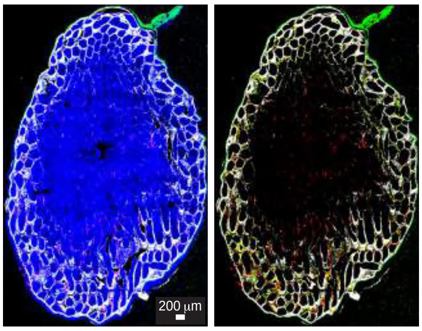 | 3 |
| Smoothelin (488)<br>Isolectin (546)<br>Emcn (594)<br>Decorin (647)<br>DAPI (405) | 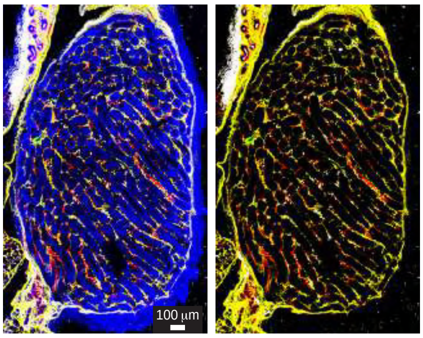 | 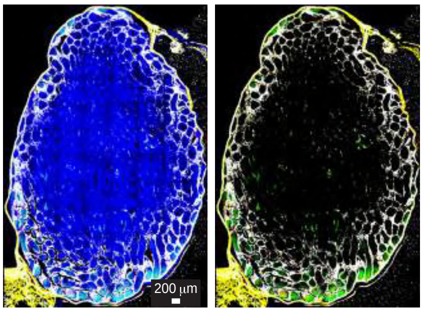 | 4 |
| HIF1α (488)<br>Endoglin (546)<br>HSPG2 (594)<br>TO-PRO-3 (647) | 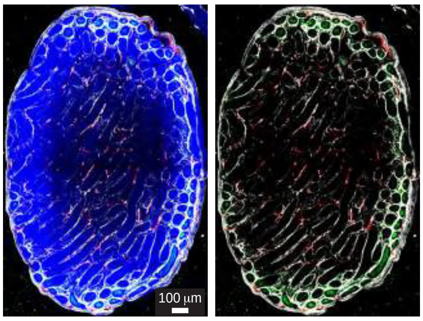 | 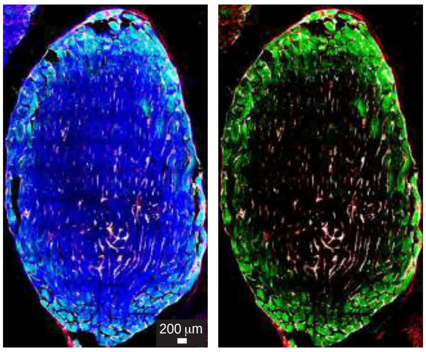 | 5 |

| Antibodies | Young | Aged | Database No. |
|---|---|---|---|
| Isolectin (488)<br>ESM-1 (546)<br>Emcn (594)<br>SM22α (647)<br>DAPI (405) | 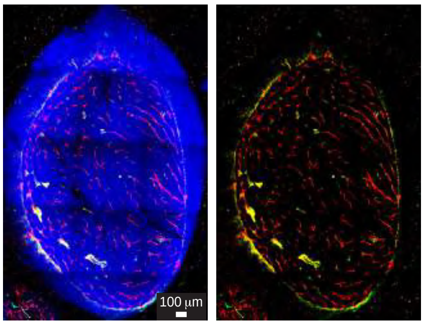 | 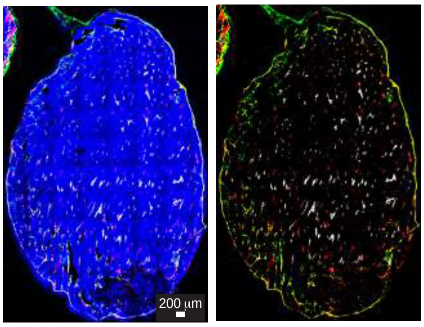 | 6 |
| Smoothelin (488)<br>Podoplanin (546)<br>HSPG2 (594)<br>TO-PRO-3 (647) | 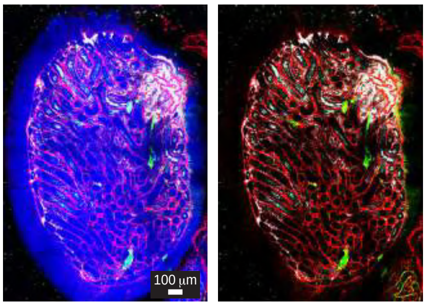 | 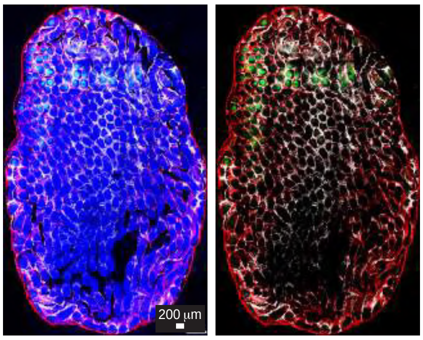 | 7 |
| FSP-1 (488)<br>Endoglin (546)<br>Emcn (594)<br>DAPI (405) | 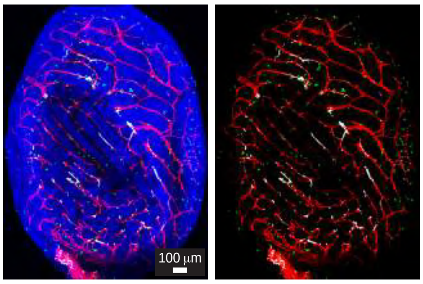 | 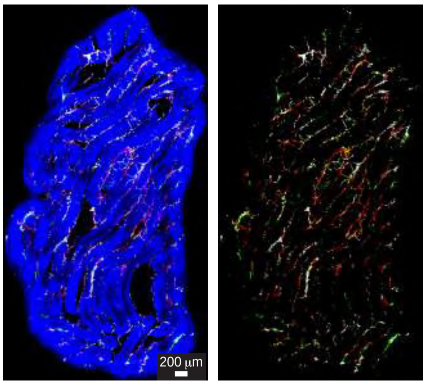 | 8 |
| Laminin (488)<br>Endoglin (546)<br>F4/80 (594)<br>DAPI (405) | 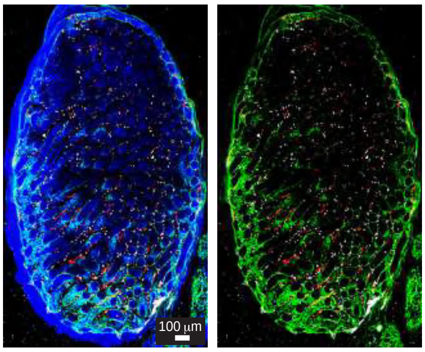 | 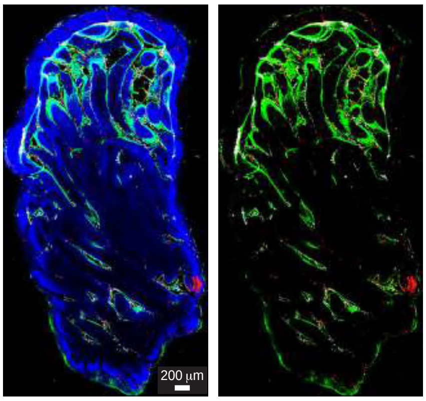 | 9 |
| HIF1α (488)<br>Decorin (546)<br>Emcn (594)<br>TO-PRO-3 (647) | 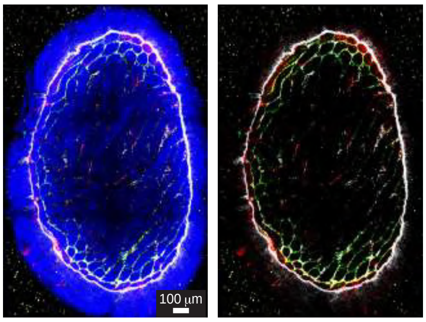 | 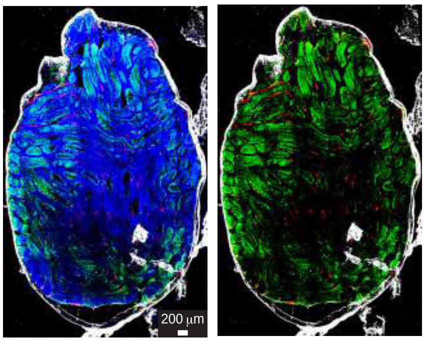 | 10 |

| Antibodies | Young | Aged | Database No. |
|---|---|---|---|
| CD31 (488)<br>Podoplanin (546)<br>PLVAP (594)<br>TO-PRO-3 (647) | 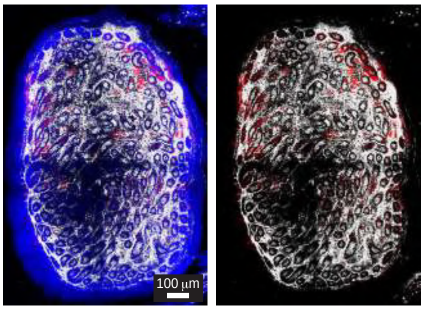  | 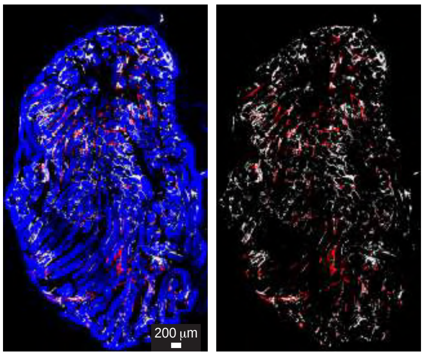  | 11 |
| FSP-1 (488)<br>Isolectin (546)<br>Emcn (594)<br>Podoplanin (647)<br>DAPI (405) | 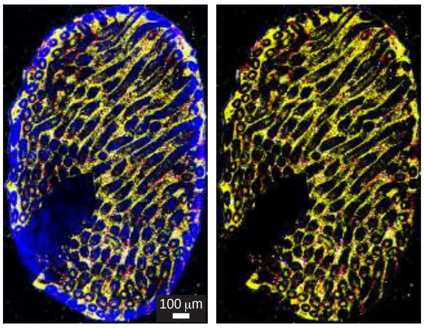  | 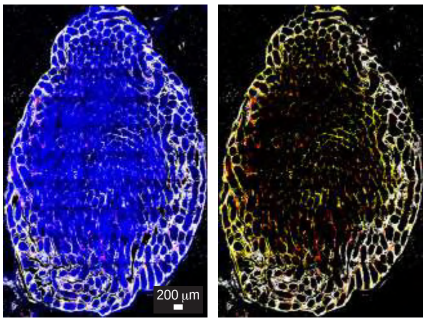  | 12 |
| Caveolin-1 (488)<br>VEGFR3 (546)<br>HSPG2 (594)<br>TO-PRO-3 (647) | 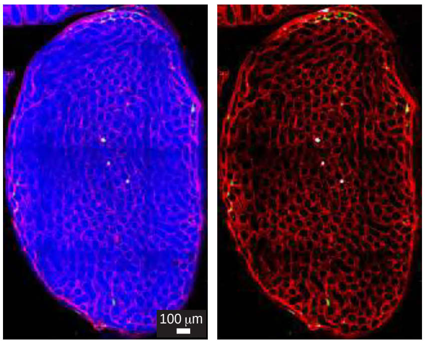  | 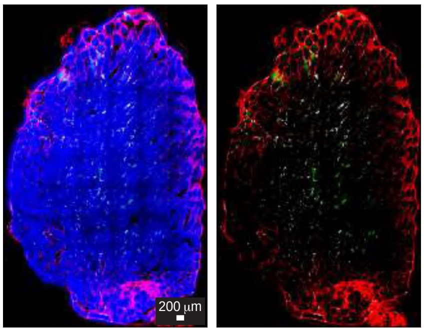  | 13 |
| VEGFR3 (488)<br>Caveolin-1 (546)<br>HSPG2 (594)<br>TO-PRO-3 (647) | 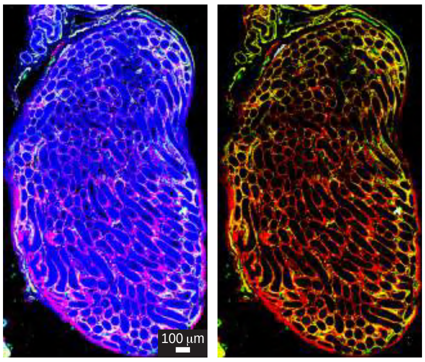  | 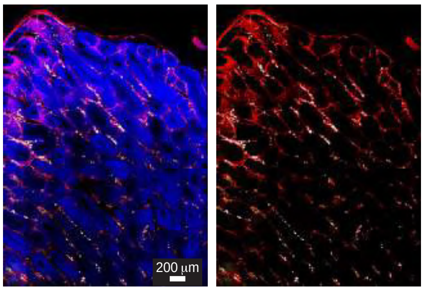  | 14 |
| Desmin (488)<br>VCAM-1 (546)<br>HSPG2 (594)<br>TO-PRO-3 (647) | 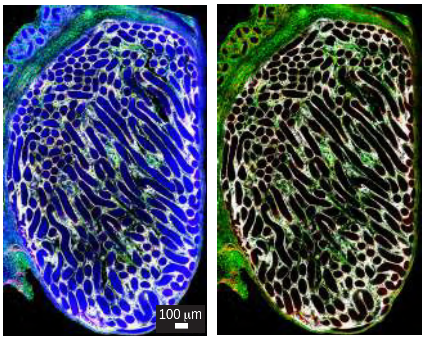  | 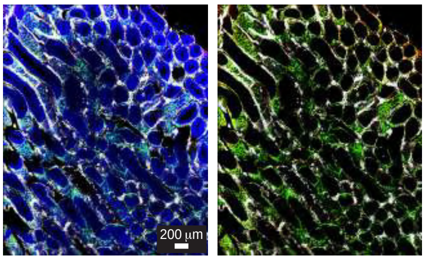  | 15 |

| Antibodies | Young | Aged | Database No. |
|---|---|---|---|
| GJA-1 (488)<br>FABP4 (546)<br>HSPG2 (594)<br>TO-PRO-3 (647) | 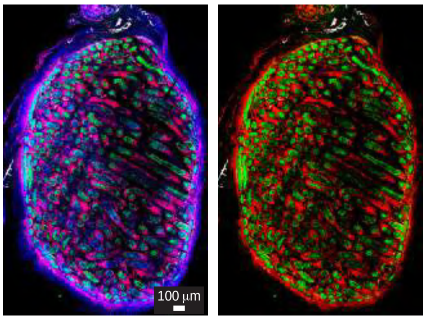 | 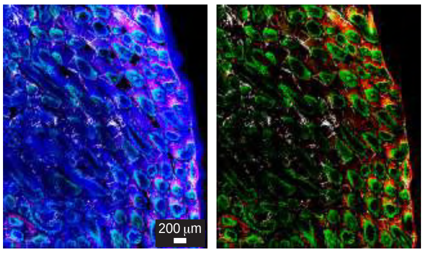 | 16 |
| Podoplanin (488)<br>NG2 (546)<br>HSPG2 (594)<br>TO-PRO-3 (647) | 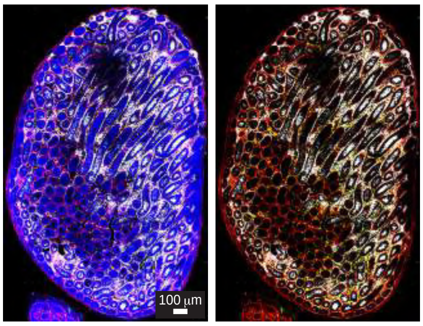 | 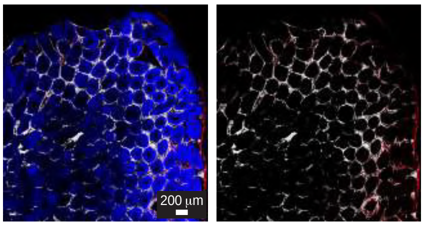 | 17 |
| PDGFRα (488)<br>CD31 (546)<br>HSPG2 (594)<br>TO-PRO-3 (647) | 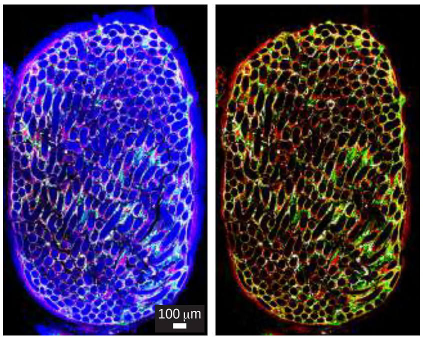 | 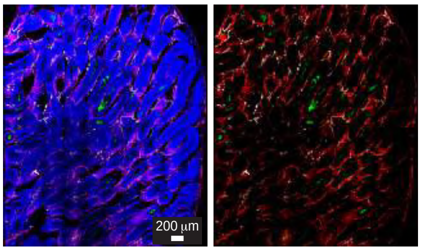 | 18 |
| Collagen IV (488)<br>Sca-1 (546)<br>F4/80 (594)<br>TO-PRO-3 (647) | 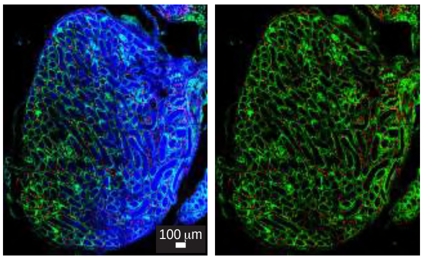 | 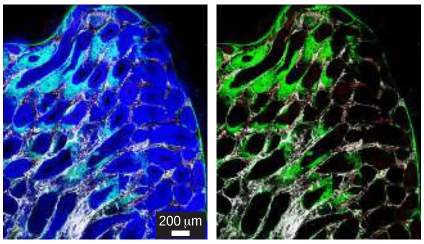 | 19 |
| Vinculin (488)<br>α-SMA (546)<br>HSPG2 (594)<br>TO-PRO-3 (647) | 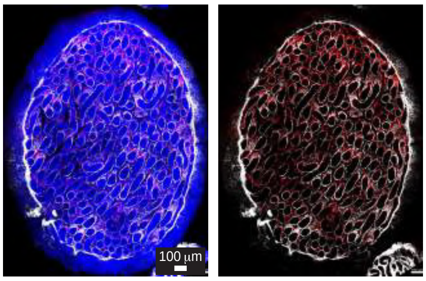 | 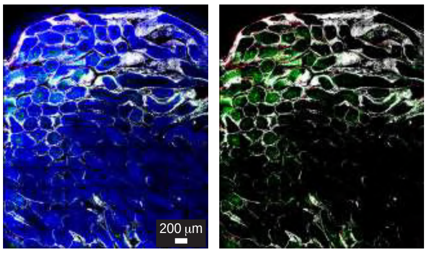 | 20 |

| Antibodies | Young | Aged | Database No. |
|---|---|---|---|
| eIF2α (488)<br>c-kit (546)<br>HSPG2 (594)<br>TO-PRO-3 (647) | 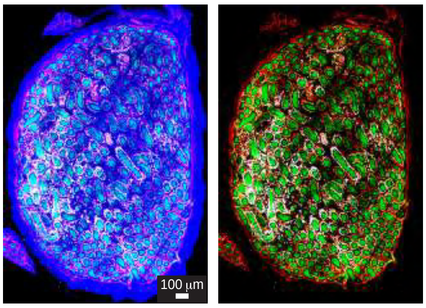  | 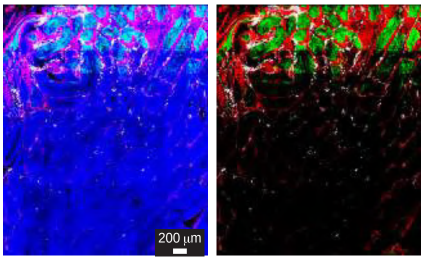  | 21 |
| Collagen IV (488)<br>Endoglycan (546)<br>CD68 (594)<br>TO-PRO-3 (647) | 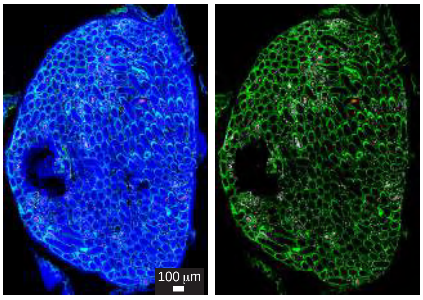  | 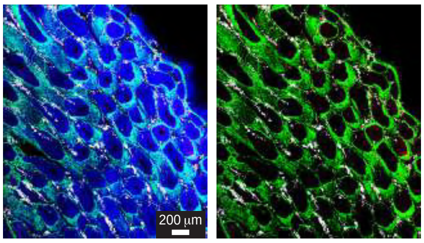  | 22 |
| Fibronectin (488)<br>Dll4 (546)<br>HSPG2 (594)<br>TO-PRO-3 (647) | 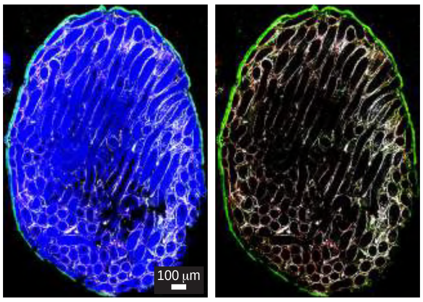  | 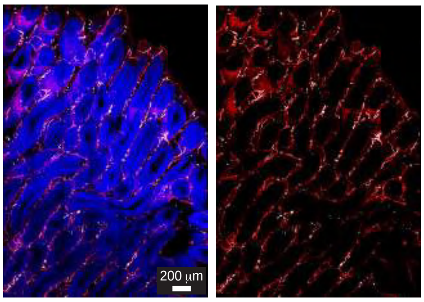  | 23 |
| CD34 (488)<br>Podocalyxin (546)<br>HSPG2 (594)<br>TO-PRO-3 (647) | 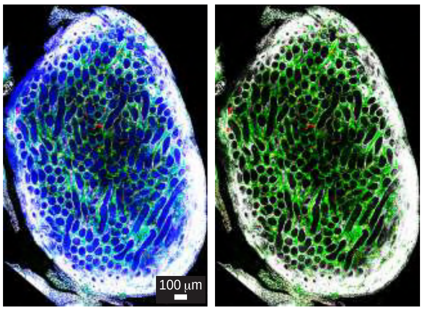  | 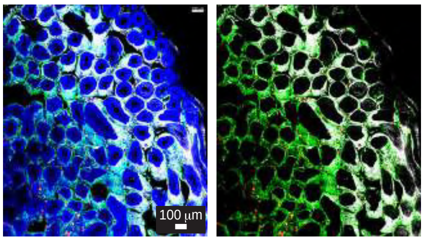  | 24 |
| SM22α (488)<br>CXCR4 (546)<br>HSPG2 (594)<br>TO-PRO-3 (647) | 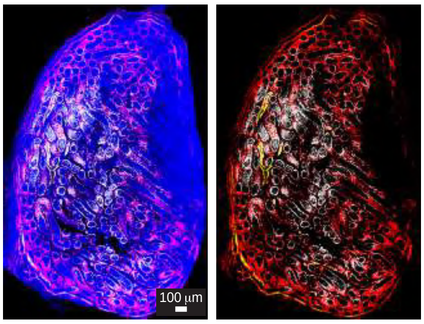  | 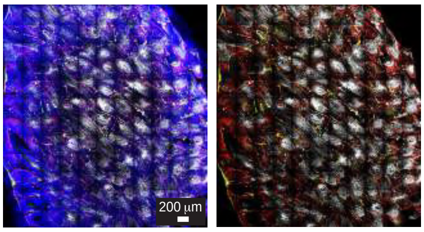  | 25 |

| Antibodies | Young | Aged | Database No. |
|---|---|---|---|
| GJA-1 (488)<br>FABP4 (546)<br>HSPG2 (594)<br>TO-PRO-3 (647) | 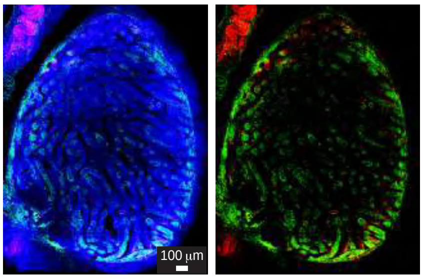 | 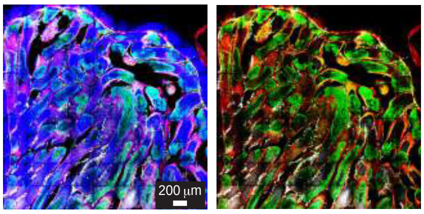 | 26 |
| eIF2α (488)<br>ICAM-1 (546)<br>Emcn (594)<br>TO-PRO-3 (647) | 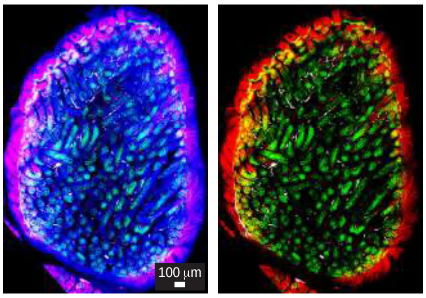 | 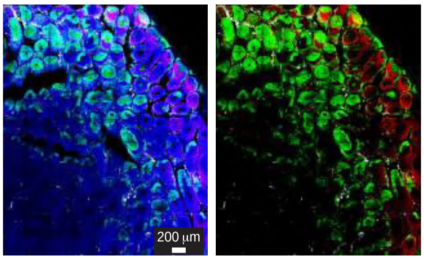 | 27 |
| eIF2α (488)<br>ICAM-1 (546)<br>Emcn (594)<br>TO-PRO-3 (647) | 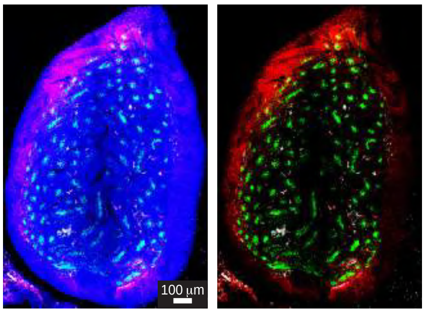 | 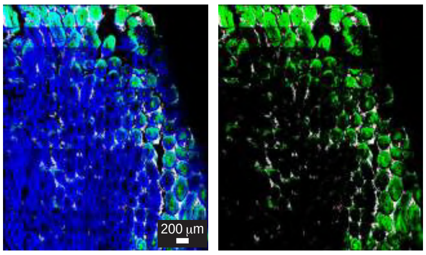 | 28 |
| PDGFRβ (488)<br>Tie-2 (546)<br>Emcn (594)<br>TO-PRO-3 (647) | 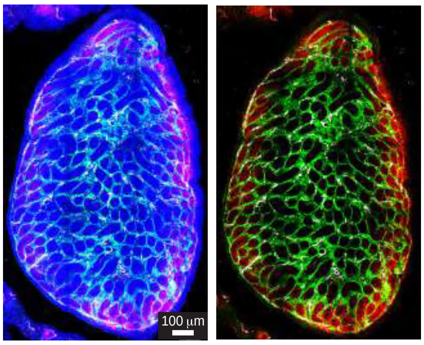 | 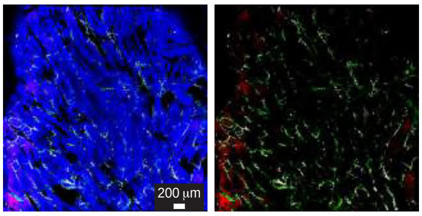 | 29 |
| PDGFRβ (488)<br>CD31 (546)<br>F4/80 (594)<br>TO-PRO-3 (647) | 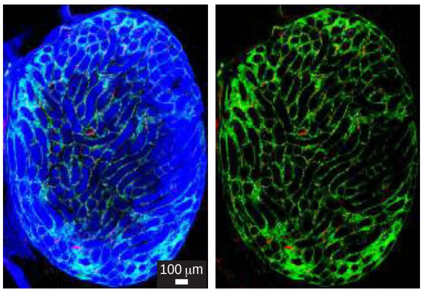 | 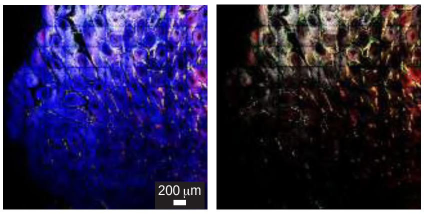 | 30 |

| Antibodies | Young | Aged | Database No. |
|---|---|---|---|
| VCAM-1 (488)<br>Emcn (594)<br>TO-PRO-3 (647) | 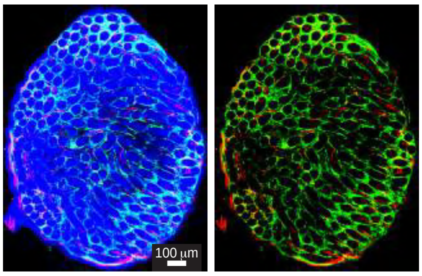 | 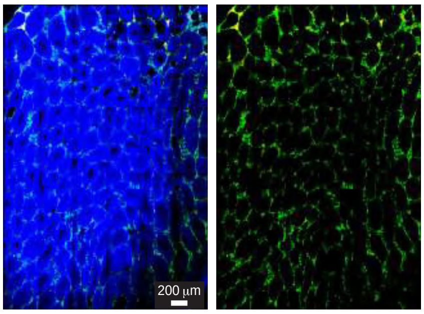 | 31 |
| Smoothelin (488)<br>BCAM (546)<br>HSPG2 (594)<br>TO-PRO-3 (647) | 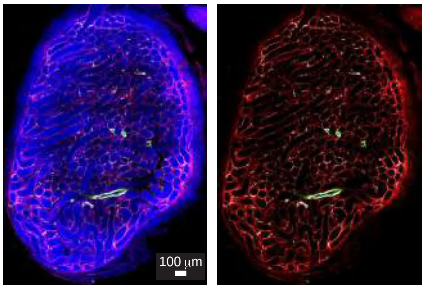 | 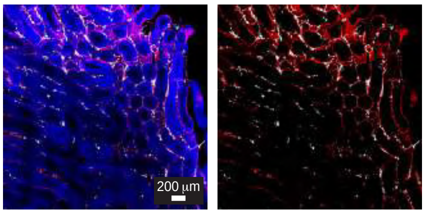 | 32 |
| Smoothelin (488)<br>PLVAP (546)<br>Emcn (594)<br>TO-PRO-3 (647) | 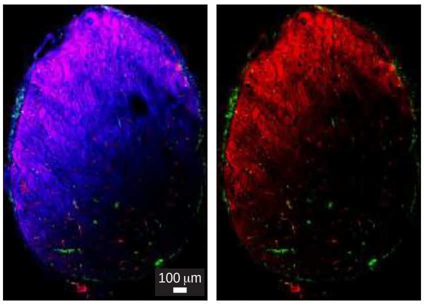 | 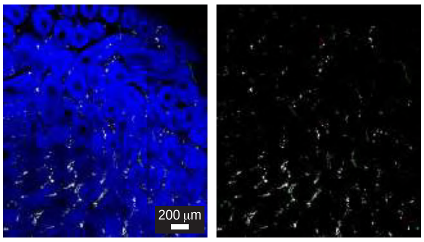 | 33 |
| Laminin (488)<br>VCAM-1 (546)<br>CD102 (594)<br>TO-PRO-3 (647) | 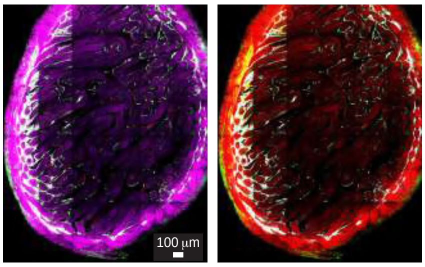 | 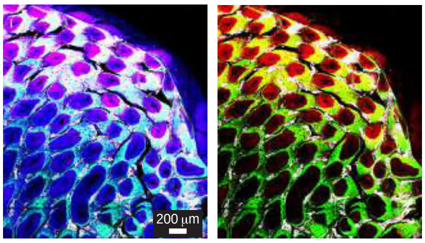 | 34 |
| eIF2α (488)<br>c-kit (546)<br>CD102 (594)<br>TO-PRO-3 (647) | 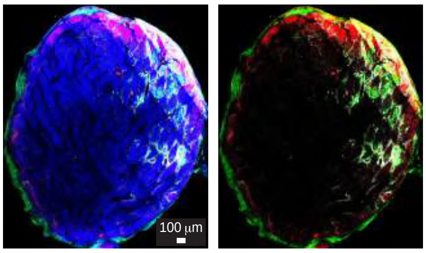 | 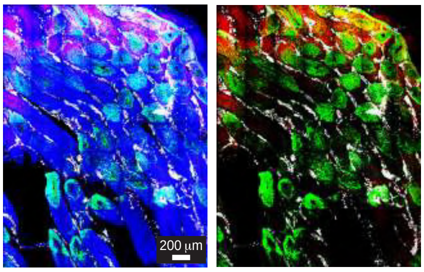 | 35 |

| Antibodies | Young | Aged | Database No. |
|---|---|---|---|
| SM22α (488)<br>CXCR4 (546)<br>Emcn (594)<br>TO-PRO-3 (647) | 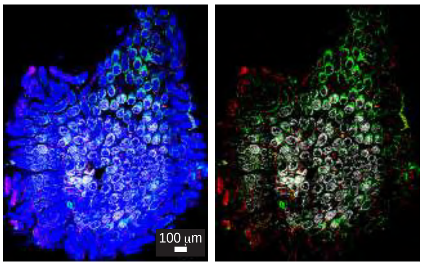 | 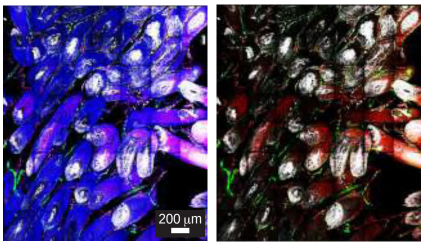 | 36 |
| SM22α (488)<br>Fibronectin (546)<br>Emcn (594)<br>TO-PRO-3 (647) | 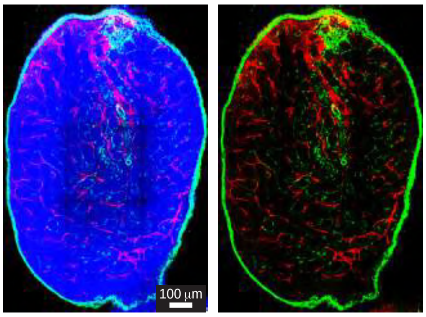 | 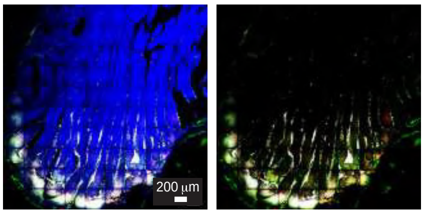 | 37 |
| P-selectin (546)<br>HSPG2 (594)<br>TO-PRO-3 (647) | 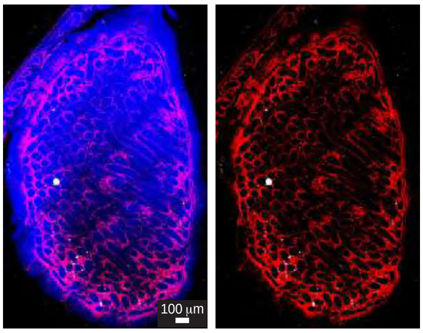 | 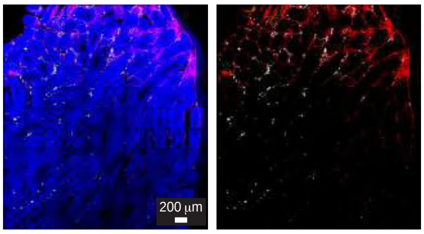 | 38 |
| NG2 (488)<br>BCAM (546)<br>Emcn (594)<br>TO-PRO-3 (647) | 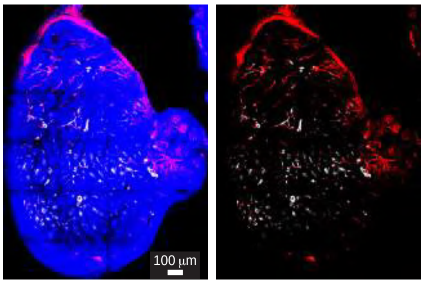 | 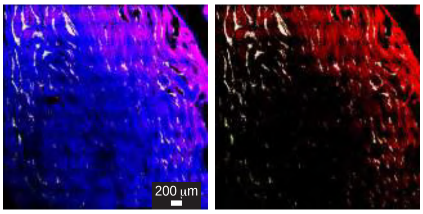 | 39 |
| VEGFA (488)<br>Emcn (594)<br>TO-PRO-3 (647) | 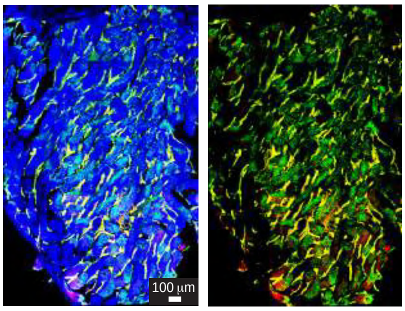 | 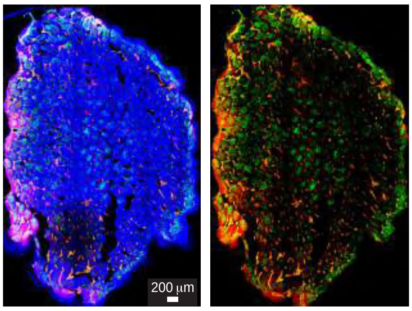 | 178, 179 |

**Appendix Table S8**. Single-cell 3D mapping image database of murine testes with various niche markers

| Antibodies | Young | Antibodies | Young |
|---|---|---|---|
| eIF2α (488)<br>c-kit (546)<br>HSPG2 (594)<br>TO-PRO-3 (647) | 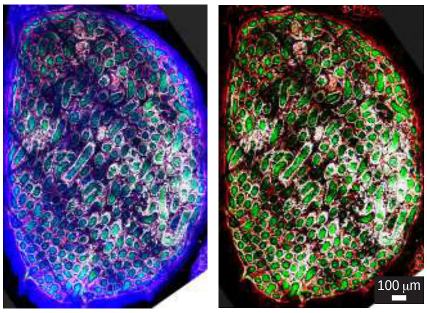 | PDGFRα (488)<br>CXCR4 (546)<br>Emcn (594)<br>TO-PRO-3 (647) | 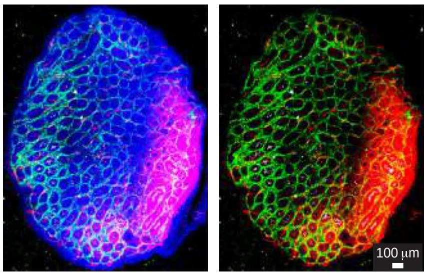 |
| GJA-1 (488)<br>FABP4 (546)<br>HSPG2 (594)<br>TO-PRO-3 (647) | 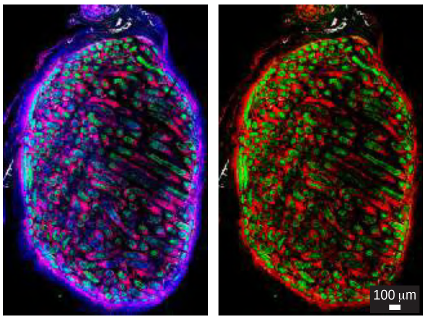 | P-selectin (546)<br>HSPG2 (594)<br>TO-PRO-3 (647) | 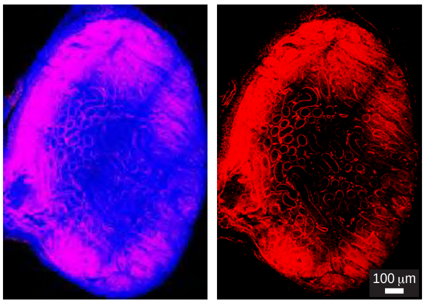 |
| Claudin-5 (488)<br>c-kit (546)<br>CD102 (594)<br>TO-PRO-3 (647) | 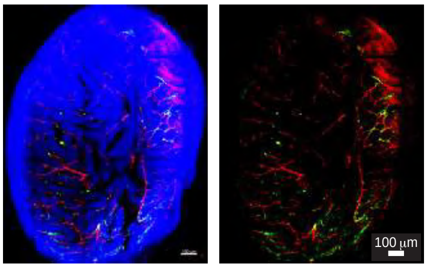 | PDGFRβ (488)<br>Tie-2 (546)<br>Emcn (594)<br>TO-PRO-3 (647) | 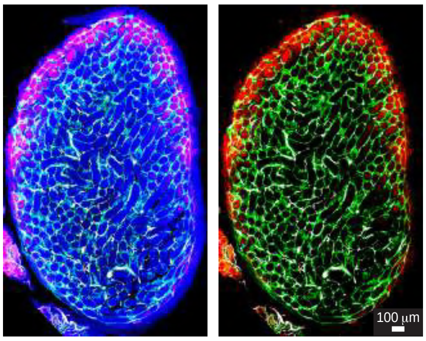 |
| Emcn (594)<br>TO-PRO-3 (647) | 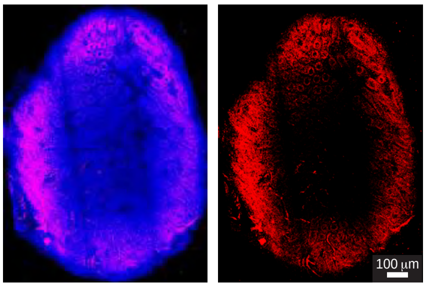 | | |

**Appendix Table S9**. Single-cell 3D mapping image database of young versus aged murine pancreases

| Antibodies | Young | Aged | Database No. |
|---|---|---|---|
| EPCR (488)<br>$\alpha$-SMA (546)<br>HSPG2 (594)<br>TO-PRO-3 (647) | 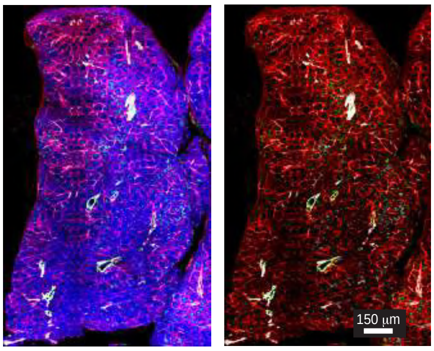 | 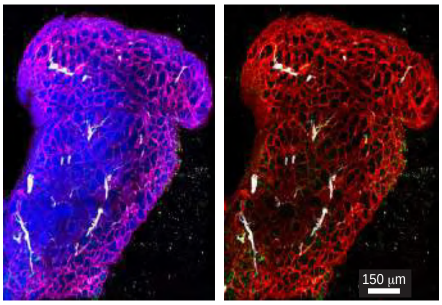 | 1 |
| eIF2$\alpha$ (488)<br>PDGFR$\alpha$ (546)<br>HSPG2 (594)<br>TO-PRO-3 (647) | 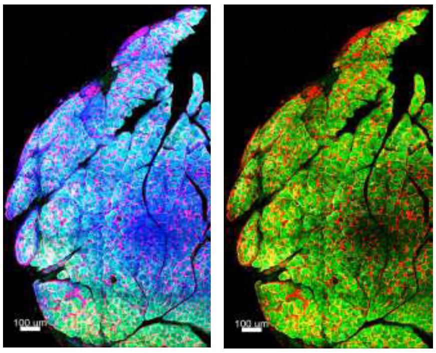 | 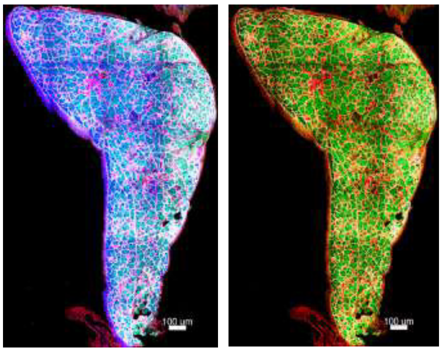 | 2 |
| Perilipin (488)<br>FABP4 (546)<br>Emcn (594)<br>TO-PRO-3 (647) | 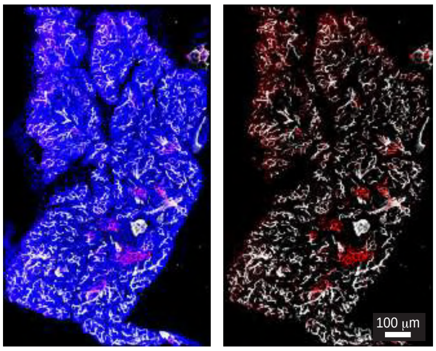 | 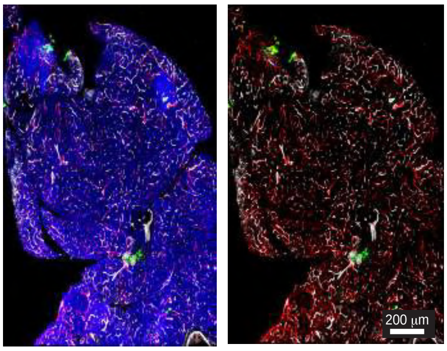 | 3 |
| Fibronectin (488)<br>Endoglin (546)<br>CD68 (594)<br>TO-PRO-3 (647) | 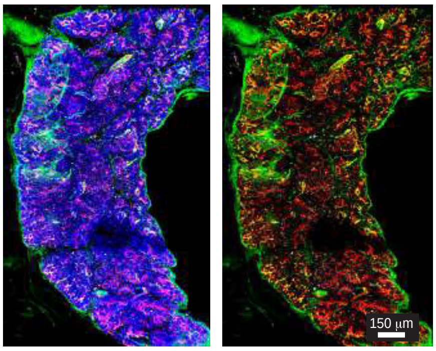 | 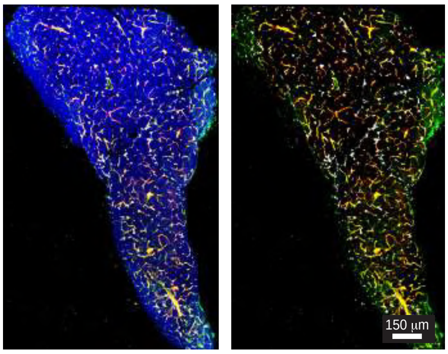 | 4 |
| Vimentin (488)<br>NG2 (546)<br>Emcn (594)<br>TO-PRO-3 (647) | 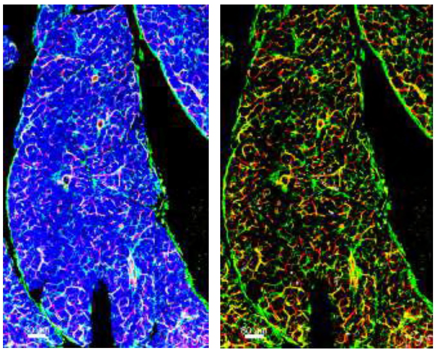 | 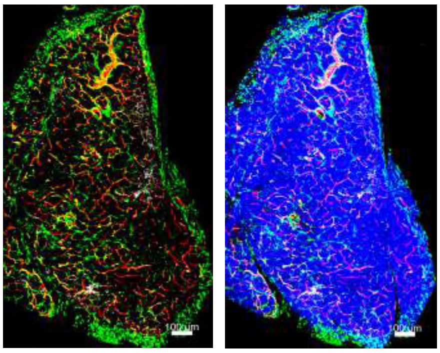 | 5 |

| Antibodies | Young | Aged | Database No. |
|---|---|---|---|
| Caveolin-1 (488)<br>Endoglin (546)<br>F4/80 (594)<br>TO-PRO-3 (647) | 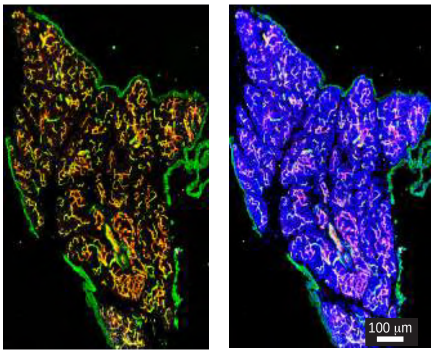  | 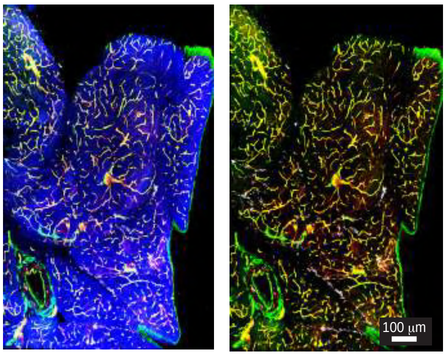  | 6 |
| Collagen IV (488)<br>P-selectin (546)<br>CD102 (594)<br>TO-PRO-3 (647) | 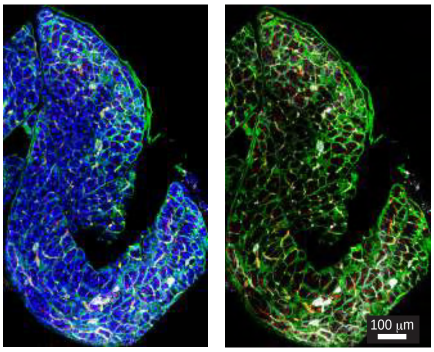  | 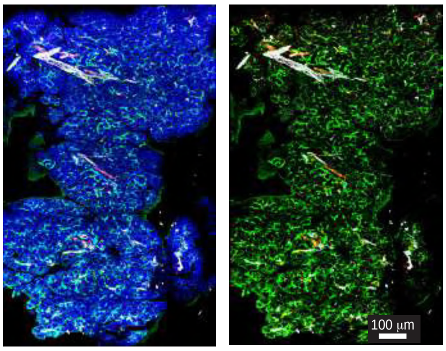  | 7 |
| PDGFRα (488)<br>Sca-1 (546)<br>Emcn (594)<br>TO-PRO-3 (647) | 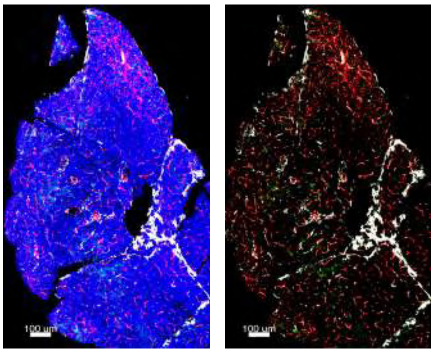 | 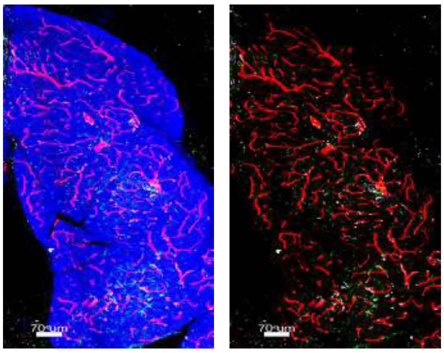 | 8 |
| Smoothelin (488)<br>BCAM (546)<br>Emcn(594)<br>TO-PRO-3 (647) | 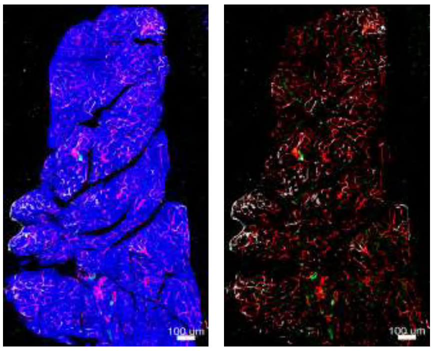 | 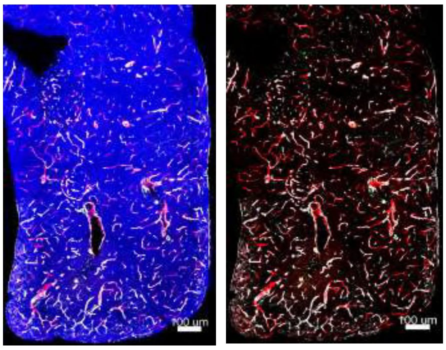 | 9 |
| SM22α (488)<br>Endoglin (546)<br>TO-PRO-3 (647) | 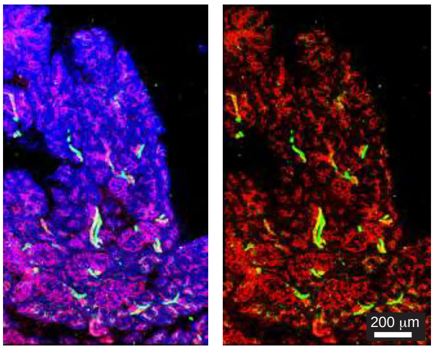  | 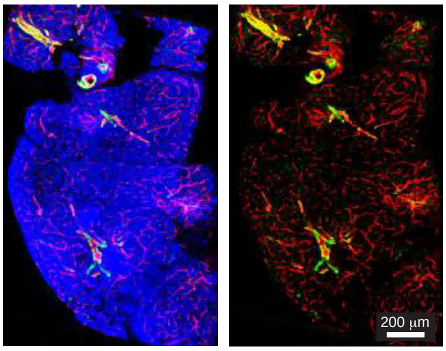  | 10 |

| Antibodies | Young | Aged | Database No. |
|---|---|---|---|
| Perilipin (488)<br>Tie-2 (546)<br>Emcn (594)<br>TO-PRO-3 (647) | 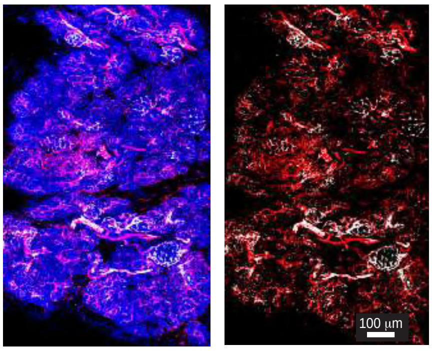 | 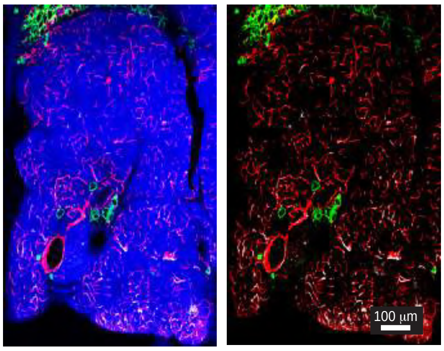 | 11 |
| Endoglin (546)<br>CD45(594)<br>TO-PRO-3 (647) | 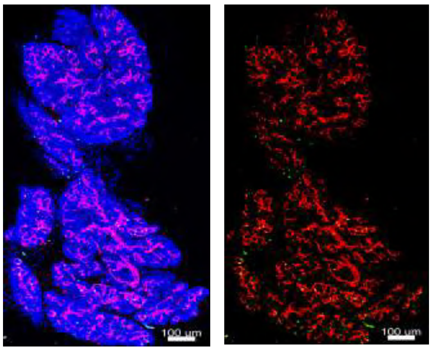 | 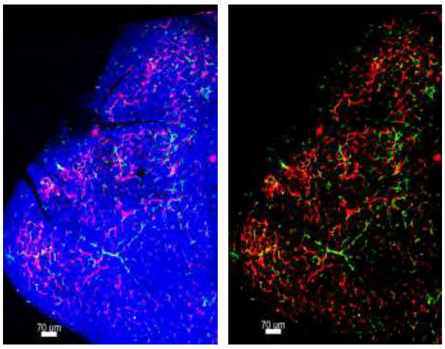 | 12 |
| GJA-1 (488)<br>Endoglin (546)<br>CD102 (594)<br>TO-PRO-3 (647) | 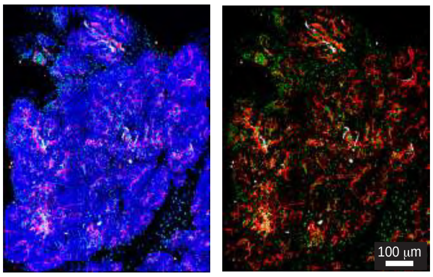 | 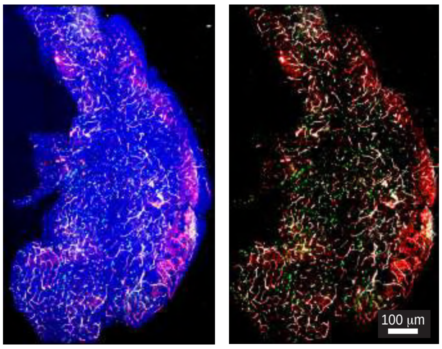 | 13 |
| FSP1 (488)<br>Isolectin (546)<br>Emcn (594)<br>Decorin (647)<br>DAPI (405) | 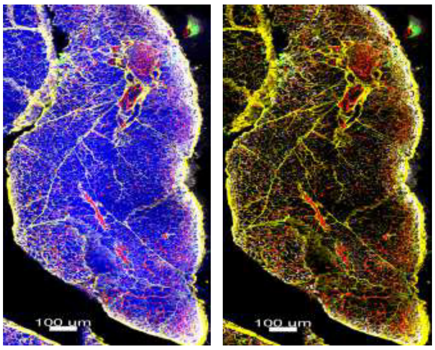 | 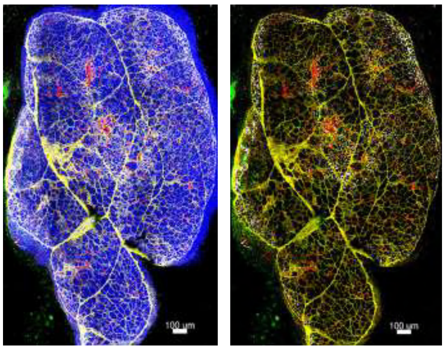 | 14 |
| Hif1α (488)<br>Decorin (546)<br>Emcn (594)<br>TO-PRO-3 (647) | 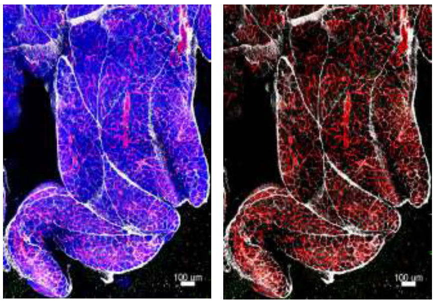 | 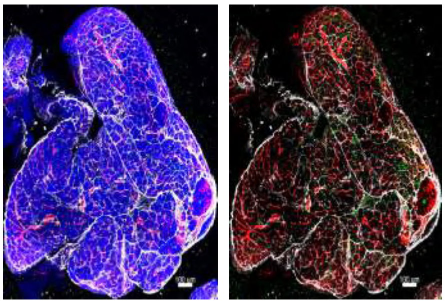 | 15 |

| Antibodies | Young | Aged | Database No. |
|---|---|---|---|
| CD31 (488)<br>Emcn (546)<br>TO-PRO-3 (647) | 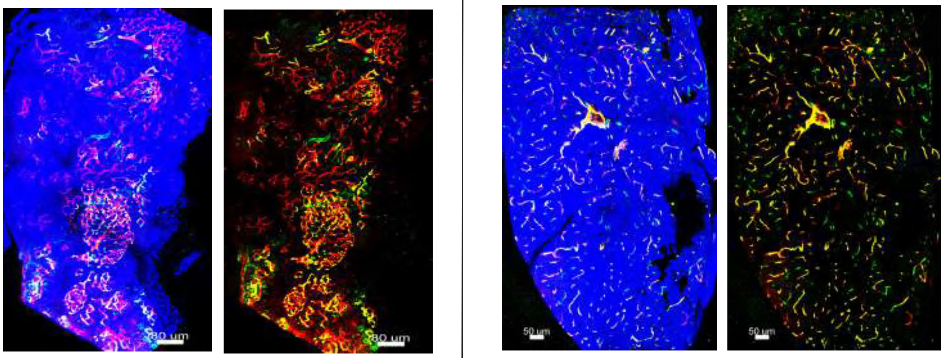 | | 16 |
| P-selectin (488)<br>Emcn (546)<br>TO-PRO-3 (647) | 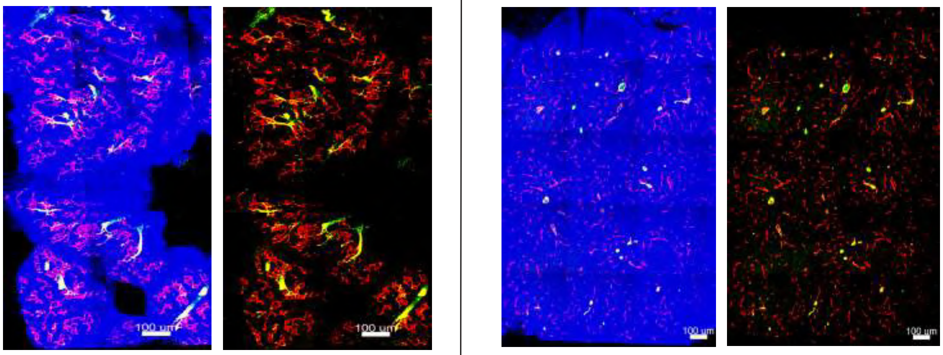 | | 17 |
| Podocalyxin (546)<br>Emcn(594)<br>TO-PRO-3 (647) | 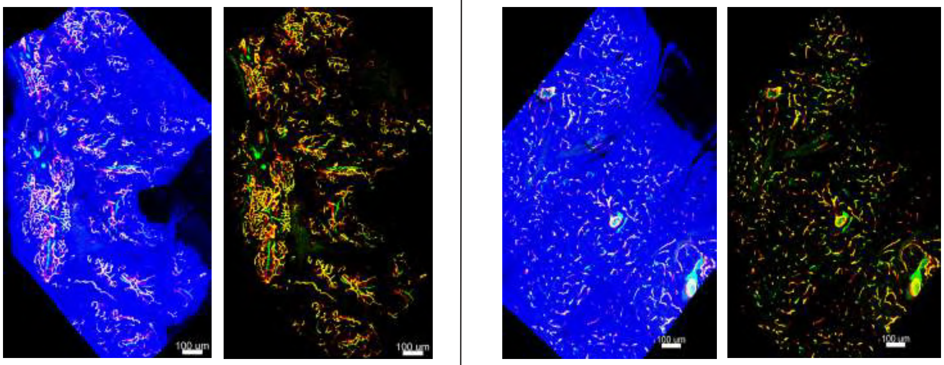 | | 18 |
| VCAM-1 (488)<br>Emcn (594)<br>TO-PRO-3(405) | 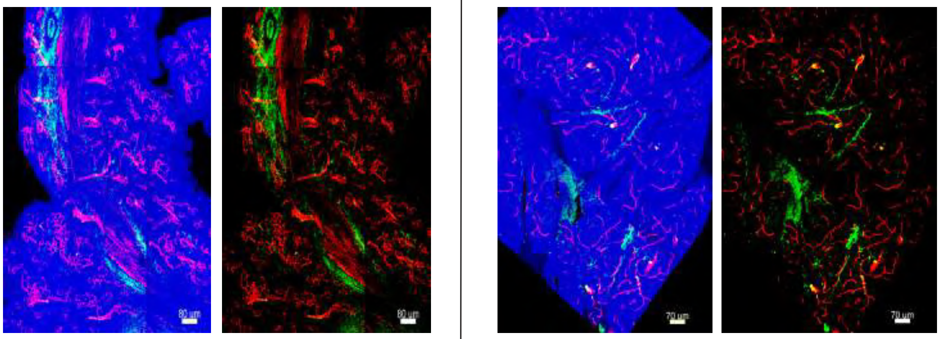 | | 19 |
| FABP4 (488)<br>Emcn (546)<br>TO-PRO-3 (647) | 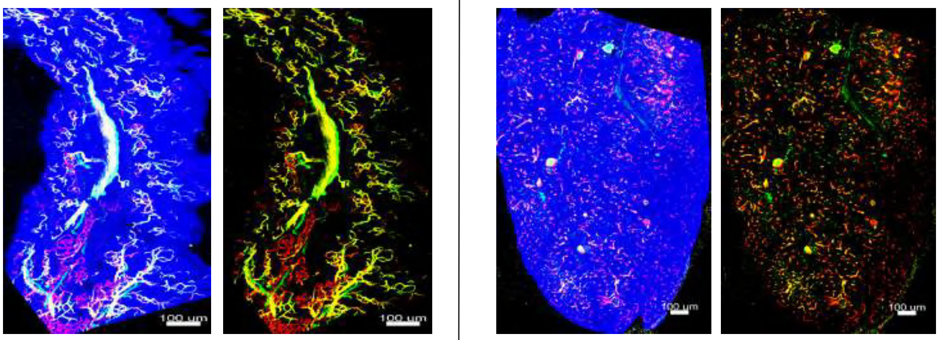 | | 20 |

**Appendix Table S10**. Single-cell 3D mapping image database of murine pancreases with various niche markers

| Antibodies | Young | Antibodies | Young |
|---|---|---|---|
| VEGFR3 (546)<br>Emcn (594)<br>TO-PRO-3 (647) | 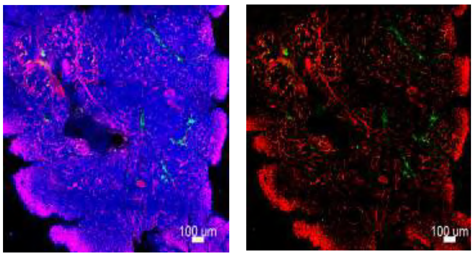 | CD31 (488)<br>Emcn (594)<br>TO-PRO-3 (647) | 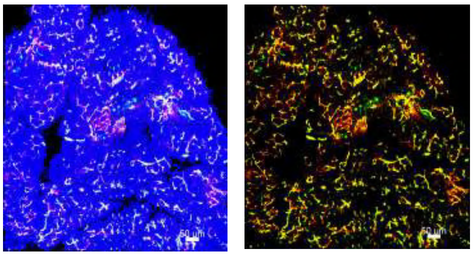 |
| HIF1α (488)<br>Endoglin (546)<br>HSPG2 (594)<br>TO-PRO-3 (647) | 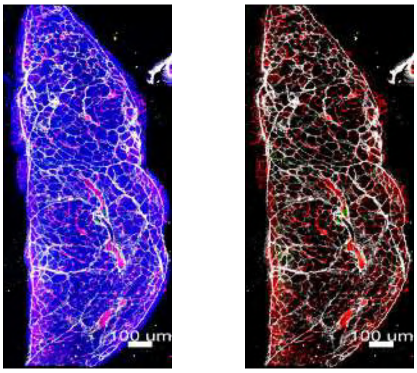 | Endoglin(546)<br>F4/80 (594)<br>TO-PRO-3 (647) | 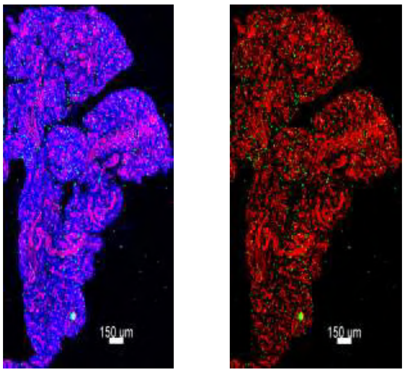 |
| CD31(488)<br>PDGFRβ (546)<br>Emcn (594)<br>TO-PRO-3 (647) | 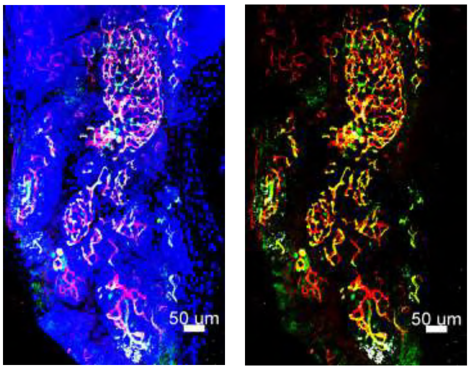 | Endoglin (546)<br>Emcn (594)<br>TO-PRO-3 (647) | 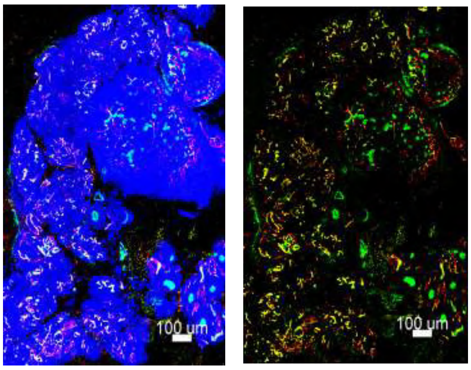 |
| VEGFR3(488)<br>Emcn (594)<br>TO-PRO-3 (647) | 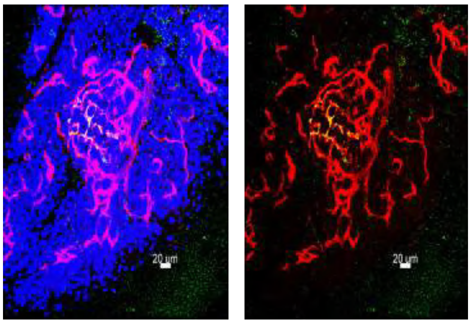 | CD31(488)<br>Emcn (594)<br>TO-PRO-3 (647) | 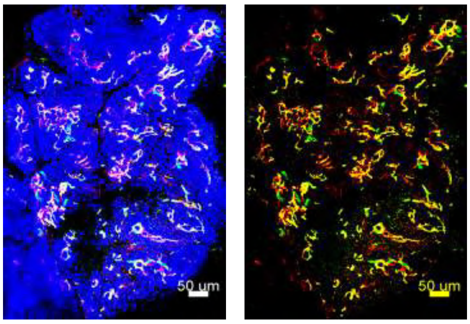 |
| ESM-1 (546)<br>VE-Cadherin (546)<br>TO-PRO-3 (647) | 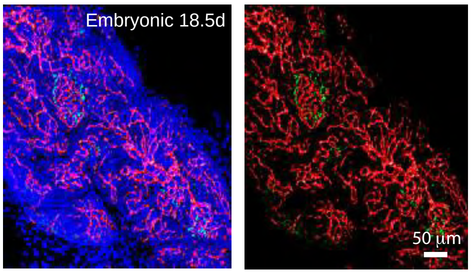 | ESM-1 (546)<br>VE-Cadherin (546)<br>TO-PRO-3 (647) | 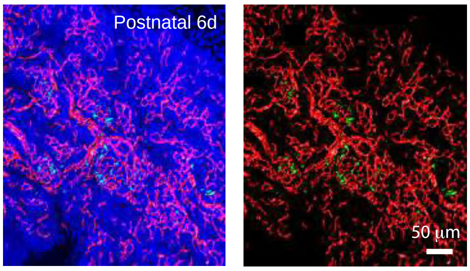 |

| Antibodies | Aged | Antibodies | Aged |
| --- | --- | --- | --- |
| HIF1α (488)<br>Decorin (546)<br>Emcn (594)<br>TO-PRO-3 (647) | 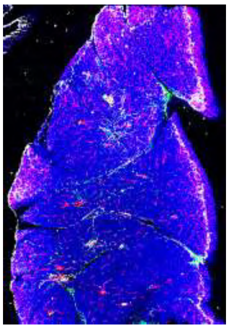 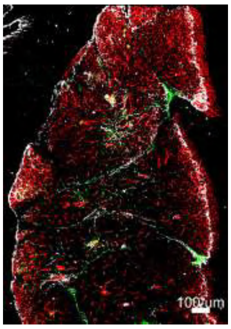 | Fibronectin (488)<br>Endoglin (546)<br>HSPG2 (594)<br>TO-PRO-3 (647) | 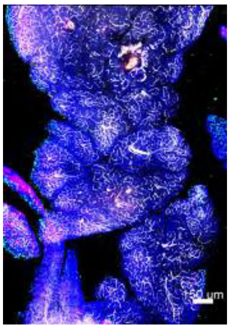 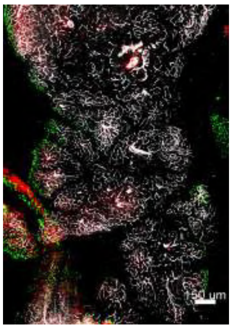 |

**Appendix Table S11** Single-cell 3D mapping image database of young versus aged murine thyroid glands

| Antibodies | Young | Aged | Database No. |
|---|---|---|---|
| Fibronectin (488)<br>Endoglin (546)<br>CD68 (594)<br>TO-PRO-3 (647) | 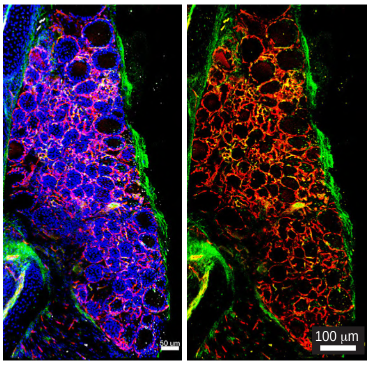 | 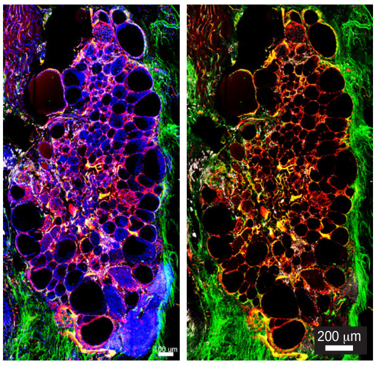 | 1 |
| Collagen IV (488)<br>c-kit (546)<br>Emcn (594)<br>TO-PRO-3 (647) | 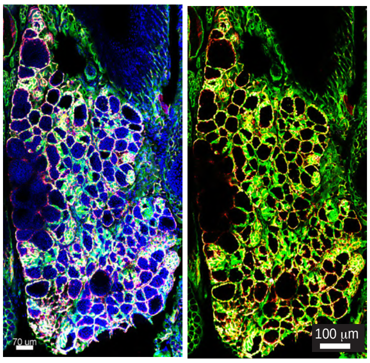 | 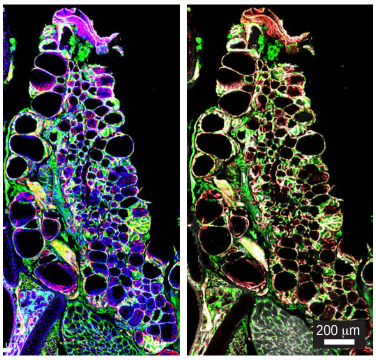 | 2 |
| Perilipin (488)<br>FABP4 (546)<br>Emcn (594)<br>TO-PRO-3 (647) | 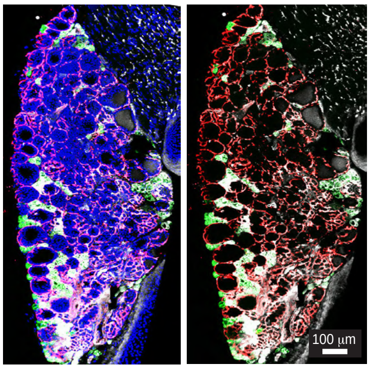 | 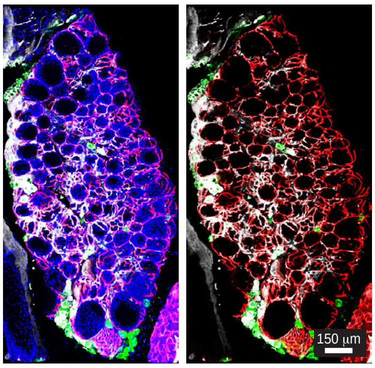 | 3 |
| Smoothelin (488)<br>BCAM (546)<br>Emcn (594)<br>TO-PRO-3 (647) | 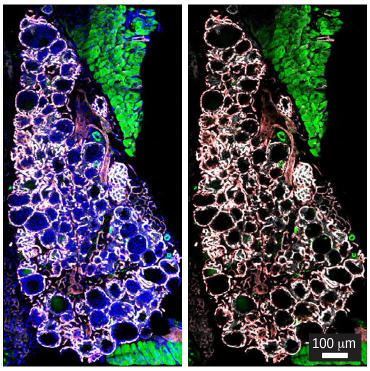 | 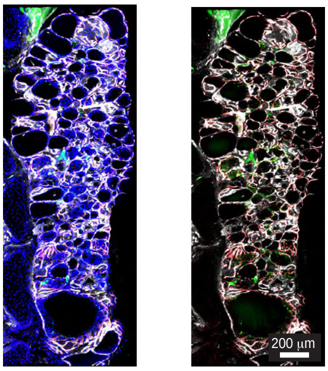 | 4 |
| PDGFRβ (488)<br>Podoplanin (546)<br>Emcn (594)<br>TO-PRO-3 (647) | 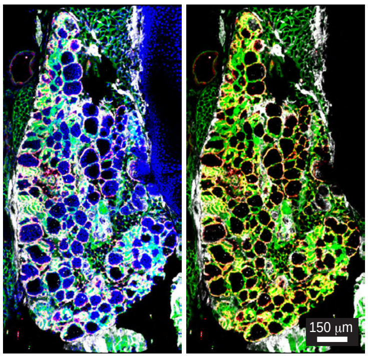 | 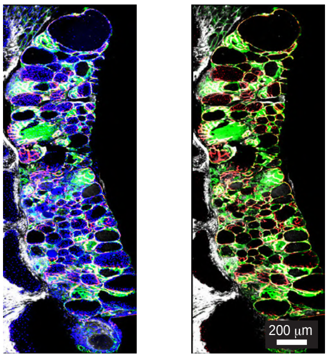 | 5 |

| Antibodies | Young | Aged | Database No. |
| --- | --- | --- | --- |
| Caveolin-1 (488)<br>Endoglin (546)<br>F4/80 (594)<br>TO-PRO-3 (647) | 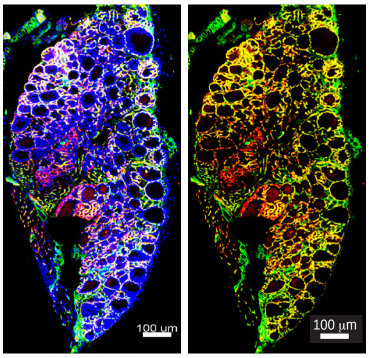  | 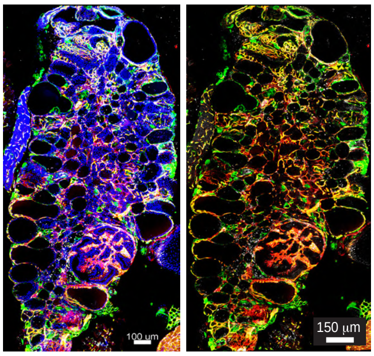  | 6 |
| Laminin (488)<br>CXCR4 (546)<br>Emcn (594)<br>TO-PRO-3 (647) | 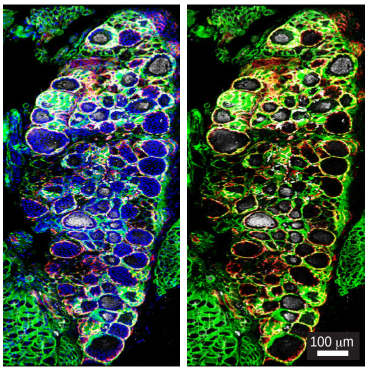  | 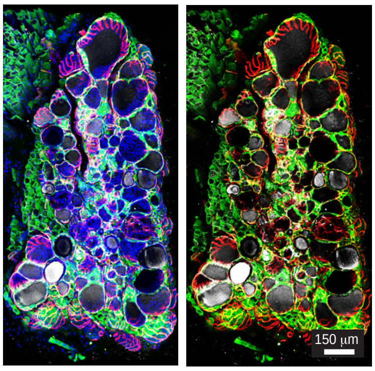  | 7 |
| GJA-1 (488)<br>BCAM (546)<br>CD102 (594)<br>TO-PRO-3 (647) | 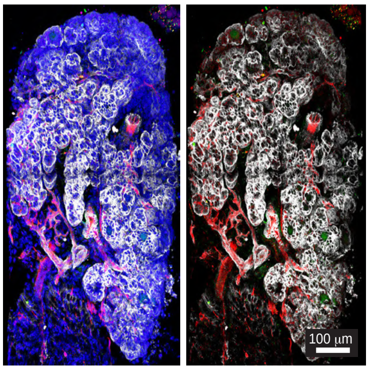  | 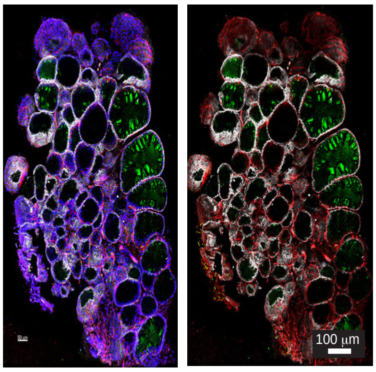  | 8 |
| Endoglycan (546)<br>HSPG2 (594)<br>TO-PRO-3 (647) | 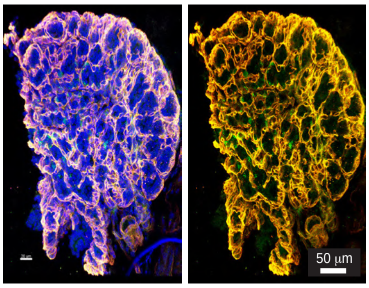  | 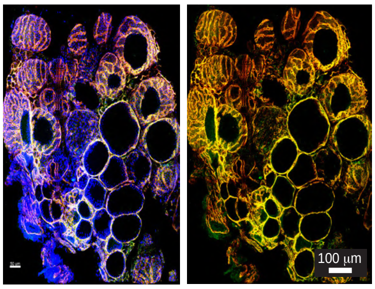  | 9 |
| EPCR (488)<br>α-SMA (546)<br>HSPG2 (594)<br>TO-PRO-3 (647) | 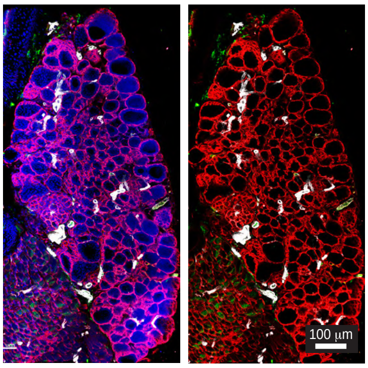  | 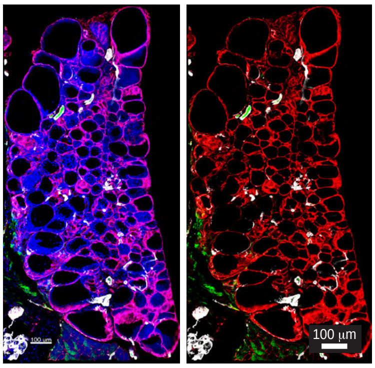  | 10 |

| Antibodies | Young | Aged | Database No. |
|---|---|---|---|
| eIF2α (488)<br>PDGFRα (546)<br>HSPG2 (594)<br>TO-PRO-3 (647) | 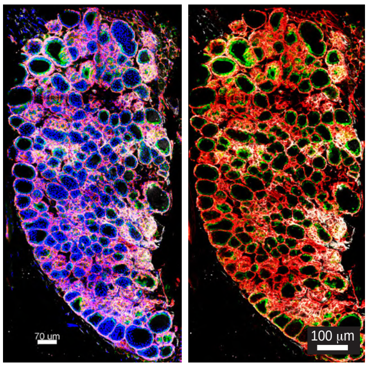 | 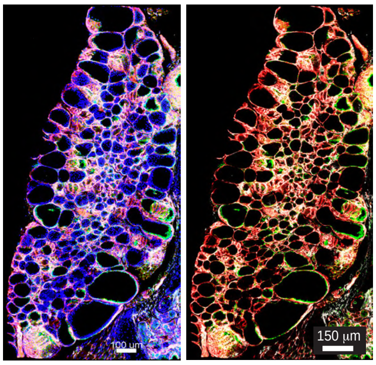 | 11 |
| Vimentin (488)<br>PDGFRα (546)<br>HSPG2 (594)<br>TO-PRO-3 (647) | 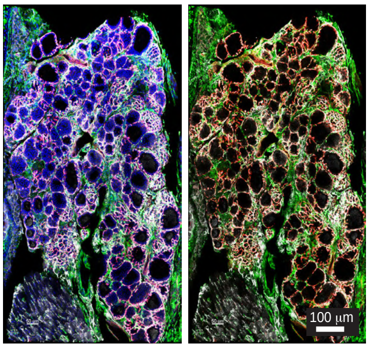 | 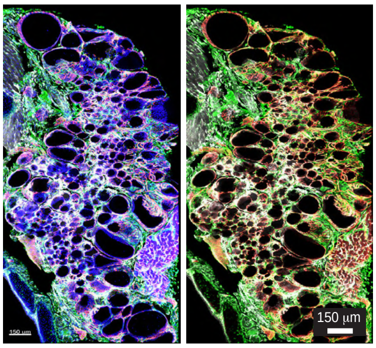 | 12 |
| NG2 (488)<br>BCAM (546)<br>Emcn (594)<br>TO-PRO-3 (647) | 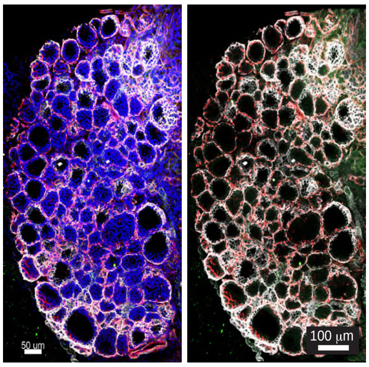 | 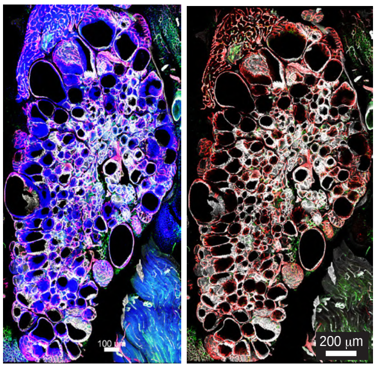 | 13 |
| Claudin-5 (488)<br>Dll4 (546)<br>Emcn (594)<br>TO-PRO-3 (647) | 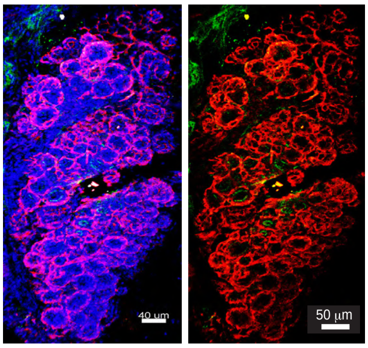 | 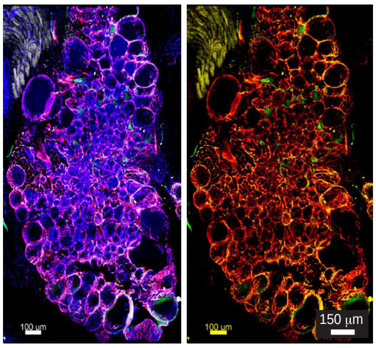 | 14 |
| HIF1α (488)<br>Endoglin (546)<br>HSPG2 (594)<br>TO-PRO-3 (647) | 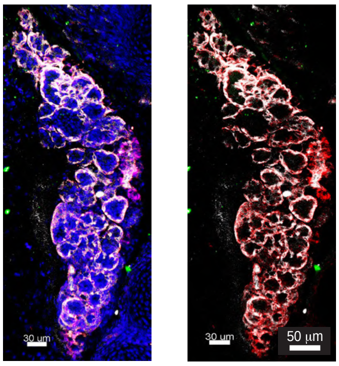 | 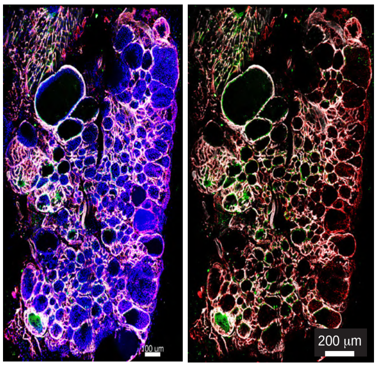 | 15 |

| Antibodies | Young | Aged | Database No. |
|---|---|---|---|
| HIF1α (488)<br>Decorin (546)<br>Emcn (594)<br>TO-PRO-3 (647) | 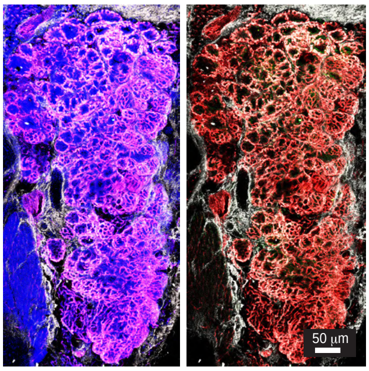 | 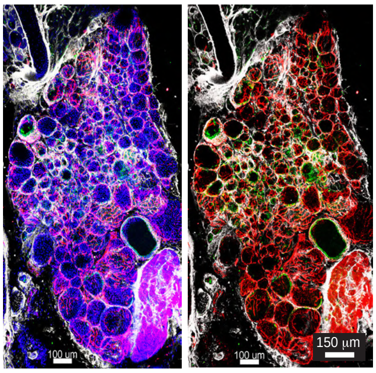 | 16 |
| FSP1 (488)<br>Isolectin (546)<br>CD34 (594)<br>Endoglin (647)<br>DAPI (405) | 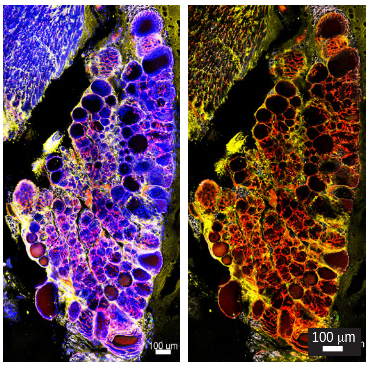 | 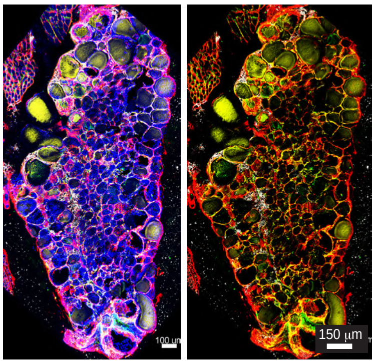 | 17 |
| FSP1 (488)<br>Isolectin (546)<br>CD102 (594)<br>Endoglin (647)<br>DAPI (405) | 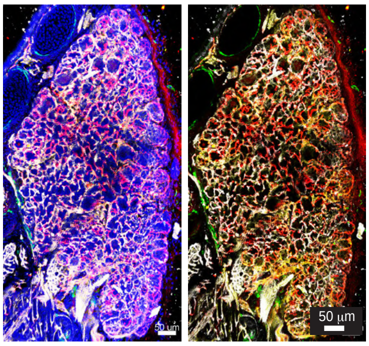 | 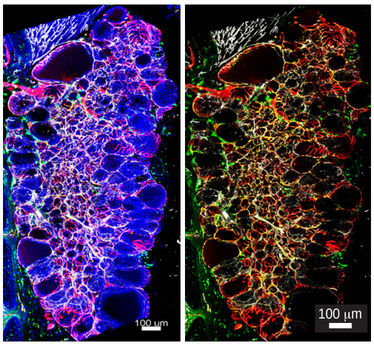 | 18 |
| GJA-1 (488)<br>Tie-2 (546)<br>Emcn (594)<br>TO-PRO-3 (647) | 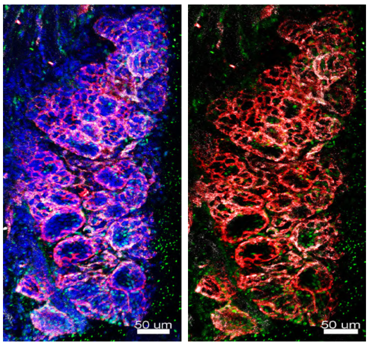 | 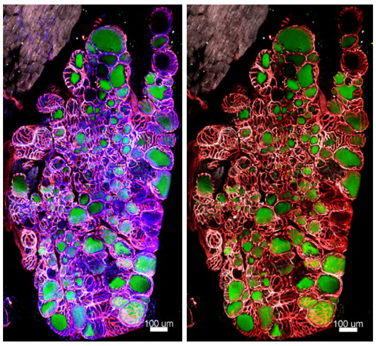 | 19 |
| CD31 (488)<br>Emcn (594)<br>TO-PRO-3 (647) | 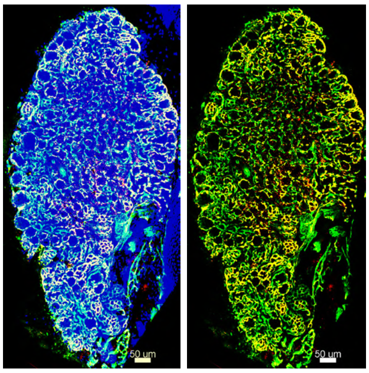 | 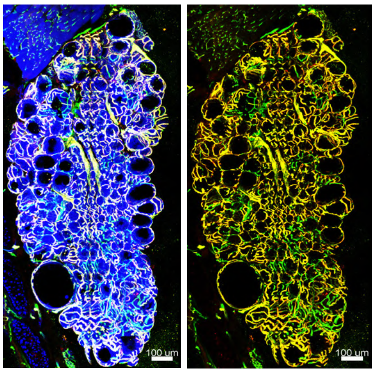 | 20 |

| Antibodies | Young | Aged | Database No. |
|---|---|---|---|
| VEGFA (488)<br>Emcn (594)<br>TO-PRO-3 (647) | 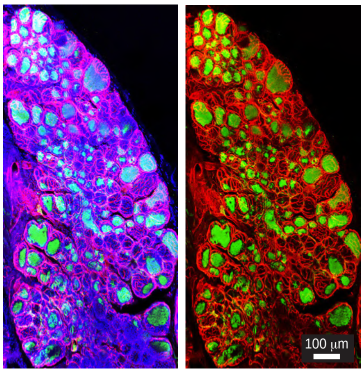 | 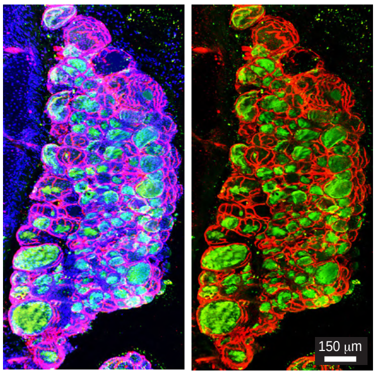 | 114, 115 |

**Appendix Table S12**. Single-cell 3D mapping image database of murine thyroid glands with various niche markers

| Antibodies | Young | Antibodies | Young |
|---|---|---|---|
| Laminin (488)<br>Isolectin (546)<br>CD34 (594)<br>Decorin (647)<br>DAPI (405) | 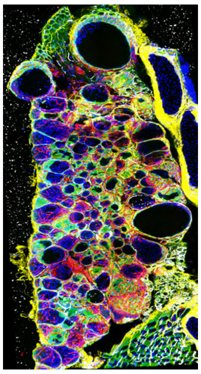 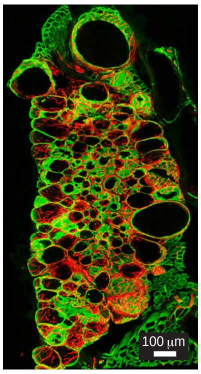 | FSP1 (488)<br>Isolectin (546)<br>Endoglin (594)<br>Decorin (647)<br>DAPI (405) | 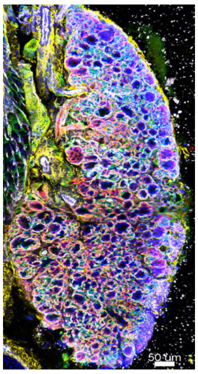 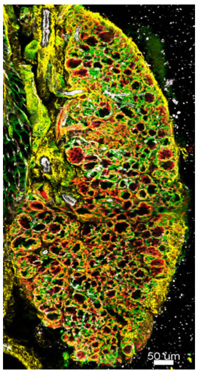 |

| Antibodies | Aged | Antibodies | Aged |
|---|---|---|---|
| Vimentin (488)<br>VCAM-1 (546)<br>Emcn (594)<br>TO-PRO-3 (647) | 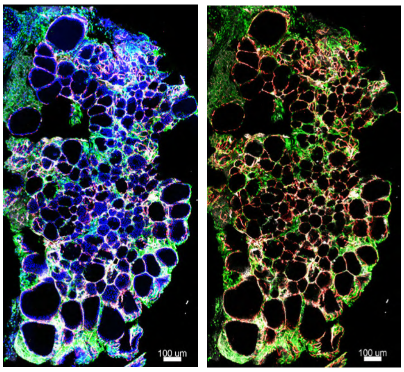 | Collagen IV (488)<br>Sca-1 (546)<br>TO-PRO-3 (405) | 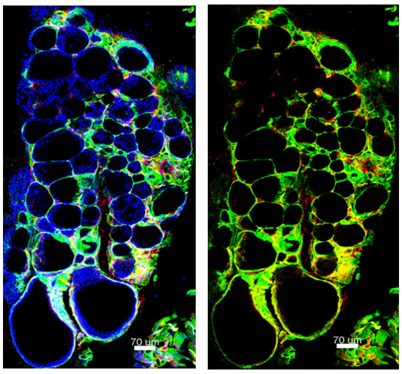 |
| α-SMA (488)<br>Emcn (594)<br>TO-PRO-3 (647) | 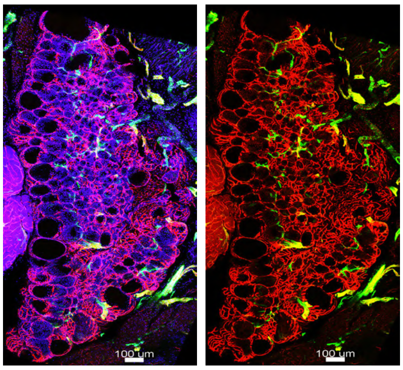 | Endoglin (488)<br>Emcn (594)<br>TO-PRO-3 (647) | 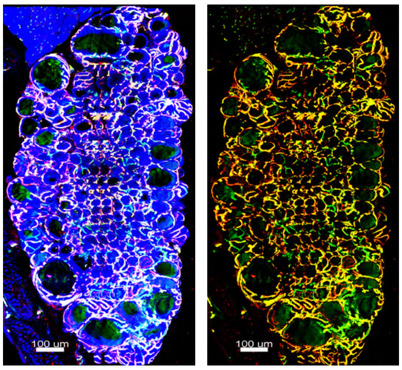 |
| BCAM (488)<br>Emcn (594)<br>TO-PRO-3 (647) | 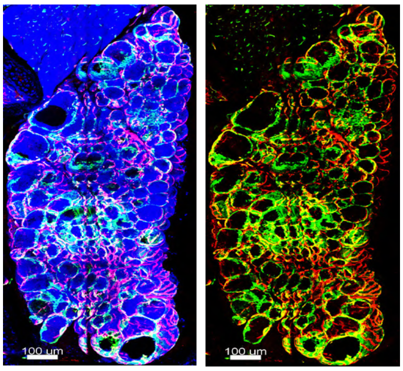 | | |
