# Supplementary material for: Decreased blood vessel density and endothelial cell subset dynamics during ageing of the endocrine system
Source: EMBO J. 2020 Nov 20;40(1):e105242. doi: 10.15252/embj.2020105242 (PMC7780152; doi:10.15252/embj.2020105242)
Supplement: Supplementary file 2 — Expanded View Figures PDF [file EMBJ-40-e105242-s002.pdf]

Expanded View Figures

Figure EV1. Pericytes in young versus aged mouse and human endocrine glands.

A Representative 3D images with PDGFRβ and Emcn expressions in young and aged pancreas. In the inset are represented with higher magnification positive cells for PDGFRβ located in the proximity of blood vessels in periphery and centre regions of the pancreas.
B 3D tile scan images of young and aged thyroid gland stained with PDGFRβ and Emcn in periphery and centre regions of the thyroid gland.
C 3D images of young and aged ovary staining with PDGFRβ and HSPG2 in cortex and medulla regions.
D Tile scan 3D images show PDGFRβ and Emcn in young and aged adrenal gland. Insets with higher magnification areas of cortex and medulla regions of the adrenal gland.
E Representative 3D images with immunostaining for α-SMA, PDGFRβ and Endoglin in young and aged human pancreas.
F 3D images of young and aged human ovary staining with α-SMA, PDGFRβ and CD31.
G 3D images of young and aged human testis after α-SMA, PDGFRβ and Endoglin immunostaining.
H 3D images of young and aged human adrenal gland after α-SMA, PDGFRβ and CD31 immunostaining.
I Representative 3D images with α-SMA, PDGFRβ and CD31 immunostaining in young and aged human thyroid gland.

Data information: The white and dashed lines in panels (A-D) represent the outlines of organs. Co, cortex; Ct, centre; Me, medulla; Pe, periphery. Nuclei stained with DAPI. Scale bars are 200 μm for tile scan 3D images and 50 μm for the high magnification insets in panels (A–D); scale bars are 80 μm for the 3D images in panels (E–I).

© 2020 The Authors

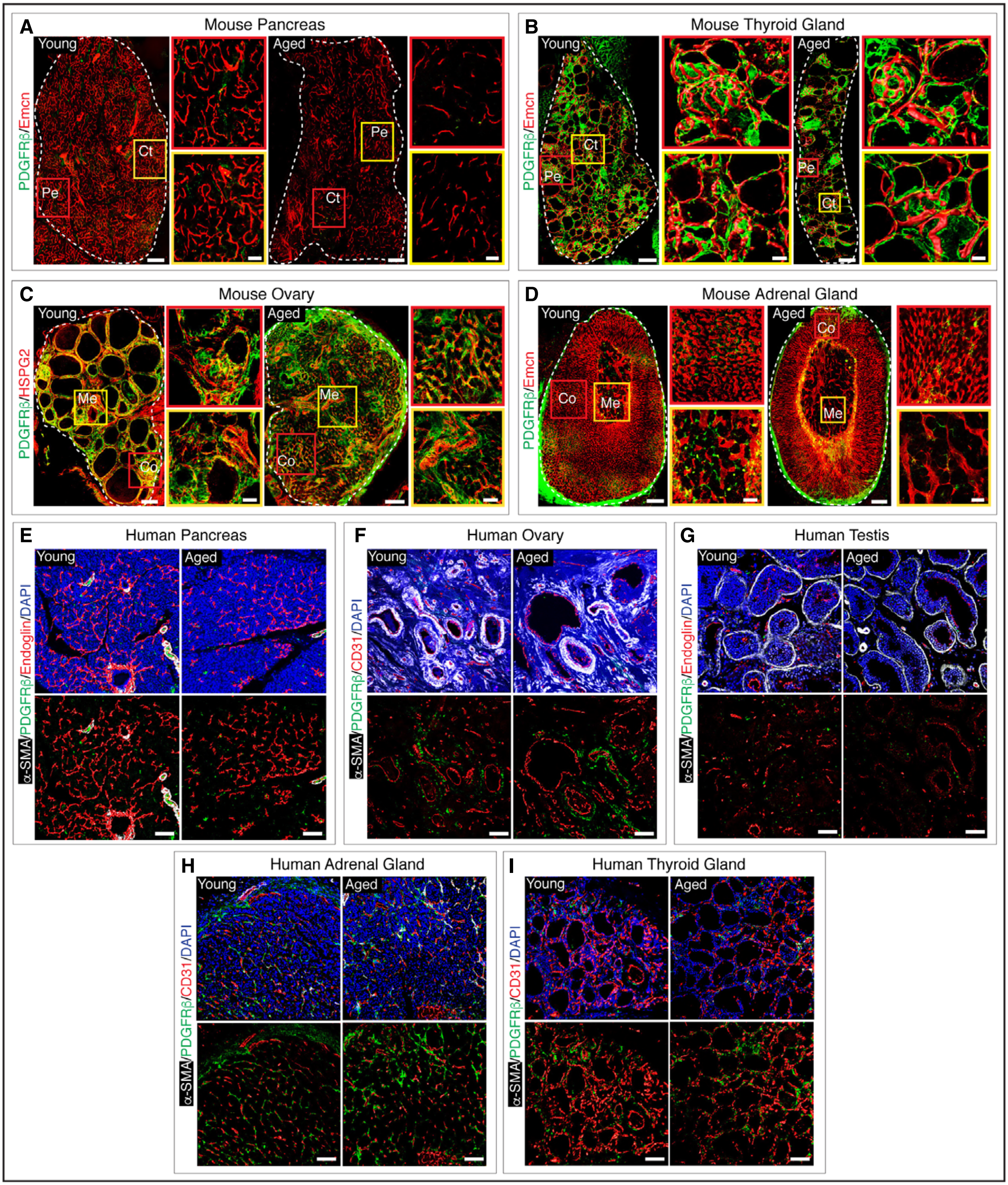

Figure EV1.

 © 2020 The Authors

Figure EV2. Structure and angiogenic nature of islet.

A Representative 3D images show CD31 and Emcn immunostaining in young pancreatic islet. Arrowheads represent the connected capillaries at the interface of the pancreatic islet and exocrine tissue.
B Bar graph with the quantification of capillary diameter inside the pancreatic islets and in the non-islet region of the young pancreas. Data represent mean ± s.d. (*n* = 5), *P*-value and two-tailed unpaired *t*-test.
C 3D images of young pancreas staining with ESM-1 and Emcn. Inset (*) shows higher magnifications with a surface rendering for ESM-1 and Emcn immunostaining.
D Images with insulin, α-SMA and Emcn in the adult pancreas.
E Bar graph with the percentage of ESM-1^+ pancreatic islets in adult (17-week-old) and aged mice. Data represent mean ± s.d. (*n* = 4), *P*-value and two-tailed unpaired *t*-test.
F Bar graph with the quantification of the percentage of VEGFA per islet (f.u) in ESM-1^+ and ESM-1^- pancreatic islets from adult mice. Data represent mean ± s.d. (*n* = 5), *P*-value and two-tailed unpaired *t*-test.
G Representative 3D images with the VEGFA expression in the ESM-1^+ and ESM-1^- pancreatic islets from 17-week-old mice. Insets (* and #) show higher magnifications with surface rendering for ESM-1, VEGFA and Emcn immunostaining. Nuclei stained with TO-PRO-3.
H Representative 3D images of young and aged pancreatic islets stained with insulin and PLVAP.
I Representative high magnification 3D images with a surface rendering for ESM-1 and Emcn in sunitinib-treated and control pancreatic islets. Combo plot shows the quantification of ESM-1^+ cell numbers per islet (f.u) counted on thick murine pancreatic sections. (*n* = 5), *P*-values and two-tailed unpaired *t*-tests.
J High magnification images with a surface rendering for ESM-1 and Emcn immunostaining in *Vegfr2*$^{i\Delta EC}$ mutant and control pancreatic islets. Combo plot shows the quantification of ESM-1^+ cell numbers per islet (f.u). (*n* = 5), *P*-values and two-tailed unpaired *t*-tests.
K qPCR analysis of *Pdgfa*, *Pdgfb, Igf1, Igf2, Cxcl12, Hgf* and *Kitl* expression (normalized to *Actb*) in young human pancreas compared to the aged human pancreas. Data represent mean ± s.d. (*n* = 4), *P*-values and two-tailed unpaired *t*-tests.

Data information: The white and dashed lines in panels (A, C, D, G and H) represent the outlines of pancreatic islets. f.u: fluorescence unit. Nuclei stained with TO-PRO-3 or DAPI. Two-tailed Student's *t*-test was performed for the statistical analysis. ***P* < 0.01; ****P* < 0.001; *****P* < 0.0001. For the combo plots in panels (I and J), the boxes represent mean ± s.d., line in the box is the median, and the lower and upper lines show the minimum and the maximum of the values. The line on the right side of the combo plots represents the sample distribution. Scale bars are 50 μm for 3D images and 5 μm for the single-cell resolution image in panels (C, G, I and J).

© 2020 The Authors

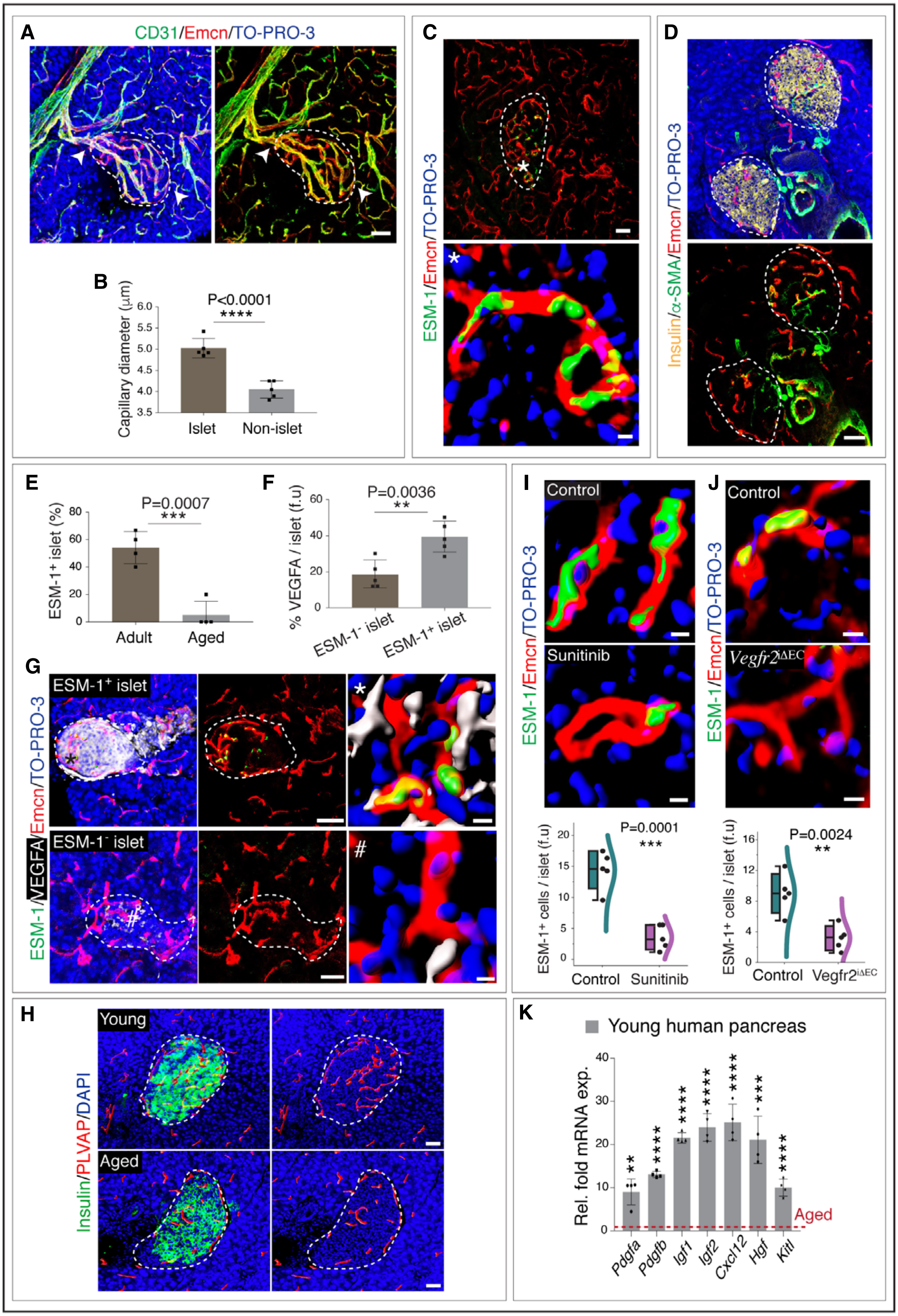

Figure EV2.

 © 2020 The Authors

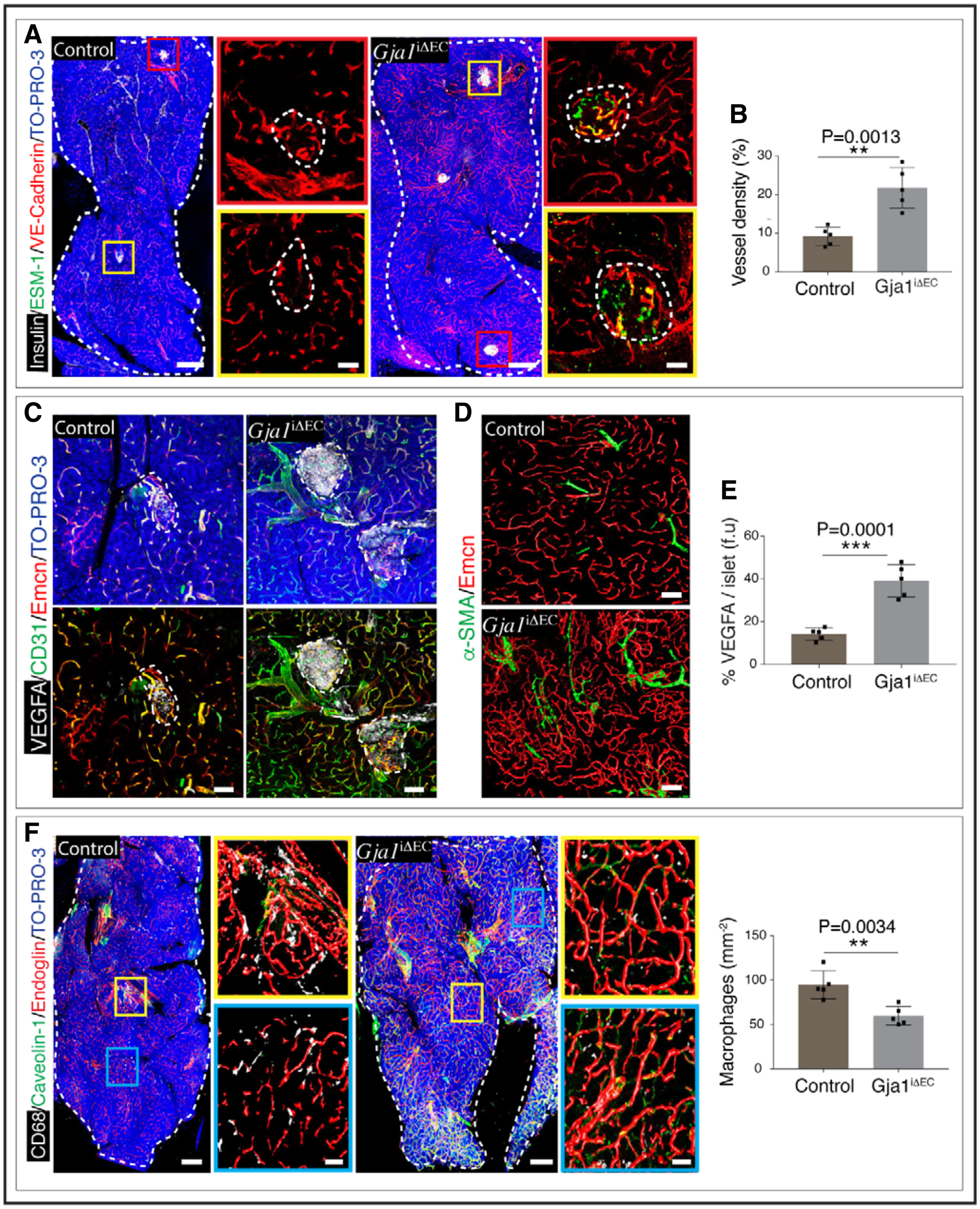

Figure EV3. Role of endothelial Gja1 in pancreatic islets.

A Representative tile scan 3D images with insulin, ESM-1 and VE-Cadherin immunostaining on *Gja1*$^{i\Delta EC}$ and control murine pancreas. Insets show higher magnifications of ESM-1^{+} and ESM-1^{-} pancreatic islets. The white and dashed lines in the tile scan images represent the outlines of one lobe of the pancreas, and the dashed lines in the insets represent the pancreatic islets.

B Bar graph with the quantification of vessel density in *Gja1*$^{i\Delta EC}$ mutant and control pancreas. Data represent mean ± s.d. (n = 5), *P*-value and two-tailed unpaired *t*-test.

C 3D images with VEGFA, CD31 and Emcn staining in *Gja1*$^{i\Delta EC}$ and control pancreas. The scale bars are 50 μm. The dashed lines represent the pancreatic islets.

D Images with α-SMA and Emcn in pancreas from *Gja1*$^{i\Delta EC}$ and littermate control mice. The scale bars are 50 μm.

E Bar graph with the quantification of the percentage of VEGFA per islet (f.u) in pancreas from *Gja1*$^{i\Delta EC}$ and littermate control mice. Data represent mean ± s.d. (n = 5), *P*-values and two-tailed unpaired *t*-tests.

F Representative tile scan 3D images with staining of CD68, Caveolin-1 and Endoglin in pancreas from *Gja1*$^{i\Delta EC}$ and littermate control mice. Bar graph with the quantification of macrophage numbers in pancreas from *Gja1*$^{i\Delta EC}$ and control mice. Data represent mean ± s.d. (n = 5), *P*-value and two-tailed unpaired *t*-test. The white and dashed lines represent the outlines of the pancreas.

Data information: Two-tailed Student's *t*-tests were performed for the statistical analysis. Nuclei: TO-PRO-3. $^{**}P < 0.01$; $^{***}P < 0.001$. f.u: fluorescence unit. Scale bars are 200 μm for longitudinal tile scans of the glands and 50 μm for higher magnification images.

© 2020 The Authors

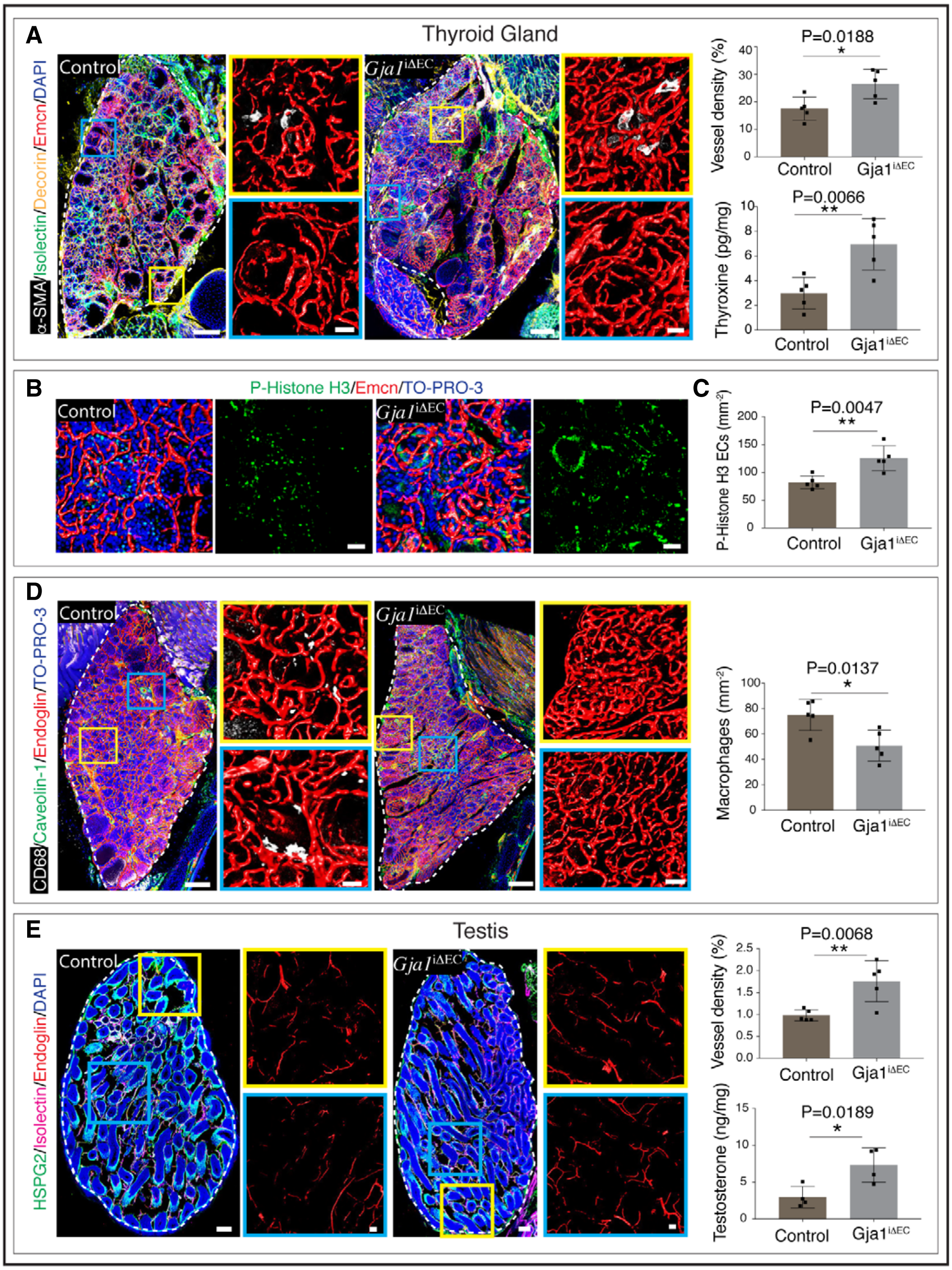

Figure EV4.

© 2020 The Authors

Figure EV4. Role of endothelial Gja1 in thyroid gland and testis.

A Representative tile scan 3D images with staining for α-SMA, Isolectin, Decorin and Emcn in adult (16 weeks old) thyroid gland from *Gja1*$^{i\Delta EC}$ and littermate control mice. Bar graph (top right panel) with the quantification of vessel density in *Gja1*$^{i\Delta EC}$ mutant and control thyroid gland. Bar graph (bottom right panel) with the level of thyroxine in the lysate of thyroid glands from *Gja1*$^{i\Delta EC}$ and control mice determined by ELISA. Data represent mean ± s.d. (n = 5), P-values and two-tailed unpaired t-tests.

B 3D images with P-Histone H3 and Emcn staining in *Gja1*$^{i\Delta EC}$ and control thyroid gland. The scale bars are 50 μm.

C Bar graph with the quantification of P-Histone H3^{+} EC numbers per 1mm^2 sample areas in thyroid glands from *Gja1*$^{i\Delta EC}$ and littermate control mice. Data represent mean ± s.d. (n = 5), P-value and two-tailed unpaired t-test.

D Representative tile scan 3D images with staining of CD68, Caveolin-1 and Endoglin in thyroid gland from *Gja1*$^{i\Delta EC}$ and littermate control mice. Bar graph with the quantification of macrophage numbers in *Gja1*$^{i\Delta EC}$ and control thyroid gland. Data represent mean ± s.d. (n = 5), P-value and two-tailed unpaired t-test.

E Representative tile scan 3D images with staining of HSPG2, Isolectin and Endoglin in testis from *Gja1*$^{i\Delta EC}$ and littermate control mice. Bar graph (top right panel) with the quantification of vessel density in *Gja1*$^{i\Delta EC}$ mutant and control testis. Data represent mean ± s.d. (n = 5), P-value and two-tailed unpaired t-test. Bar graph (bottom right panel) with the levels of testosterone in the lysate of testes from *Gja1*$^{i\Delta EC}$ and control mice determined by ELISA. Data represent mean ± s.d. (n = 4), P-value and two-tailed unpaired t-test.

Data information: The white and dashed lines in panels (A, D and E) represent the outlines of organs. Nuclei: DAPI or TO-PRO-3 as indicated. Two-tailed Student's t-tests were performed for the statistical analysis. *P < 0.05; **P < 0.01. Scale bars are 200 μm for tile scan 3D images and 50 μm for higher magnification images.

© 2020 The Authors

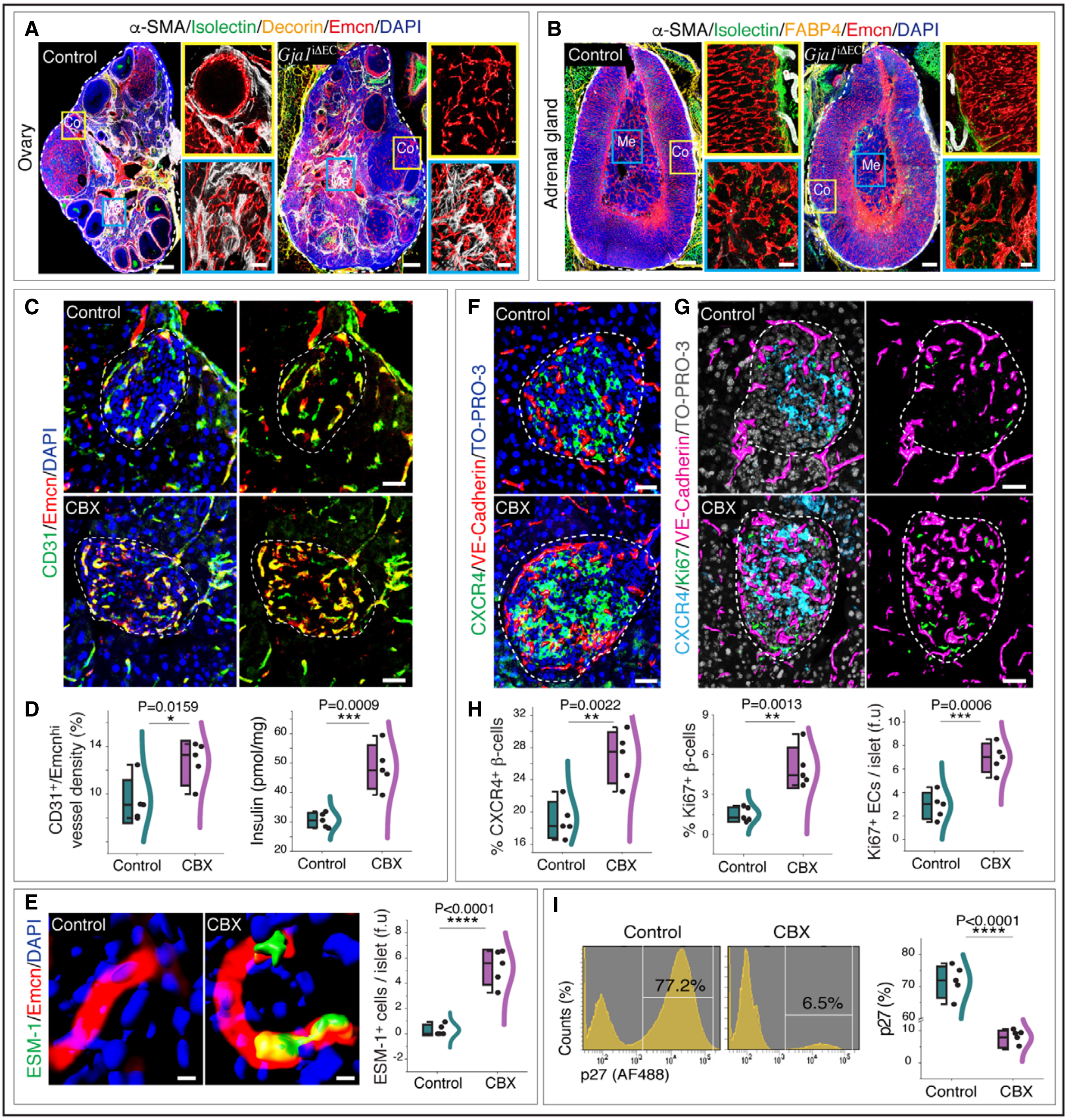

Figure EV5.

 © 2020 The Authors

Figure EV5. Role of endothelial Gja1 in ovary and adrenal glands and the function of Gja1 inhibitor on aged pancreatic islets.

A Representative tile scan 3D images with staining of α-SMA, Isolectin, Decorin and Emcn in the ovary from *Gja1*$^{i\Delta EC}$ and littermate control mice.
B Representative 3D images of adrenal gland from *Gja1*$^{i\Delta EC}$ mutant and control mice with α-SMA, Isolectin, FABP4 and Emcn immunostaining.
C Representative 3D images of pancreas from aged carbenoxolone (CBX)-treated and control mice with CD31 and Emcn immunostaining.
D Combined box and whiskers, and scatter plot (left) shows the quantification of CD31$^+$ Emcn^{hi} vessel density in aged CBX-treated and control pancreatic islets. Combo plot (right) shows the quantification of insulin in lysates of pancreases determined by ELISA. (n = 5), *P*-values and two-tailed unpaired *t*-tests.
E Representative high magnification 3D images with surface rendering for ESM-1 and Emcn immunostaining in aged CBX-treated and control pancreatic islets. Combo plot demonstrates the quantification of ESM-1$^+$ cell numbers per islet (f.u) in aged CBX-treated and control mice. (n = 5), *P*-value and two-tailed unpaired *t*-test.
F Representative 3D images illustrate CXCR4 and VE-Cadherin immunostaining on pancreas sections from aged CBX-treated and control mice.
G Representative 3D images show CXCR4, Ki67 and VE-Cadherin in aged CBX-treated and control pancreatic islet.
H Combo plots illustrate the quantifications of the percentage of CXCR4$^+$ β cells per islet, Ki67$^+$ β cells normalized to the total number of β cells per islet and Ki67$^+$ EC numbers per islet (f.u) in pancreas from aged CBX-treated and control mice. (n = 5), *P*-values and two-tailed unpaired *t*-tests.
I Representative flow cytometry plot (left) shows the population of p27 in ECs from aged CBX-treated and control pancreas. Combo plot shows the quantification of p27 (%) in ECs from aged CBX-treated and control pancreas. (n = 5), *P*-value and two-tailed unpaired *t*-test.

Data information: The white and dashed lines in panels (A and B) represent the outlines of the organs. The dashed lines in panels (C, F and G) represent the outlines of pancreatic islets. Two-tailed Student's *t*-tests were performed for the statistical analysis. *$P < 0.05$; **$P < 0.01$; ***$P < 0.001$; ****$P < 0.0001$. Nuclei: DAPI or TO-PRO-3 as indicated. f.u: fluorescence unit. Co, cortex; Me, medulla. For the combo plots, the boxes represent mean ± s.d., line in the box is the median, and the lower and upper lines show the minimum and the maximum of the values. The line on the right side of these combo plots in represents the sample distribution. Scale bars are 200 μm for tile scan images and 50 μm for higher magnifications insets in panels (A and B); scale bars are 50 μm for the 3D images in panels (C, F and G) and 5 μm for the higher magnifications in panel (E).

© 2020 The Authors
