# Supplementary material for: Decreased blood vessel density and endothelial cell subset dynamics during ageing of the endocrine system
Source: EMBO J. 2020 Nov 20;40(1):e105242. doi: 10.15252/embj.2020105242 (PMC7780152; doi:10.15252/embj.2020105242)
Supplement: Supplementary file 8 — Movie EV2 [file EMBJ-40-e105242-s008.zip › Movie_EV2.docx]

**Movie EV2**. 3D volumes of a young ovary stained with FSP1 (green), Endoglin (red) and DAPI (blue)
