# Supplementary material for: Decreased blood vessel density and endothelial cell subset dynamics during ageing of the endocrine system
Source: EMBO J. 2020 Nov 20;40(1):e105242. doi: 10.15252/embj.2020105242 (PMC7780152; doi:10.15252/embj.2020105242)
Supplement: Supplementary file 10 — Movie EV4 [file EMBJ-40-e105242-s010.zip › Movie_EV4.docx]

**Movie EV4**. 3D volumes of a young pituitary gland stained with c-kit (white), Laminin (green), Emcn (red) and TO-PRO-3 (blue)
