# Supplementary material for: Decreased blood vessel density and endothelial cell subset dynamics during ageing of the endocrine system
Source: EMBO J. 2020 Nov 20;40(1):e105242. doi: 10.15252/embj.2020105242 (PMC7780152; doi:10.15252/embj.2020105242)
Supplement: Supplementary file 11 — Movie EV5 [file EMBJ-40-e105242-s011.zip › Movie_EV5.docx]

**Movie EV5**. 3D volumes of a young testis stained with α-SMA (white), Gja-1 (green), Emcn (red) and TO-PRO-3 (blue)
