# Supplementary material for: Decreased blood vessel density and endothelial cell subset dynamics during ageing of the endocrine system
Source: EMBO J. 2020 Nov 20;40(1):e105242. doi: 10.15252/embj.2020105242 (PMC7780152; doi:10.15252/embj.2020105242)
Supplement: Supplementary file 14 — Movie EV8 [file EMBJ-40-e105242-s014.zip › Movie_EV8.docx]

**Movie EV8**. 3D volumes of a whole cleared pancreas stained with α-SMA (green) and Emcn (red)
